# *PSD3* downregulation confers protection against fatty liver disease

**DOI:** 10.1038/s42255-021-00518-0

**Published:** 2022-01-31

**Authors:** Rosellina M. Mancina, Kavitha Sasidharan, Anna Lindblom, Ying Wei, Ester Ciociola, Oveis Jamialahmadi, Piero Pingitore, Anne-Christine Andréasson, Giovanni Pellegrini, Guido Baselli, Ville Männistö, Jussi Pihlajamäki, Vesa Kärjä, Stefania Grimaudo, Ilaria Marini, Marco Maggioni, Barbara Becattini, Federica Tavaglione, Carly Dix, Marie Castaldo, Stephanie Klein, Mark Perelis, Francois Pattou, Dorothée Thuillier, Violeta Raverdy, Paola Dongiovanni, Anna Ludovica Fracanzani, Felix Stickel, Jochen Hampe, Stephan Buch, Panu K. Luukkonen, Daniele Prati, Hannele Yki-Järvinen, Salvatore Petta, Chao Xing, Clemens Schafmayer, Elmar Aigner, Christian Datz, Richard G. Lee, Luca Valenti, Daniel Lindén, Stefano Romeo

**Affiliations:** 1 Department of Molecular and Clinical Medicine, Institute of Medicine, Sahlgrenska Academy, Wallenberg Laboratory, University of Gothenburg, Gothenburg, Sweden; 2 Bioscience Metabolism, Research and Early Development Cardiovascular, Renal and Metabolism (CVRM) BioPharmaceuticals R&D, AstraZeneca, Gothenburg, Sweden; 3 Ionis Pharmaceuticals, Carlsbad, CA USA; 4 Bioscience Cardiovascular, Research and Early Development Cardiovascular, Renal and Metabolism (CVRM) BioPharmaceuticals R&D, AstraZeneca, Gothenburg, Sweden; 5 Pathology, Clinical Pharmacology and Safety Sciences BioPharmaceuticals R&D, AstraZeneca, Gothenburg, Sweden; 6 Translational Medicine, Department of Transfusion Medicine and Hematology, Fondazione IRCCS Ca’ Granda Ospedale Maggiore Policlinico and Department of Pathophysiology and Transplantation, Università degli Studi di Milano, Milan, Italy; 7 Department of Medicine, University of Eastern Finland and Kuopio University Hospital, Kuopio, Finland; 8 Institute of Public Health and Clinical Nutrition, University of Eastern Finland, Kuopio, Finland; 9 Clinical Nutrition and Obesity Centre, Kuopio University Hospital, Kuopio, Finland; 10 Department of Pathology, University of Eastern Finland and Kuopio University Hospital, Kuopio, Finland; 11 Section of Gastroenterology and Hepatology, PROMISE, University of Palermo, Palermo, Italy; 12 Department of Pathology, Fondazione Ca’ Granda Ospedale Maggiore Policlinico, Milan, Italy; 13 Antibody Discovery and Protein Engineering (ADPE), AstraZeneca, Cambridge, UK; 14 Discovery Biology, Discovery Sciences R&D, AstraZeneca, Gothenburg, Sweden; 15 University of Lille, Inserm, Lille Pasteur Institute, CHU Lille, European Genomic Institute for Diabetes, U1190 Translational Research in Diabetes, Lille University, Lille, France; 16 CHU Lille, Department of General and Endocrine Surgery, Intergrated Center for Obesity, Lille, France; 17 General Medicine and Metabolic Diseases, Fondazione IRCCS Ca’ Granda Ospedale Maggiore Policlinico, Milan, Italy; 18 Department of Pathophysiology and Transplantation, Università degli Studi di Milano, Milan, Italy; 19 Department of Gastroenterology and Hepatology, University Hospital of Zurich, Zurich, Switzerland; 20 Medical Department 1, University Hospital Dresden, Technische Universitaät Dresden (TU Dresden), Dresden, Germany; 21 Department of Medicine, University of Helsinki and Helsinki University Central Hosptial, Helsinki, Finland; 22 Minerva Foundation Institute for Medical Research, Helsinki, Finland; 23 Department of Internal Medicine, Yale University, New Haven, CT USA; 24 McDermott Center for Human Growth and Development University of Texas Southwestern Medical Center, Dallas, TX USA; 25 Department of General, Visceral, Vascular and Transplantation Surgery, University of Rostock, Rostock, Germany; 26 First Department of Medicine, Paracelsus Medical University, Salzburg, Austria; 27 Department of Internal Medicine, General Hospital Oberndorf, Teaching Hospital of the Paracelsus Medical University Salzburg, Oberndorf, Austria; 28 Division of Endocrinology, Department of Neuroscience and Physiology, Sahlgrenska Academy, University of Gothenburg, Gothenburg, Sweden; 29 Department of Cardiology, Sahlgrenska University Hospital, Gothenburg, Sweden; 30 Clinical Nutrition Unit, Department of Medical and Surgical Sciences, University Magna Graecia, Catanzaro, Italy

**Keywords:** Non-alcoholic fatty liver disease, Non-alcoholic steatohepatitis, Metabolic syndrome, Metabolism

## Abstract

Fatty liver disease (FLD) is a growing health issue with burdening unmet clinical needs. FLD has a genetic component but, despite the common variants already identified, there is still a missing heritability component. Using a candidate gene approach, we identify a locus (rs71519934) at the Pleckstrin and Sec7 domain-containing 3 (*PSD3*) gene resulting in a leucine to threonine substitution at position 186 of the protein (L186T) that reduces susceptibility to the entire spectrum of FLD in individuals at risk. *PSD3* downregulation by short interfering RNA reduces intracellular lipid content in primary human hepatocytes cultured in two and three dimensions, and in human and rodent hepatoma cells. Consistent with this, *Psd3* downregulation by antisense oligonucleotides in vivo protects against FLD in mice fed a non-alcoholic steatohepatitis-inducing diet. Thus, translating these results to humans, *PSD3* downregulation might be a future therapeutic option for treating FLD.

## Abstract (web summary)

Employing a candidate gene approach, Mancina et al. identify a genetic variant of the Pleckstrin and Sec7 domain-containing 3 (*PSD3*) gene that reduces susceptibility to fatty liver disease. Functional studies in vitro and in vivo demonstrate that targeting *PSD3* protects against fatty liver disease.

## Main

Fatty liver disease (FLD) is a growing health issue and is already the leading cause of liver damage worldwide^1^. Liver fat accumulation is the hallmark of FLD, which covers a spectrum of conditions ranging from simple steatosis to liver inflammation and fibrosis^2^. Progression of FLD to advanced fibrosis may result in cirrhosis and hepatocellular carcinoma.

The worldwide prevalence of severe liver fibrosis related to FLD is expected to double within the next 10 years and become the leading cause of liver failure and cancer, leading to a major threat to public health^3^. Despite promising results obtained with drugs targeting dysmetabolism, no drug has yet been approved for the treatment of FLD^4–6^. Therefore, novel effective therapies and non-invasive biomarkers are urgently needed.

FLD is a highly heritable trait, with an estimated heritability of between 25% and 60%^7–11^. Genetic variations in Patatin-like phospholipase domain-containing protein 3 (*PNPLA3*) and other lipid droplet-related genes contribute to an increased risk of FLD onset and progression^12–16^. These genetic variations affect hepatic lipid handling in the liver by altering lipid secretion, lipid droplet remodelling or by increasing de novo lipogenesis, and result in liver inflammation and fibrosis^17^. Despite recent progress in the field, the common variants identified to date account for less than 6% of the disease variability^17^.

To identify additional loci affecting hepatic fat content, we used a candidate gene approach starting from gene variants previously identified through a genome-wide association study as determinants of fasting circulating triglycerides^18^. Fasting triglycerides are produced by the liver in the form of very low-density lipoproteins and are a proxy for hepatic fat content^19–23^.

In the discovery phase, we examined the association between variants in the candidate genes and liver fat content in the Dallas Heart Study (DHS) cohort (*n* = 2,736). Next, we validated our results in: (1) the Liver Biopsy Cohort (LBC; *n* = 1,951) consisting of individuals at risk for non-alcoholic fatty liver disease (NAFLD) from northern and southern Europe; (2) participants from the United Kingdom (UK) Biobank cohort for whom liver fat measurements were available (*n* = 10,970); and (3) an independent cohort of individuals with obesity at risk for liver disease from central Europe with liver biopsy available (*n* = 674). To better understand the molecular mechanisms underlying the genetic association, we performed studies in mice, in human primary hepatocytes and in human and rodent hepatoma cells.

We identified a locus, previously not linked to liver fat content in the Pleckstrin and Sec7 domain-containing 3 (*PSD3*) gene protecting against liver fat accumulation, inflammation and fibrosis. Downregulation of this gene conferred protection against the entire spectrum of FLD in mice, and against lipid accumulation in primary human hepatocytes and in human and rodent hepatoma cells.

## Results

### PSD3 sequence variation reduces FLD susceptibility in humans

The study design is shown in Supplementary Fig. 1. To identify previously unknown genetic loci affecting liver fat content, we used a candidate gene approach in the DHS, a population-based sample study with measurement of liver fat content by proton magnetic spectroscopy in 2,736 participants. Specifically, we selected all the tag single-nucleotide polymorphisms (SNPs) identified by a previous genome-wide study on circulating triglyceride levels^18^ (Supplementary Table 1). All missense and nonsense variants in the genes identified by the tag SNPs were then tested for association with liver fat content in the DHS^14^ using linear regression analysis under an additive genetic model adjusted for age, gender and the top four principal components of ancestry. For tag SNPs that were intergenic, missense and nonsense variants in both genes flanking the tag SNP were examined.

We identified three missense variants in three genes present in Europeans (minor allele frequency (MAF) >5% in Europeans from the 1000 Genome Project, Database of Single Nucleotide Polymorphisms (dbSNP), current build 154, released 21 April 2020) that were nominally associated (*P* < 0.050) with liver fat content in the DHS (Table 1).

**Table 1:** Missense variants associated with hepatic triglyceride content in the DHS cohort

Chromosome	Position	rsID	Gene	Amino acid substitution	Nucleotide substitution	N0	N1	N2	MAF^a^	MAF EUR^b^	Median0	Median1	Median2	*P* value	Beta	s.e.
2	27730940	rs1260326	*GCKR*^c^	L446P	G/A	1,515	972	247	27%	41%	3.34	3.68	4.57	0.007	0.03	0.01
8	18872307-08	rs71519934	*PSD3*	L186T	AC/CT	1,907	728	101	17%	33%	3.49	3.74	3.01	0.049	−0.02	0.01
19	19379549	rs58542926	*TM6SF2*	E167K	G/A	2,470	259	7	5%	7%	3.46	4.49	15.70	5.7 × 10^−^^8^	0.12	0.02

Of 32 loci identified by a previous genome-wide association study on circulating triglyceride levels, three missense variants with a MAF >5% in Europeans were nominally associated with hepatic triglyceride content in the DHS cohort. Of these three variants, one was associated with a decrease in hepatic fat content (rs71519934, beta = −0.02), and two were associated with increased hepatic fat content (rs1260326, beta = 0.03 and rs58542926, beta = 0.12). The association was tested by linear regression analysis adjusted for age, gender and the top four principal components of ancestry.

N0, number of individuals homozygote for the major allele; N1, number of individuals heterozygote; N2, number of individuals homozygote for the minor allele; rsID, reference SNP identification.

^a^MAF in the overall DHS cohort. In the DHS, rs71519934 was found as rs7003060, that is, in complete linkage disequilibrium with rs71519934 (*D*′ = 1, *r*^2^ = 1).

^b^MAF EUR refers to 1000 Genome data reported in the dbSNP (current build 154, released 21 April 2020), except for rs71519934 where the frequency was estimated in white British participants from the UK Biobank 50,000 exome sequencing data because data were not available in dbSNP.

^c^Genotyping of *GCKR* was undetermined in two individuals.

Among these, we found two variants robustly associated with FLD and circulating triglycerides, namely the Glucokinase regulator (*GCKR*) rs1260326 (*P* = 0.007) and Transmembrane 6 Superfamily Member 2 (*TM6SF2*) rs58542926 (*P* = 5.7 × 10^−^^8^)^15,24^. Moreover, we found a variant in the *PSD3* gene, rs71519934, associated with lower liver fat content (*P* = 0.049). The DHS consists of three ethnic groups, namely European American, African American and Hispanic American. We next examined differences in clinical and anthropometric parameters, lipoprotein levels and liver fat content stratified by *PSD3* rs71519934 genotype and by these ethnic groups (Supplementary Table 2). In the entire DHS there was no difference in these selected traits among the three genotypes, except for liver fat content. However, when stratified by ethnicity, there was a reduction in circulating total cholesterol levels for the *PSD3* rs71519934 genotype in European Americans.

The *PSD3* rs71519934, in the dbSNP, is annotated as a threonine to leucine amino acid substitution at position 186 of the PSD3 protein (dbSNP, current build 154, released 21 April 2020; https://www.ncbi.nlm.nih.gov/snp/rs71519934). However, exome sequencing of participants from the UK Biobank revealed that the 186 threonine is the minor allele in Europeans (Supplementary Fig. 2).

To test whether the *PSD3* rs71519934 is associated with protection against the entire spectrum of FLD, we examined this variant in the LBC, comprising *n* = 1,951 Europeans at high risk for FLD with liver biopsy available (Supplementary Table 3). In the LBC, the *PSD3* rs71519934 minor allele (186T) was associated with lower prevalence of liver steatosis (*P* = 5.9 × 10^−^^6^), fibrosis (*P* = 0.006), inflammation (*P* = 9.9 × 10^−^^7^) and ballooning (*P* = 0.002) (Table 2) using binary logistic regression analysis under an additive genetic model adjusted for age, gender, body mass index (BMI), centre of recruitment and the *PNPLA3* rs738409.

**Table 2:** *PSD3* rs71519934 minor allele associated with lower prevalence of liver disease in the LBC (*N* histological data = 1,951)

	186L	L186T	186T	*P* value	OR	CI	
*N*	917	796	238				
Steatosis presence, *n* (%)	778 (85)	603 (76)	170 (71)	5.9 × 10^−^^6^	0.67	0.57	0.80
Fibrosis presence, *n* (%)	536 (58)	417 (52)	121 (51)	0.006	0.82	0.72	0.94
Inflammation presence, *n* (%)	596 (65)	420 (53)	118 (50)	9.9 × 10^−^^7^	0.70	0.61	0.81
Ballooning presence, *n* (%)^a^	338 (39)	246 (34)	68 (31)	0.002	0.79	0.68	0.92

The association was tested by binary logistic regression analysis under an additive genetic model adjusted by age, gender, BMI, centre of recruitment and number of *PNPLA3* mutant alleles. Presence of steatosis, fibrosis, inflammation or ballooning was defined as the relative degree >0.

CI, confidence interval; OR, odds ratio.

^a^Data available for *n* = 1,805.

**Fig. 1:**
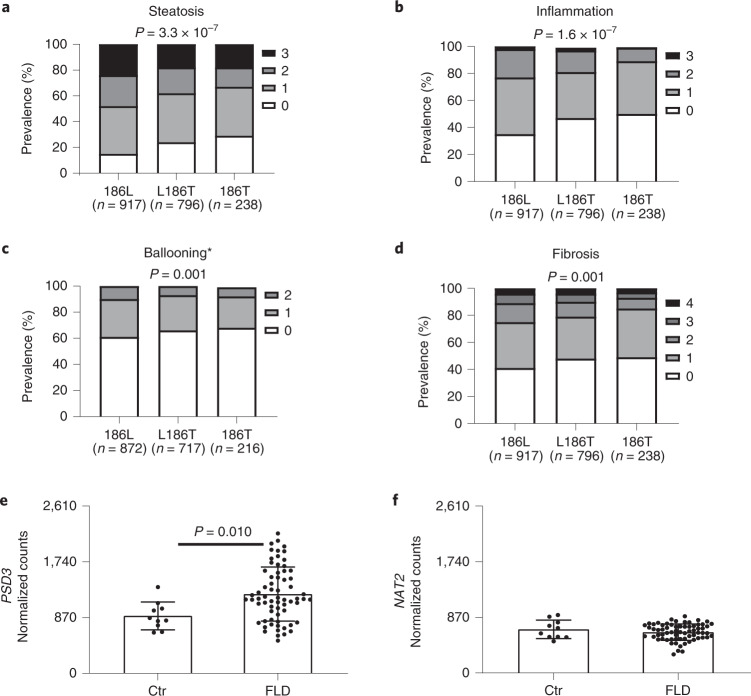
PSD3 minor allele protected against enhanced severity of histological liver damage in the LBC and *PSD3* gene expression was higher in livers with FLD. **a**–**d**, Histological liver damage stratified by PSD3 genotype. Bars show the prevalence expressed as percentage of the degree of the specified disease. Colour shading from white to black indicates increased disease severity. Histological damage was evaluated according to the different components of the NAS and hepatic fibrosis stage. Carriers of the PSD3 rs71519934 186T minor allele had less severe liver disease with lower degrees of steatosis (**a**), inflammation (**b**), ballooning (**c**) and fibrosis (**d**). The association was tested under an additive genetic model by an ordinal regression analysis adjusted for age, gender, BMI, recruitment centre and number of PNPLA3 I148M mutant alleles. Results were virtually the same after further adjustment for the other main genetic risk factors (*TM6SF2* rs58542926 (E167K), *MBOAT7* rs641738 and *GCKR* rs1260326 (L446P)) and environmental (presence of diabetes) variables influencing FLD (Supplementary Table 4). All the reported *P* values are two-sided with no adjustment for multiple testing. *Data available for ballooning, *n* = 1,805. **e**, Total *PSD3* mRNA expression stratified by healthy and FLD livers. Liver *PSD3* expression levels were higher in FLD subjects compared with healthy controls. **f**, Total *NAT2* mRNA expression stratified by healthy and FLD livers. There was no difference in the *NAT2* expression level based on the presence of FLD. **e**,**f**, Data are presented as mean and s.d. Two-sided *P* value was calculated by Mann–Whitney non-parametric test. Ctr, healthy control livers (*n* = 10); FLD, livers with fatty liver disease (*n* = 67). Source data

In addition, carriers of the 186T minor allele were protected against more severe liver steatosis (*P* = 3.3 × 10^−^^7^), inflammation (*P* = 1.6 × 10^−^^7^), ballooning (*P* = 0.001) and fibrosis (*P* = 0.001) (Fig. 1a–d). These results were virtually identical when further adjusted for other genetic (*TM6SF2* rs58542926 (E167K), *MBOAT7* rs641738 and *GCKR* rs1260326 (L446P)) and environmental (presence of diabetes) variables influencing FLD (Supplementary Table 4). Moreover, consistent with European Americans in the DHS, carriers of the *PSD3* 186T minor allele had lower circulating total and low-density lipoprotein (LDL) cholesterol levels (*P* = 1.4 × 10^−^^6^ and *P* = 0.001, respectively; Supplementary Table 5). Next, we tested for an interaction between the *PSD3* and *PNPLA3* variants in the LBC but found no interaction between these two genetic variants and liver disease (Supplementary Fig. 3).

To seek independent replication of the association between the *PSD3* variant and liver fat content, we examined white British participants (*n* = 10,970) from the UK Biobank with liver fat content measured as magnetic resonance imaging (MRI)-derived proton density fat fraction (PDFF). In this population-based study, genetic data on the rs71519934 dinucleotide substitution were not available, therefore we used the rs7003060 which is in complete linkage disequilibrium (*D*′ = 1, *r*^2^ = 1) with the rs71519934 in Europeans. We did not find any association between the *PSD3* minor allele and lower liver fat content in the UK Biobank (Table 3).

**Table 3:** Association of PDFF with *PSD3* rs7003060 overall and within four BMI strata in white British participants from the UK Biobank

rs7003060									
	GG		GT		TT				
	*N*	Median (i.q.r.)	*N*	Median (i.q.r.)	*N*	Median (i.q.r.)	*P* value	Beta	CI
Overall	5,243	2.92 (2.78)	4,668	2.84 (2.71)	1,059	2.86 (2.67)	0.49	−0.00892	−0.034, 0.016
BMI < 25	2,137	2.18 (1.5)	1,907	2.19 (1.40)	442	2.22 (1.36)	0.77	−0.00636	−0.048, 0.035
25 ≤ BMI < 30	2,294	3.32 (2.94)	2,019	3.29 (3.10)	433	3.31 (2.85)	0.66	0.00975	−0.033, 0.052
30 ≤BMI < 35	651	4.86 (6.03)	568	4.25 (4.87)	143	4.72 (6.67)	0.18	−0.0534	−0.13, 0.025
BMI ≥ 35	156	6.37 (8.8)	165	5.14 (8.04)	39	5.60 (7.47)	0.02	−0.175	−0.33, −0.022

Analysis was performed using a linear regression adjusted for age, sex, BMI, the first ten principal components of ancestry and array type.

However, because genetic variations affecting FLD have a robust gene–environment interaction, with BMI amplifying their effect^25^, we examined the differences in liver fat content among the *PSD3* rs7003060 genotype after stratification for severity of overweight/obesity as measured by BMI. Severely obese (BMI > 35) carriers of the *PSD3* minor allele had a lower liver fat content (beta = −0.175, *P* = 0.02; Supplementary Fig. 4 and Table 3).

Considering these results, we further tested the association between the *PSD3* variant and the protection against liver disease in an independent replication cohort of obese (BMI ≥ 30 kg m^−^^2^) central European participants at risk for liver disease^26^. More specifically, we examined 674 adult individuals (mean age 45 ± 12 years), with a mean BMI of 46 ± 10 kg m^−^^2^ from three central European centres (Supplementary Table 3) with liver biopsies available. We found that the *PSD3* minor allele was associated with a lower prevalence of liver steatosis (*P* = 0.024), fibrosis (*P* = 0.049) and ballooning (*P* = 0.047), and with less severe fibrosis and ballooning (*P* = 0.040 and *P* = 0.048, respectively; Table 4). No differences in clinical or metabolic traits were detected in this cohort stratified by *PSD3* genotype (Supplementary Table 5). Finally, we performed a meta-analysis of the LBC and the replication cohort from central Europe. With both fixed and random effect models, the *PSD3* genetic association was stronger for all the traits examined, except for the presence and severity of inflammation where the association was attenuated using a random effect model (Supplementary Table 6).

**Table 4:** *PSD3* rs71519934 minor allele was associated with a lower prevalence and lower severity of liver disease in the central Europeans independent replication cohort (*N* histological data = 674)

	186L	L186T	186T	*P* value	OR	CI	
*N*	330	282	62				
Disease presence							
Steatosis	276 (84)	225 (80)	48 (77)	0.024	0.69	0.50	0.95
Fibrosis	132 (40)	103 (36)	–	0.049	0.77	0.59	1.00
Inflammation	107 (32)	86 (30)	19 (31)	0.524	0.92	0.70	1.20
Ballooning	106 (32)	72 (25)	18 (29)	0.047	0.75	0.56	1.00
Disease severity							
Steatosis				0.158	0.85	0.69	1.06
Fibrosis				0.040	0.77	0.60	0.99
Inflammation				0.355	0.88	0.68	1.15
Ballooning				0.048	0.75	0.57	1.00

The association was tested by binary logistic (disease presence) or ordinal regression (disease severity) analysis under an additive genetic model adjusted by age, gender, BMI, centre of recruitment and number of *PNPLA3* mutant alleles.

The OR for ordinal regression was calculated as exponentials of the coefficient estimate and its CI.

Presence of steatosis, fibrosis, inflammation or ballooning was defined as the relative degree >0.

### PSD3 hepatic expression is increased in individuals with FLD

**Fig. 2:**
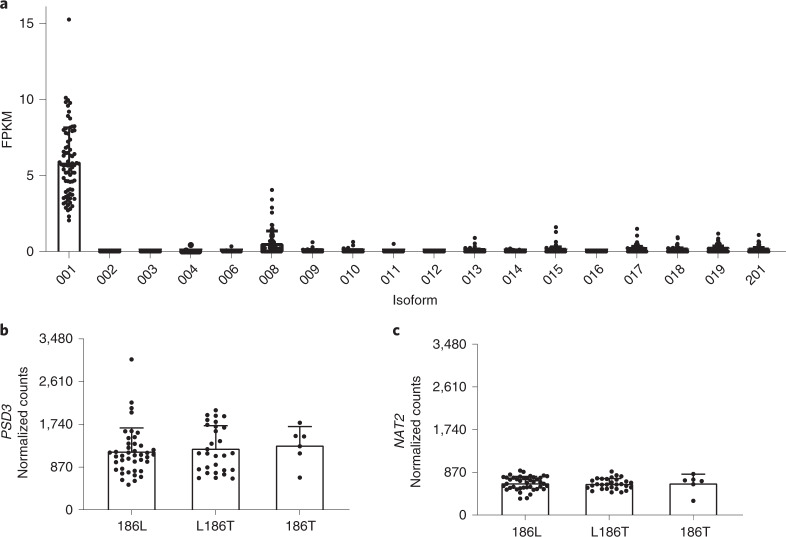
*PSD3* mRNA isoform expression in human liver and total *PSD3* mRNA expression stratified by rs71519934 genotype. **a**, Expression level of the different *PSD3* mRNA isoforms in human liver tissue. To assess which isoform is the most abundant, the transcriptome from liver biopsies of a subset of 77 individuals from the Milan subgroup of the LBC was examined. Isoform 001 (identified as ENST00000327040 by Ensembl or as NP_056125 (isoform-a) by NCBI, 1,047 amino acids) had the highest expression, followed by isoform 008 (identified as ENST00000521841 by Ensembl, non-coding). Data are presented as mean and s.d. **b**, Total *PSD3* mRNA expression stratified by rs71519934 genotype. There were no differences in *PSD3* mRNA expression levels when stratified by genotype. **c**, Total *NAT2* mRNA expression levels did not differ among *PSD3* rs71519934 genotypes. **b**,**c**, Data are presented as mean and s.d. The two-sided *P* value was calculated by unadjusted linear regression. Values were log-transformed before entering the model. FPKM, fragments per kilobase of exon model per million reads mapped; 186L, homozygotes for the L allele (*n* = 42); L186T, heterozygotes (*n* = 29); 186T, homozygotes for the T allele (*n* = 6). Source data

The PSD3 protein has 18 annotated isoforms (Ensembl release 75). To determine which isoform is the most abundant in human liver, we examined the transcriptome of liver biopsies from a subset of individuals (*n* = 77) from the LBC^27^. We found that isoform-a (annotated as NP_056125 in NCBI and as 001 ENST00000327040 in Ensembl) had the highest expression level in the liver (Fig. 2a). Interestingly, the total hepatic *PSD3* messenger RNA levels were higher in livers with FLD than in those without (Fig. 1e), whereas no differences in the *N-acetyltransferase 2* (*NAT2*) mRNA level were observed (Fig. 1f), When we stratified individuals based on the *PSD3* genotype, we found no difference in the mRNA expression level of either *PSD3* or *NAT2* (Fig. 2b,c).

### PSD3 186T protects against intracellular lipid accumulation

**Fig. 3:**
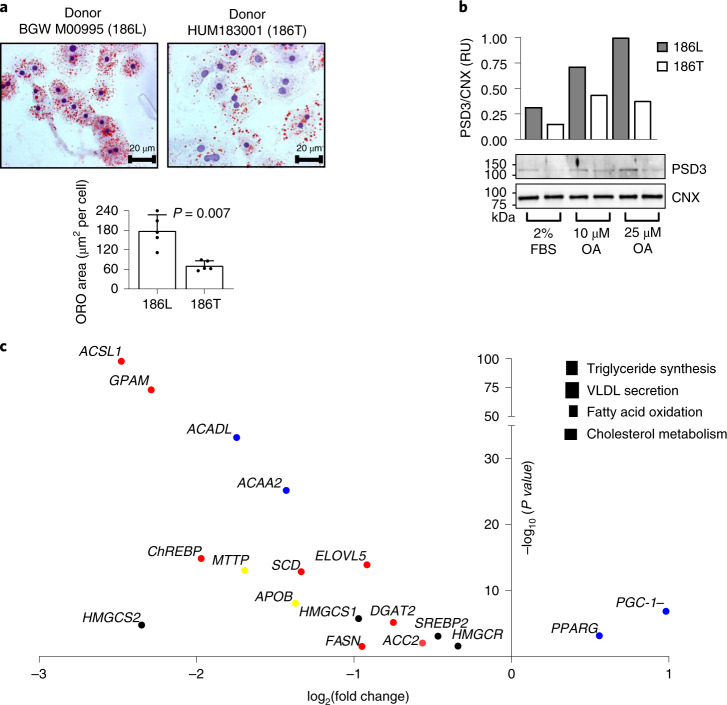
Primary human hepatocytes from a donor homozygous for the 186T allele had lower PSD3 protein and intracellular lipid levels compared with homozygous 186L hepatocytes. Primary human hepatocytes from donors homozygous for 186L or 186T, were cultured in 2D. **a**, Intracellular neutral fat content visualized by ORO staining and quantified by Biopix iQ software v.2.3.1. Data are presented as mean and s.d. of the reported independent experiments (*n* = 5). Two-sided *P* value was calculated by Mann–Whitney non-parametric test. **b**, Cells were cultured in serum-free regular medium supplemented with 2% FBS, 10 µM OA or 25 µM OA for 48 h. Immunoblotting was performed with total cell lysates to detect PSD3 (NCBI: NP_056125, 1,047 amino acids) using a custom antibody. The bar graph shows the relative PSD3 amount calculated as PSD3/calnexin (CNX). **c**, Key genes involved in lipid metabolism that were differentially expressed between donors homozygous for 186T versus 186L obtained with RNA-seq. Data are presented as log_2_(fold change) in expression and –log_10_(*P* value) adjusted using the Benjamini and Hochberg’s approach for controlling the FDR. RU, relative units; VLDL, very low-density lipoprotein. Red Triglyceride synthesis; yellow VLDL secretion; blue Fatty acid oxidation; black cholesterol metabolism. Source data

To understand the mechanism(s) underlying the association between the rs71519934 minor allele and lower liver fat content, we examined primary human hepatocytes from donors carrying the 186L or 186T amino acid change in homozygosity. Consistent with the genetic association, human primary hepatocytes homozygous for the 186T allele cultured in two dimensions (2D) had a lower neutral lipid fat content (*P* = 0.007) as measured by Oil Red O (ORO) staining compared with homozygous 186L hepatocytes (Fig. 3a). To examine PSD3 levels in the two different genotypes, we generated an antibody specific for human PSD3 (custom antibody generation detailed in Methods). We incubated cells with different amount of oleic acid (OA) (0, 10 and 25 µM) and examined protein levels between the two genotypes in primary hepatocytes. Overall, the amount of PSD3 was elevated with increasing concentration of OA (Fig. 3b). However, for each OA concentration, PSD3 protein expression was lower in cells homozygous for the 186T allele. To understand the mechanism behind this association, we examined differentially expressed genes involved in lipid homoeostasis using RNA sequencing (RNA-seq). We found a robust reduction in genes involved in triglyceride synthesis and secretion and cholesterol biosynthesis, whereas the expression of *PGC-1α* involved in mitochondrial biogenesis was increased (Fig. 3c).

### *PSD3* downregulation reduces intracellular lipid accumulation

**Fig. 4:**
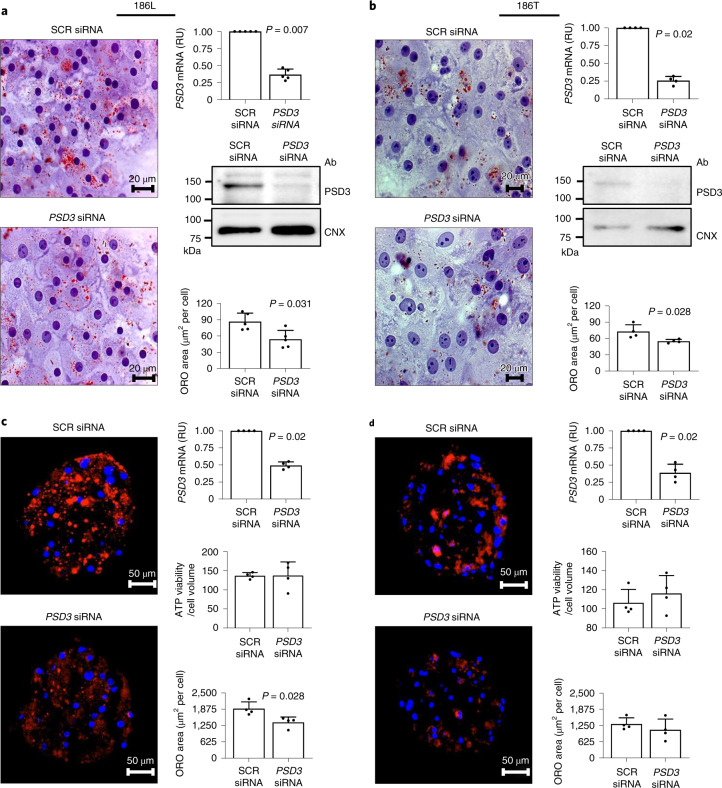
*PSD3* downregulation lowered intracellular neutral fat content in primary human hepatocytes cultured in 2D and 3D spheroids. Downregulation of endogenous *PSD3* expression using siRNA in primary human hepatocytes cultured in 2D and 3D. For 2D culture, after attachment of cells in collagen-coated plates, cells were incubated with regular growth medium supplemented with 10 µM OA and transfected with negative control SCR siRNA or *PSD3* siRNA for 48 h. **a**,**b**, Intracellular neutral fat content was visualized by ORO staining and quantified by Biopix iQ software v.2.3.1 in primary human hepatocytes carrying (**a**) the 186L allele (*n* = 5) or (**b**) the 186T allele (*n* = 4). Average *PSD3* downregulation efficiency was ~80% as evaluated by quantitative retro transcription PCR analysed by the 2^−^^ΔΔCt^ method and western blotting for both donor types. For 3D culture of primary human hepatocytes, spheroids were generated by seeding 2,000 cells per well in a 96-well round-bottom flask, along with transfection mix in 100 µl of medium. For the generation of 186T allele spheroids, 5 nM of FMK-Z-VAD was added to support spheroid formation. After 24 h, additional growth medium was added to give a total volume of 200 µl per well. Fifty per cent of the total media was replenished with fresh media every 48 h. After 7 days of formation, spheroids were collected and 8-µM sections were subjected to ORO staining to visualize intracellular neutral fat content. **c**,**d**, Nuclei were stained with 4,6-diamidino-2-phenylindole and ORO staining was quantified by Image J, normalized to number of nuclei of primary human hepatocyte spheroids carrying (**c**) the 186L allele (*n* = 4) and (**d**) the 186T allele (*n* = 4). Average gene knockdown efficiency was ~50–60% as evaluated by quantitative retro transcription PCR analysed by the 2^−ΔΔCt^ method for both donor types. Cellular ATP levels (marker of viability) remained stable between the negative control SCR and *PSD3* siRNA groups. Data are shown as mean ± s.d. of the reported independent experiments. Two-sided *P* values were calculated by Mann–Whitney non-parametric test comparing SCR siRNA versus *PSD3* siRNA. RU, relative units; CNX, calnexin. Source data

Because *PSD3* expression was elevated in livers from individuals with FLD, we tested the hypothesis that *PSD3* downregulation would result in a reduction in intracellular fat content by using siRNA in human primary hepatocytes from donors homozygous for either the 186L or 186T allele. Downregulation of *PSD3* reduced the intracellular neutral lipid content in hepatocytes carrying either allele when cultured in 2D (Fig. 4a,b). However, *PSD3* silencing reduced intracellular lipid levels only in primary hepatocytes carrying the 186L allele when cultured in a three-dimensional (3D) spheroid model (Fig. 4c,d).

### *PSD3* downregulation reduces triglyceride synthesis and ARF6 activation

**Fig. 5:**
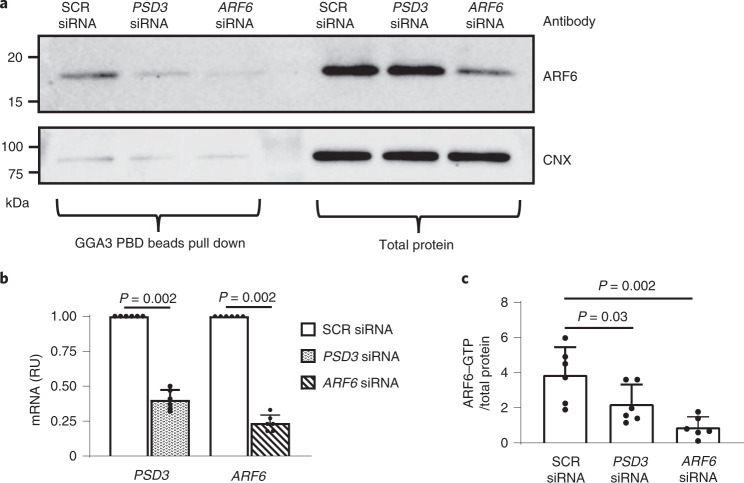
Downregulation of PSD3 resulted in partial loss of ARF6 activation. Human hepatoma Huh7 cells were transiently transfected with negative control SCR siRNA, *PSD3* siRNA or *ARF6* siRNA. Forty-eight hours after transfection, the cell lysates were incubated with GGA3 PBD agarose beads that selectively isolate and pull down endogenous active ARF6 (ARF6–GTP). **a**, After precipitation, active ARF6–GTP was detected by immunoblotting using an anti-ARF6 antibody provided in the kit. Cells transfected with *ARF6* siRNA were used as a control. The experiment was performed independently six times with similar results. Representative blot presented. **b**, Knockdown efficiency with ~60% reduction for PSD3 and ~75% for ARF6 as evaluated by quantitative retro transcription PCR analysed by the 2^−^^ΔΔCt^ method (*n* = 6). **c**, Relative ARF6–GTP (active) calculated as GTP–ARF6/calnexin (CNX) (*n* = 6). Data are shown as mean ± s.d. of the reported independent experiments. Two-sided *P* values calculated by Mann–Whitney non-parametric test. RU, relative units. Source data

To understand the molecular mechanism(s) underlying the genetic association, we examined immortalized hepatocytes from rat (McArdle, McA-RH7777) homozygous for 180T, which corresponds to 186T in human PSD3. In these cells, *Psd3* downregulation resulted in a reduction in the intracellular neutral lipid content as measured by ORO staining. Additionally, *Psd3* downregulation in McA-RH7777 cells resulted in lower triglyceride production, measured as de novo triglyceride synthesis, and as mRNA expression of genes involved in triglyceride synthesis. Moreover, very low-density lipoprotein secretion, measured as apolipoprotein B (Apo B) secretion, was also lower in cells transfected with *Psd3* siRNA compared with scramble (SCR) control siRNA. No differences were detected in intracellular lipid utilization, measured as beta oxidation (Supplementary Fig. 5). To confirm our data, we measured the intracellular lipid accumulation and triglyceride synthesis using radiolabelled tracers in human hepatoma cells (Huh7 cells) homozygous for the *PSD3* 186L allele, and obtained virtually identical results as McA-RH7777 cells (Supplementary Fig. 6). Thus, downregulation of endogenously expressed Psd3 threonine or PSD3 leucine resulted in decreased intracellular lipid levels. PSD3 is a guanine nucleotide exchange factor activating ADP-ribosylation factor 6 (ARF6). To test whether *PSD3* downregulation results in changes in ARF6 activation, *PSD3* was downregulated in Huh7 cells and levels of activated ARF6 were measured using a GGA3 protein-binding domain (PBD) pull-down assay. *PSD3* downregulation resulted in lower levels of activated ARF6 (ARF–GTP) (Fig. 5).

In immunohistochemistry analyses, PSD3 was found to be present at a low level in hepatocytes and there was no apparent difference in the levels between L186T heterozygous and 186L homozygous donors (Supplementary Fig. 7). Otherwise, sinusoidal endothelial cells and biliary ducts showed moderate positivity. Interestingly however, the sample from one heterozygous patient characterized by the lowest grade of steatosis also showed the weakest staining intensity for PSD3. Regarding ARF6, hepatocytes showed multifocally moderate cytoplasmic and membrane positivity. The staining for activated ARF6, especially in membranes, appeared more pronounced in samples homozygous for 186L (Supplementary Fig. 7). However, immunohistochemistry does not allow for the quantification of PSD3 or activated ARF6.

### *Psd3* downregulation protects against FLD in mice

**Fig. 6:**
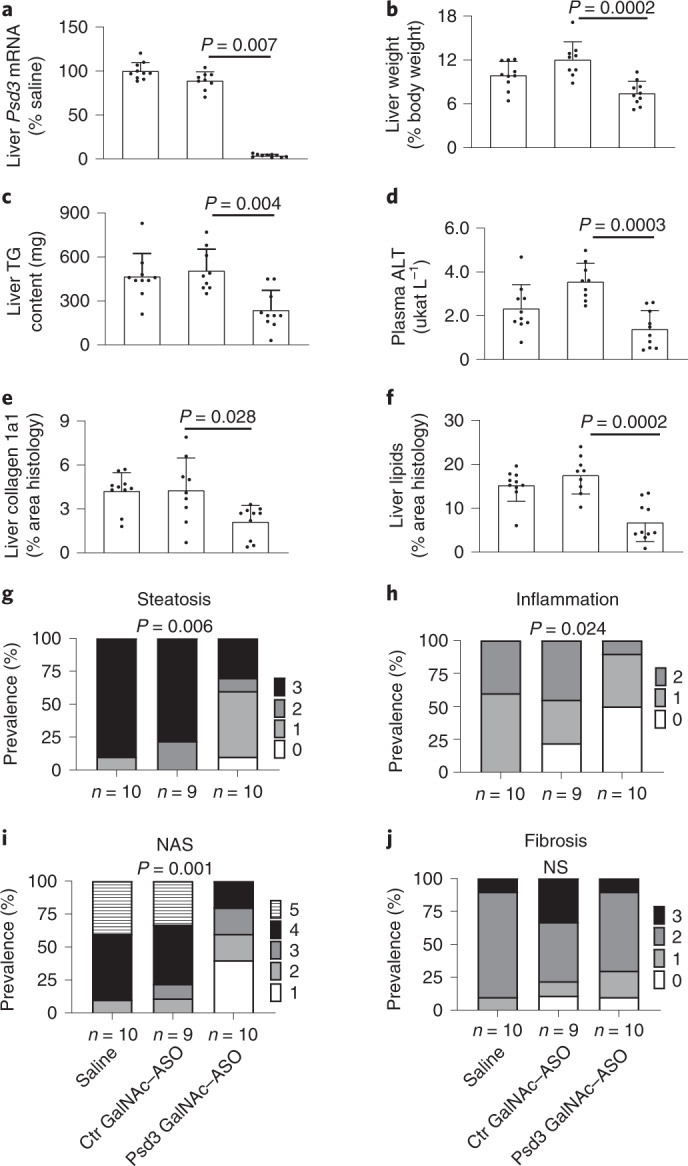
Liver *Psd3* downregulation in mice fed a NASH-inducing diet reduced the severity of steatosis, inflammation and NAS, and liver collagen levels. C57BL/6 male mice were fed a NASH-inducing diet for a total of 50 weeks and during the last 16 weeks, groups of mice were dosed via once weekly subcutaneous injections with saline (*n* = 10 animals), control GalNAc–ASO (*n* = 9 animals, 5 mg per kg body weight per week) or Psd3 GalNAc–ASO (*n* = 10 animals, 5 mg per kg body weight per week). **a**–**d**, Psd3 GalNAc–ASO reduced liver *Psd3* mRNA expression levels (**a**) and was associated with reduced liver weight (**b**), total liver triglyceride content (**c**) and plasma ALT levels (**d**). **e**,**f**, Psd3 GalNAc–ASO treatment reduced liver Col1a1 protein levels (**e**) and liver lipid droplet number (**f**). **g**–**j**, Psd3 GalNAc–ASO treatment reduced the severity of steatosis (**g**), inflammation (**h**) and NAS (**i**), although there were no significant changes in liver fibrosis grade (**j**). **a**–**f**, Data are presented as mean ± s.d. Two-sided *P* values were calculated by one-way ANOVA Kruskal–Wallis non-parametric test followed by Dunn’s correction for multiple comparisons. Multiple comparisons were performed between the mean of each group and the mean of the control group (Ctr GalNAc–ASO). **g**–**j**, Data are presented as prevalence (%) of the specified disease’s degree. Colour shading from white to black indicates increased disease severity. Two-sided *P* values were calculated by using ordinal regression analyses. NS, non-significant. Source data

**Fig. 7:**
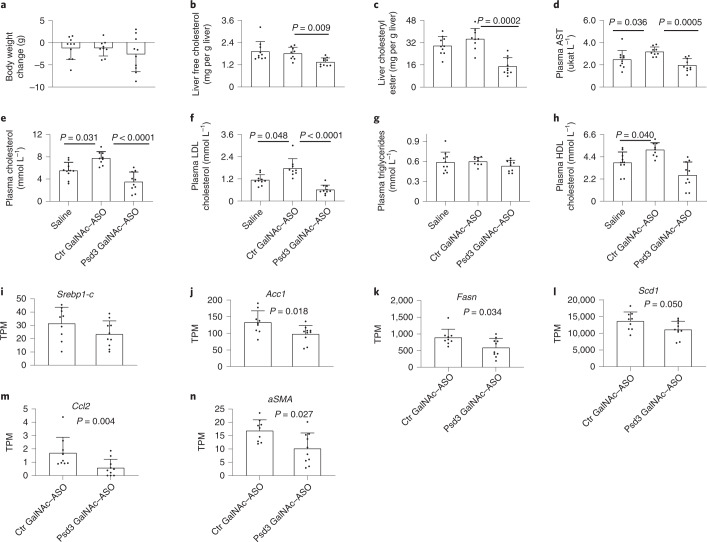
Liver *Psd3* downregulation in mice fed a NASH-inducing diet reduced liver cholesterol and circulating LDL cholesterol levels and reduced expression of hepatic genes involved in de novo lipogenesis. C57BL/6 male mice were fed a NASH-inducing diet for a total of 50 weeks and during the last 16 weeks, groups of mice were dosed via once weekly subcutaneous injections with saline (*n* = 10 animals), control GalNAc–ASO (*n* = 9 animals, 5 mg per kg body weight per week) or Psd3 GalNAc–ASO (*n* = 10 animals, 5 mg per kg body weight per week). **a**–**h**, Psd3 GalNAc–ASO treatment did not affect body weight gain (**a**) but reduced liver cholesterol (**b**) and cholesteryl ester (**c**), plasma AST (**d**), total cholesterol (**e**) and LDL cholesterol levels (**f**). There were no effects on plasma triglyceride (**g**) or HDL cholesterol (**h**) levels. Hepatic mRNA was quantitated by digital gene expression profiling as described in the Methods and expressed as transcripts per million. **i**–**n**, Psd3 GalNAc–ASO treatment had no effect on hepatic Srebp1-c (**i**) but significantly reduced hepatic Acc1 (**j**), Fasn (**k**), Scd1 (**l**), αSma (**m**) and Ccl2 (**n**) mRNA expression levels. Data are presented as mean ± s.d. **a**–**h**, Two-sided *P* values were calculated by one-way ANOVA Kruskal–Wallis non-parametric tests followed by Dunn’s multiple comparisons tests. Multiple comparisons were performed by comparing each group with the control group (Ctr GalNAc–ASO). **i**–**n**, Two-sided *P* values were calculated by Mann–Whitney non-parametric tests. TPM, transcripts per million. Source data

Next, we downregulated liver *Psd3* in vivo by administrating triantennary *N*-acetylgalactosamine (GalNAc)-conjugated Gen 2.5 antisense oligonucleotides (ASOs) in C57BL/6 mice. Mice were fed a non-alcoholic steatohepatitis (NASH)-inducing diet for a total of 50 weeks, and during the last 16 weeks, groups of mice were treated with Psd3 ASO, control ASO or saline. Psd3 ASO treatment decreased the liver *Psd3* mRNA expression levels by 98% (Fig. 6a) and reduced the liver weight, total liver triglyceride content and plasma alanine transaminase (ALT) levels (Fig. 6b–d). Psd3 ASO treatment did not affect the body weight of the mice (Fig. 7a).

**Fig. 8:**
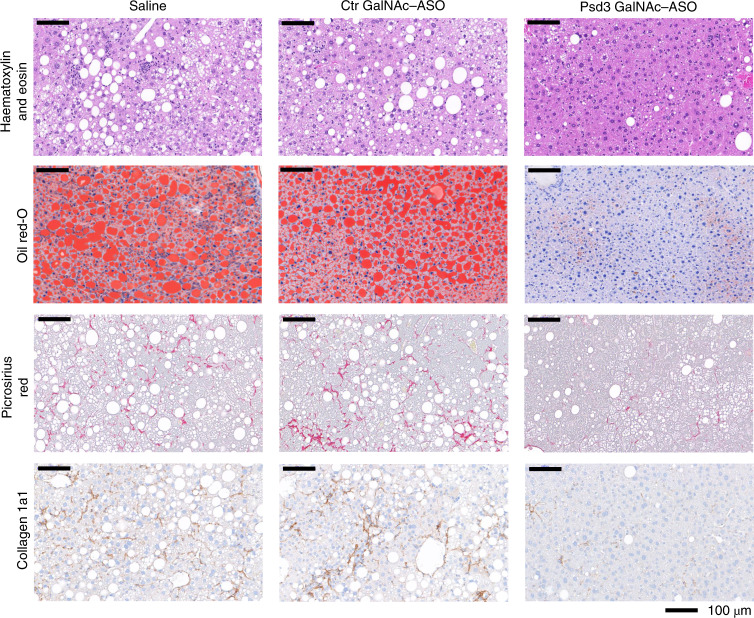
Liver Psd3 downregulation in mice fed a NASH-inducing diet improved liver histopathology. C57BL/6 male mice were fed a NASH-inducing diet for a total of 50 weeks and during the last 16 weeks, groups of mice were dosed via once weekly with subcutaneous injections of saline (*n* = 10 animals), control GalNAc–ASO (*n* = 9 animals, 5 mg per kg body weight per week) or Psd3 GalNAc–ASO (*n* = 10 animals, 5 mg per kg body weight per week). Liver sections were stained with haematoxylin and eosin, ORO or picrosirius red according to standard procedures. Consecutive sections were immunohistochemically stained for Col1a1. Representative pictures are presented. Ctr, control. Source data

Moreover, Psd3 ASO treatment reduced the total liver free cholesterol, liver cholesteryl ester, plasma aspartate transaminase (AST), total and LDL cholesterol levels but did not change the plasma triglyceride or high-density lipoprotein (HDL) cholesterol levels (Fig. 7b–h). Histologically, Psd3 ASO treatment reduced the liver collagen 1a1 (Col1a1) protein and liver lipid droplet levels (Figs. 6e,f and 8). Next, to examine the severity of liver disease using the semiquantitative scoring developed by Kleiner *et al*^28^, we performed ordinal regression analyses and found that Psd3 ASO treatment reduced the severity of steatosis and lobular inflammation scores, and the NAFLD activity score (NAS), although the liver fibrosis score did not change significantly (Fig. 6g–j). To gain insights into the mechanisms by which *Psd3* downregulation results in lower hepatic fat content, we examined expression levels of genes involved in triglyceride synthesis. Consistent with the in vitro experiments, *Psd3* downregulation in mice fed the NASH-inducing diet reduced the expression of genes involved in de novo lipogenesis (*Fasn, Acc1. Scd1)* (Fig. 7i–l). *Psd3* downregulation also reduced hepatic expression levels of *monocyte chemoattractant protein 1* (*Ccl2*), involved in monocyte infiltration and *α-smooth muscle actin* (*Acta2*), a marker of myofibroblast formation (Fig. 7m,n).

To confirm our in vivo data in another model, liver *Psd3* levels were silenced using a GalNAc-conjugated Psd3 ASO in mice fed a choline-deficient high-fat diet (CD-HFD) for 12 weeks (Supplementary Figs. 8 and 9). As expected, the CD-HFD led to a reduction in body weight irrespective of the treatment. However, this reduction was recovered at the end of the treatment. Psd3 ASO treatment resulted in robust downregulation of liver *Psd3* mRNA levels. Consistent with the diet-induced NASH model, Psd3 ASO treatment reduced the liver total amount of triglycerides and free cholesterol in the CD-HFD model (Supplementary Fig. 8). Moreover, Psd3 ASO treatment reduced the severity of liver steatosis (Supplementary Fig. 9). However, there were no differences observed in liver inflammation or Col1A1 levels between the Psd3 and control ASO groups in this model (Supplementary Fig. 8).

## Discussion

To date, single-nucleotide sequence variants in *PNPLA3* (ref. ^14^), *TM6SF2* (ref. ^12^), *GCKR* (ref. ^15^), *MBOAT7* (ref. ^13^), *HSD17B13* (ref. ^29^), *MARC1* (ref. ^30^), *APOE* and *GPAM*^31^ have been shown to be associated with FLD. However, there is still a missing heritability for this disease^16,17^. Here, we describe a genetic locus, not previously linked to hepatic fat content, in *PSD3* involved in the susceptibility to FLD in individuals at risk. Furthermore, downregulation of this gene resulted in protection against FLD in mice fed with a NASH-inducing diet, and in lower intracellular fat in human primary hepatocytes cultured in 2D and 3D.

Based on the fact that circulating triglycerides are a proxy for liver fat content^19–23^, we started by examining the association between common non-synonymous variants influencing circulating triglycerides^18^ and liver fat content in the DHS. Using this approach, we found three loci associated with liver fat content. Two of these were already well-known genetic determinants of FLD, namely the *TM6SF2* and *GCKR*, and the third was a dinucleotide polymorphism (rs71519934) in *PSD3*.

Next, we examined the association between the rs71519934 and liver disease in a cohort of 1,951 individuals at risk for liver disease with an available liver biopsy. In this cohort, we found that the rs71519934 186T allele was associated with a reduction in liver steatosis, inflammation, ballooning and fibrosis. Moreover, the rs71519934 was associated with lower circulating lipoprotein levels. To confirm our data, we examined the rs7003060 (in complete linkage disequilibrium (*D*′ = 1, *r*^2^ = 1) with rs71519934) in European individuals (*n* = 10,970) from the UK Biobank with liver fat content measurement available, and found an association with reduced hepatic fat content in individuals with obesity. To further confirm our data, we examined the contribution of the rs71519934 to liver disease in a cohort of individuals with obesity at risk for liver disease from central Europe with liver biopsy available (*n* = 674). In this cohort we found that the rs71519934 was associated with protection against liver steatosis, inflammation and fibrosis. Meta-analyses of the two studies in which liver biopsy was available confirmed a robust effect of the genetic variant against liver steatosis, fibrosis and ballooning. A strength of the genetic study is the replication of the association in two independent cohorts with liver biopsy available; a limitation is that our validation and replication cohorts comprised only European adults and therefore further studies are warranted to test the contribution of this variant to liver disease in other ethnic groups and in children.

The index SNP (rs1495741) identified in the genome-wide association study on circulating triglycerides is an intergenic variant located between *PSD3* and *NAT2*. We did not find any association of *NAT2* missense gene variants with liver fat content. Moreover, there was no difference in the expression of *NAT2* between individuals with or without FLD. These data suggest that *PSD3* and not *NAT2* is involved in FLD. Recently, the rs1495741 was associated at a genome-wide level with susceptibility to drug-induced liver injury^32^, reinforcing the concept of an involvement of this locus in liver disease.

PSD3, also known as EFA6D, EFA6R and HCA67, is a member of the EFA6 protein family. Knowledge about this protein is limited, but proteins in this family have guanine nucleotide exchange activity for ARF6, a small guanosine triphosphatase that regulates endosomal trafficking and cytoskeleton remodelling^33^. *PSD3* was originally identified by gene expression screening on human hepatocellular carcinoma^34^. However, PSD3 has not previously been shown to be involved in hepatic lipid metabolism.

Next, we examined the *PSD3* genotype effect on intracellular neutral lipid levels in human primary hepatocytes cultured in 2D. Consistent with the genetic association data, hepatocytes from a homozygous 186T donor had lower levels of intracellular lipids than homozygous 186L hepatocytes. PSD3 protein levels were elevated when increased amounts of OA were added to the cell cultures with either genotype. However, at any given OA concentration, hepatocytes homozygous for the 186T allele had lower PSD3 protein levels than 186L hepatocytes. Transcriptomic analyses revealed that carriers of the 186T allele had lower expression of key genes involved in the de novo triglyceride and cholesterol biosynthesis.

To gain insight into the mechanisms underlying the genetic association, we examined the hepatic expression of *PSD3* in individuals with or without FLD. Overall, *PSD3* isoform-a was abundantly expressed in the liver. In addition, and consistent with primary human hepatocyte data, individuals with FLD had higher hepatic *PSD3* mRNA expression levels compared with non-FLD controls. Based on this, we hypothesized that *PSD3* downregulation would result in a reduction in intracellular liver fat content. Consistent with this hypothesis, downregulation of *PSD3* resulted in a reduction in neutral lipids in primary human hepatocytes cultured in 2D and 3D. The downregulation-induced reduction was present in both genotypes, except for 186T in 3D in which only a trend was observed. Similar results were observed in human and rat hepatoma cells.

Radiolabelled tracer experiments in vitro showed that downregulation of *Psd3* resulted in a reduction in triglyceride synthesis with no differences in the utilization of fatty acids via beta oxidation. Consistent with the genetic associations with total and LDL cholesterol levels in humans, Apo B secretion was lower in these cells. This may be because of the primary reduction in lipid content due to the lower intracellular synthesis of triglycerides.

Moreover, in silico analyses of the L186T substitution (Supplementary Table 7) indicated that this amino acid change is tolerated. Taking all this together, we speculate that the L186T substitution in PSD3 induces partial loss of function of the protein, becoming apparent in individuals at risk for FLD.

To confirm our data in an in vivo model, we downregulated *Psd3* in mice fed a NASH-inducing diet. Consistent with the human genetic data, downregulation of *Psd3* resulted in protection against liver steatosis, inflammation and collagen deposition in these mice. We did not see any significant changes in liver fibrosis stage in these mice and this may be due to a lack of power to detect this association in the in vivo study. However, the amount of liver Col1a1 visualized by histological staining was lower after downregulation of *Psd3*. By using Mendelian randomization, we have shown that liver fat content per se is deleterious to the liver^35^ causing inflammation and fibrosis. The reduction in inflammation and collagen content observed in this study may be secondary to the reduction in liver fat content due to *Psd3* downregulation. To confirm our in vivo data, we silenced hepatic *Psd3* in mice fed a CD-HFD. In these mice, *Psd3* downregulation resulted in a lower hepatic triglyceride content. However, no differences were observed in liver Col1a1 deposits. This discrepancy could be because this experimental amino acid-deficiency model is very aggressive and develops liver fibrosis in a very short time frame. In addition, this model comes with a negative energy balance and weight loss rather than obesity and insulin resistance, thus the translational relevance of this model to a human NASH patient could be questioned.

To test whether de novo lipogenesis was perturbed in vivo, we examined expression data for the genes involved in triglyceride synthesis. Consistent with the in vitro data, downregulation of *Psd3* resulted in a reduction in key enzymes involved in triglyceride synthesis. What are the mechanisms linking PSD3 to triglyceride synthesis? Recently, we showed that hepatic Mboat7/LYPAAT1 inactivation results in an increase in hepatic triglyceride synthesis^36^. This increase was due to an excess of triglyceride precursors deriving from a non-canonical pathway related to a high phosphatidylinositol turnover. PSD3 is an activator of ARF6, which in turn, is one of the activators of phospholipase D. This lipase hydrolyses phosphatidylcholine to phosphatidic acid and choline. Phosphatidic acid is a main precursor for triglycerides synthesis in the liver. *PSD3* downregulation resulted in a reduction in ARF6 activation. Taking all this together, we speculate that lower ARF6 activation due to *PSD3* downregulation results in less substrate being available for triglyceride synthesis in the liver.

In summary, *PSD3* rs71519934 was associated with protection against FLD in individuals at risk and *PSD3*/*Psd3* downregulation led to a reduction in hepatocellular fat content in human primary hepatocytes and in human and rodent hepatoma cells. Furthermore, liver *Psd3* silencing resulted in protection against high liver fat, inflammation and collagen content in vivo in mice. Further studies are warranted for further mechanistic understanding and to evaluate the potential of this target to treat FLD.

## Methods

### Selection of gene loci

The study design is shown in Supplementary Fig. 1.

In the discovery phase, 32 genetic tag SNPs previously associated with triglycerides as the main (*n* = 24) or secondary trait (*n* = 8) at a genome-wide significance level were selected^18^ (Supplementary Table 1). All missense and nonsense variants within 50 kb of the selected loci that were on the Illumina HumanExome BeadChip (including the tag SNPs when available) were then tested for their association with liver fat content in 2,736 participants in the DHS. Liver fat content was measured using proton magnetic resonance spectroscopy. When the tag SNP was intergenic, variants in both genes adjacent to the tag SNP were examined.

### Identification of the *PSD3* genetic variant

Among the genetic variants tested, three had a MAF >5% in Europeans from the 1000 Genome Project (dbSNP, Current Build 154, released 21 April 2020) and a nominally significant association with liver fat content in the DHS. Among these variants, *TM6SF2* rs58542926 and *GCKR* rs1260326, two well-known genetic variants associated with the entire spectrum of FLD as well as with circulating triglycerides^15,24^, were associated with increased liver fat content. The other variant, namely the rs71519934 (found as rs7003060 that is in complete linkage disequilibrium (*D*′ = 1, *r*^2^ = 1) in the *PSD3* gene, was associated with lower liver fat content.

In the LBC, the rs71519934 had an overall MAF of 33%. When we stratified the cohort based on the centre of recruitment, we found that the rs71519934 had a higher frequency in individuals from Finland (MAF 48% and 42% in the two subgroups from Finland) than in participants from Italy (MAF 26% and 28% in the two subgroups from Italy; Supplementary Table 8) reflecting reference data (Exome Aggregation Consortium database). The genotype frequencies were in HWE in three-quarters of subcohorts (Supplementary Table 8). The deviation in the Milan subcohort may be because this subcohort is a selected population of individuals at risk for liver disease.

Owing to these differences in the MAF, we included the centre of recruitment as a covariate in subsequent multivariate analyses. To understand the effect of the genetic variant, we examined the histological degree of the different components of liver disease, namely, steatosis (the histological measurement of liver fat content), inflammation, ballooning (an index of cell damage) and fibrosis (the main predictor of mortality related to liver disease).

### Study cohorts

#### Dallas Heart Study

The DHS is a multi-ethnic population-based sample of Dallas County residents in which hepatic triglyceride content was measured with proton magnetic resonance spectroscopy (supplementary material) in a subset of 2,736 individuals^37,38^. For the current study, only individuals with a measurement of hepatic triglyceride content have been included. All analyses were based on cross-sectional data. The study was approved by the institutional review board of University of Texas Southwestern Medical Centre, and all individuals provided written informed consent.

#### Liver Biopsy Cohort

The LBC is a cross-sectional study of individuals of European descent who underwent liver biopsy for suspected NASH or severe obesity, consecutively enrolled in three European centres^13,24^. In addition, a total of 323 individuals from a fourth and independent Finnish centre^39^ have been included (supplementary material)^40–42^. In the current study, only individuals with complete data available for both *PSD3* genotype and liver histology were included (*n* = 1,951). The diagnosis of NASH was based on the presence of steatosis with lobular necro-inflammation and ballooning or fibrosis. Disease activity was assessed according to the NAS; fibrosis was staged according to the recommendations of the FLD clinical research network^28^. Scoring of liver biopsies was performed blind by pathologists unaware of the patient’s genotype and status. The study was approved by the ethics committees of the Fondazione IRCCS Ca’ Granda (Milan), Palermo University Hospital (Palermo), Northern Savo Hospital District in Kuopio (Finland), and the ethics committee of the Hospital District of Helsinki and Uusimaa (Finland).

#### UK Biobank

The UK Biobank is a large cohort study comprising more than 500,000 adults (aged between 40 and 69 years at recruitment) who visited 22 recruitment centres throughout the UK between 2006 and 2010. Both the phenotypic and genotypic data used in the current study were obtained from the UK Biobank under Application Number 37142.

For the current study, we restricted our analysis to a subset of unrelated white British participants from the UK Biobank, after further removal of individuals with more than ten putative third-degree relatives, a mismatch between self-reported and genetically inferred gender, putative sex chromosome aneuploidy, withdrawn consent and those who were identified by the UK Biobank as outliers based on heterozygosity and missingness^43,44^.

We used liver MRI-derived PDFF data (data field 22,436). Briefly, participants were scanned with a Siemens MAGNETOM Aera 1.5‐T MRI scanner using a 6‐minute dual‐echo Dixon Vibe protocol, and a single multi-echo slice was further acquired to analyse the liver PDFF^45,46^.

The UK Biobank study received ethical approval from the National Research Ethics Service Committee North West Multi-Centre Haydock (reference 16/NW/0274)^47,48^.

#### The central European independent replication cohort

In total, 674 obese adult Europeans at risk for FLD who underwent percutaneous or surgical liver biopsy were included (supplementary material). NASH was defined by the NAS. The presence of fibrosis was assessed histologically according to the Kleiner classification^28^. Liver biopsies were read by two experienced histopathologists in a blinded fashion. All patients gave their written informed consent for liver biopsy and genetic testing.

### Genotyping

All participants from the DHS were previously genotyped for the variants using an Illumina Infinium HumanExome BeadChip^12,14^.

Participants from the LBC and from the central European independent replication cohort were genotyped by TaqMan 5′ nuclease assays (Life Technologies). The allelic discrimination probe for *PSD3* rs71519934 was not commercially available. A custom assay for this variant has been designed (supplementary material).

Nine individuals denoted as AC homozygotes (*n* = 3), CT homozygotes (*n* = 3) and AC/CT heterozygous (*n* = 3) by the TaqMan assay were Sanger sequenced with consistent results (Supplementary Fig. 2).

UK Biobank participants were genotyped using two highly similar UK BiLEVE or UK Biobank Axiom arrays (>95% overlap). Genotyped data were then imputed based on the 1000 Genomes Phase 3, UK10K haplotype and Haplotype Reference Consortium reference panels^47^. Genotype data for the rs71519934 dinucleotide change were not available in the UK Biobank; the rs7003060 (identifying the first nucleotide change of the rs71519934) was among directly genotyped variants and has been used instead.

### Gene expression analysis

#### Gene expression in human liver biopsies

For human liver biopsies, mRNA expression of the different *PSD3* isoforms, and of *PSD3* and *NAT2* in FLD versus non-FLD was measured in 77 participants from the Milan subset of the LBC (supplementary material)^35,49^. Informed consent was obtained from each participant. The study protocol was approved by the ethics committee of the Fondazione IRCCS Ca’ Granda, Milan, and conformed to the ethical guidelines of the 1975 Declaration of Helsinki.

#### Gene expression in primary and immortalized cells

For primary and immortalized cells, gene expression was assessed by real-time quantitative polymerase chain reaction (quantitative retro transcription PCR) and analysed using the 2^−^^**ΔΔ**Ct^ method (supplementary material).

#### Gene expression in mice

Liver RNA was purified and subjected to quantitative PCR analysis (supplementary material)^50^.

*Psd3* mRNA was quantitated using the primer probe set Mm01351099_m1 (ThermoFisher Scientific). RNA transcript levels were normalized to total RNA levels using Quant-iT RiboGreen RNA reagent (ThermoFisher Scientific)^51^.

For lipogenic and inflammatory gene expression, total RNA was sequenced on a NextSeq500 sequencing instrument (Illumina) (supplementary material).

### In vitro 2D cell culture and quantification of intracellular fat

Cryopreserved primary human hepatocytes were purchased from BioIVT and LONZA. After genotyping, cells were identified as homozygous for 186L (BioIVT lot number: BGW-M00995 (male, aged 50 years, BMI 20.4)) and homozygous for 186T (LONZA lot number: HUM183001 (female, aged 20 years, BMI 28.9)). Rat hepatoma McArdle (McA-RH7777 cells, homozygotes for 180T that corresponds to 186T in human PSD3 according to the alignment of human NP_056125.3 and rat XP_017455908.1) were from ATCC and human hepatocytes Huh7 (PSD3 L186L) were from the JCRB cell bank.

In basal conditions, primary cells were cultured with media provided by the respective companies, McA-RH7777 cells were cultured in DMEM containing 10% foetal bovine serum (FBS), and Huh7 were cultured in DMEM (low glucose) containing 10% FBS.

In experimental conditions, 24 h after seeding, cells were transiently transfected with 30 nM SCR siRNA (AM4611; ThermoFisher Scientific), human *PSD3* siRNA (mix of s23653, s23654 and s23655 for primary and Huh7 cells;, ThermoFisher Scientific), rat *Psd3* siRNA (mix of s157480, s157481 and s157482 for McA-RH7777 cells; ThermoFisher Scientific) or human *ARF6* siRNA (mix of s1565, s1566 and s1567 for Huh7 cells; ThermoFisher Scientific) by Lipofectamine 3000 transfection reagent (L3000-075; ThermoFisher Scientific) according to the manufacturer’s instructions, and grown in serum-free regular medium supplemented with 10 µM (primary cells), 50 µM (McA-RH7777) or 25 µM (Huh7) OA for 48 h.

Intracellular neutral fat content was visualized by ORO staining and images were acquired using an Axio KS 400 Imaging System and AxioVision v.4.8 software (Zeiss) at ×100 magnification. The ORO-stained area was quantified using Biopix iQ software v.2.3.1 (refs. ^52,53^).

### RNA-seq and differentially expressed genes comparison

Human primary hepatocytes from donors homozygous for the 186T or 186L allele were cultured in 2D in their respective media. Total RNA was extracted and its purity and integrity number were evaluated (supplementary material). Illumina sequencing and subsequent analysis was carried out by Novogene (UK) (supplementary material). Differential expression analysis between two conditions/groups (two biological replicates per condition) was performed using the DESeq2 R package (v.2_1.6.3). The resulting two-sided *P* values were adjusted using the Benjamini and Hochberg’s approach for controlling the false discovery rate (FDR). Genes with an adjusted *P* value <0.05 were assigned as differentially expressed.

### 3D liver spheroid formation and quantification of intracellular fat

Cryopreserved primary human hepatocytes from two different donors (as described above) were used to generate spheroids. Cells were seeded into ultralow attachment 96-well plates (Corning) (supplementary material)^54^ and transfected with negative control SCR siRNA or *PSD3* siRNA as described above. The plates were then centrifuged at 100*g* for 5 min. Once the cells have collected at the bottom, self-aggregation leads to the formation of spheroids. Spheroids were cultured for 7 days (supplementary material) and their ATP-based viability was determined using a CellTiter-Glo Luminescent Cell Viability Assay kit (Promega). Cellular ATP was normalized to spheroid volume. Images of the spheroids were taken using an Axio Vert.A1 inverted microscope (Carl Zeiss AG). For quantification of neutral fat, spheroids were fixed and stained as described previously^55^. The ORO-stained area was normalized to the number of 4,6-diamidino-2-phenylindole-stained nuclei and quantified using Image J (v.1.52h, NIH).

### PSD3 polyclonal antibody generation

Human PSD3 (Uniprot Q9NYI0-1) amino acids D527–T1048 were expressed in Sf21 cells in fusion with an N-terminal 6× His tag (separated by a flexible linker) from the pFastBac1 vector (ThermoFisher Scientific). His–PSD3 was purified under endotoxin-free conditions by immobilized metal affinity chromatography on a nickel column (GE Healthcare) and size-exclusion chromatography on a Superdex200 column (GE Healthcare) in 2× PBS buffer pH 7.4. Antibody generation was carried out by Antibody Applications. Purified His–PSD3 (D527–T1048) was used to immunize three male 22-week-old New Zealand white rabbits, using a standard 90-day protocol. Anti-PSD3 IgG from each animal was affinity purified from final bleed rabbit serum on an NHS-activated Sepharose 4 fast flow agarose column (Cytiva) coupled to His–PSD3. A lead antibody was validated via silencing of endogenously expressed PSD3 in human hepatoma HepaRG cells (Supplementary Fig. 10).

### Immunoblotting

Proteins were separated by SDS–PAGE and transferred to nitrocellulose membranes in accordance with standard procedures. Protein lysates were prepared from cell extracts using M-PER protein extraction reagent (ThermoFisher Scientific) supplemented with 10% protease inhibitor (SIGMAFAST; Sigma-Aldrich). Primary antibodies used were: anti-PSD3 custom antibody (dilution 1:1,000, as described above), rabbit anti-calnexin (dilution 1:1,000; Sigma-Aldrich) and mouse monoclonal anti-Arf6 (dilution 1:1,000, provided with the Arf6 activation kit, Cell Biolabs Inc.). Blots were probed with primary antibodies followed by the appropriate horseradish peroxidase-conjugated secondary antibody (dilution 1:2,000) and developed using ECL substrate (Immobilon Western Chemiluminescent HRP Substrate; Merck Millipore).

### ARF6 activation assay

A gamma adaptin ear-containing ARF binding 3 family protein-binding domain (GGA3 PBD) pull-down assay was performed using an ARF6 activation assay kit (STA-407-6, Cell Biolabs) according to the manufacturer’s protocol. Briefly, Huh7 cells were transfected with either SCR siRNA, *PSD3* siRNA as described above, or with *ARF6* siRNA (mix of s1565, s1566 and s1567, ThermoFisher Scientific) for 48 h (supplementary material). Activated ARF6 was then pulled down using GGA3 PBD agarose beads (supplementary material) and along with the corresponding total cell fraction proteins, was analysed by immunoblotting for ARF6 and calnexin.

### PSD3 and active ARF6 immunohistochemistry

Immunostaining for PSD3 and active ARF6 was performed on formalin-fixed and paraffin-embedded liver specimens from eight patients with NAFLD from the LBC (*n* = 4 heterozygotes (L186T) and *n* = 4 homozygotes for the 186L allele of the *PSD3* gene). The automated Ventana BenchMark ULTRA IHC/ISH Staining Module (Ventana) was used together with the Ultraview 3,3′-diaminobenzidine v.1 detection system (Ventana). Tissue sections (4 µm) were deparaffinized (at 72 °C) and incubated in EZPrep Volume Adjust (Ventana). At intervals, steps slides were washed with a cell conditioning solution, Cell Conditioner 1, Tris–EDTA-based buffer, pH 7.8. For analysis of PSD3, sections were first incubated with ultraviolet inhibitor blocking solution for 4 min at 36 °C, then with the protease 1 for 4 min at 36 °C, and finally with anti-PSD3 primary antibody (29-749, dilution 1:100; ProSci) for 1 h at room temperature. For analysis of ARF6, a heat-induced antigen retrieval protocol set for 56 min at 91 °C was carried out using a Tris–EDTA–boric acid, pH 8.4, buffer (Cell Conditioner 1). Sections were incubated with ultraviolet inhibitor blocking solution for 4 min and then with anti-activated ARF6 primary antibody (26918, dilution 1:200; NewEast Biosciences) for 1 h and 4 min at room temperature. For both analyses, incubation with horseradish peroxidase-linked secondary antibody (8 min) (1706515 for PSD3, dilution 1:100, and 170-6516 for active ARF6, dilution 1:150; both Bio-Rad) was followed by incubation with 3,3′-diaminobenzidine chromogen and substrate (8 min) and copper enhancer (4 min). Counterstain (haematoxylin) was applied for 12 min before 4-min incubation with bluing reagent.

Stained tissue sections were analysed under an optical microscope by a pathologist who was blinded to the *PSD3* genotype.

### De novo triglyceride synthesis

De novo triglyceride synthesis was analysed using ^3^H-glycerol^56^. Briefly, McA-RH7777 and Huh7 cells were seeded in triplicate in six-well plates. Twenty-four hours after seeding, cells were transfected with *Psd3* siRNA or SCR siRNA for 48 h in regular medium (supplementary material). Forty-eight hours after transfection, cells were incubated with ^3^H-glycerol (PerkinElmer) plus OA for 15, 30 or 60 min. Cell lysates were collected and lipids were extracted (supplementary material)^57^, separated and quantified by thin-layer chromatography. Spots corresponding to triglycerides were visualized with iodine vapour and added to vials with scintillation fluid. Radioactivity was measured using a scintillation counter as disintegrations per min.

### Apolipoprotein B secretion

Apo B secretion in vitro was measured by adapting the previously described protocol^53^. Briefly, McA-RH7777 cells were transfected with *Psd3* siRNA or SCR siRNA. Forty-eight hours after transfection, cells were labelled with ^35^S Met/Cys (PerkinElmer) and chased for 5, 15, 30 or 60 min (supplementary material). Apo B was then immunoprecipitated (supplementary material) and subsequently separated on 3–8% gradient SDS–PAGE. The gel was then dried, exposed overnight on BAS-MS film and visualized using Phosphoimager (Fujifilm FLA-3000).

### Beta oxidation

Beta oxidation was analysed using ^3^H-palmitate^58^. Briefly, McA-RH7777 cells were transfected with *Psd3* siRNA or SCR siRNA as described above. Forty-eight hours after transfection, cells were labelled with ^3^H-palmitate (PerkinElmer). Labelled palmitate from the cell media was then precipitated (supplementary material). The supernatant was collected and radioactivity was measured using a scintillation counter as disintegrations per min.

### Liver Psd3 silencing in mice

#### ASO synthesis

Chimeric 16-mer phosphorothioate ASOs containing 2′,4′-constrained 2′-*O*-ethyl at positions 1–3 and 14–16, and a triantennary galactosamine (GalNAc) attached to the 5′-end of the ASOs were synthesized at Ionis Pharmaceuticals, as described previously^59^. Two different Psd3 ASOs were used in the two studies; Psd3 ASO (5′-GTATTAATACTCTCTC-3′) for the diet-induced NASH study, and Psd3 ASO (5′-CTTGATCGAGAATCCT-3′) for the CD-HFD study. The effects were compared with mice dosed with a control ASO targeting no known murine gene (5′-GGCCAATACGCCGTCA-3′). The in vivo studies in mice complied with ethical regulations and were approved by an institutional animal care and use committee, and the Ionis local ethics committee (AAALAC Accreditation #P-0305). All mice were obtained from the Jackson Laboratory and were housed in cages on a 12:12 h light/dark cycle at 22.5 ± 2.5 °C and 50 ± 20% humidity, fed ad libitum and had free access to drinking water for the duration of the studies.

#### Diet-induced NASH and ASO treatment

Six-week-old male C57BL/6 mice (homozygotes for 147T which corresponds to 186T in human PSD3 according to the alignment of human NP_056125.3 and mouse XP_017168192.1) were fed a NASH-inducing diet (D16010101, Research Diets) for 34 weeks. This NASH diet contains 40% of the kilocalories as fat (corn oil), 20% of the kilocalories as fructose and 2% cholesterol, and has previously been shown to accelerate NASH and liver fibrosis progression in mice^60^. Mice were bled and randomized into study groups based on body weight and plasma ALT levels (*n* = 9–10 per group). Mice were maintained on the NASH diet and treated with either saline (*n* = 10 animals), control GalNAc–ASO (*n* = 9 animals, 5 mg per kg body weight per week) or Psd3 GalNAc–ASO (*n* = 10 animals, 5 mg per kg body weight per week) for 16 weeks via weekly subcutaneous injection. During the ASO treatment, body weight was monitored weekly. Seventy-two hours after the final ASO dose, mice were anaesthetized, blood was collected via cardiac puncture, and tissues were collected and either snap frozen in liquid nitrogen or fixed in formalin for histological analyses. Blood was centrifuged at 3,000*g*, and plasma was collected. The plasma and snap-frozen tissues were stored at −80 °C.

#### CD-HFD and ASO treatment

Six-week-old male C57BL/6 mice were administered a weekly subcutaneous injection of either saline, control GalNAc–ASO or a Psd3 GalNAc–ASO at a dose of 5 mg per kg body weight per week for 14 weeks (*n* = 12 per group, 15 injections). For the first two weeks of treatment (three injections), all mice were fed a chow diet (5V12; LabDiet), and thereafter the mice were switched to a choline-deficient l-amino acid defined high-fat diet (A06071302; Research Diets) for the remaining 12 weeks of ASO treatment (12 injections). A smaller group of mice (*n* = 4) were kept on chow diet and dosed with saline for the entire study. During the ASO treatment period, body weight was monitored weekly. Seventy-two hours after the last ASO dose, mice were anesthetized, blood was collected via cardiac puncture and tissues were collected and either snap frozen in liquid nitrogen or fixed in formalin for histology analyses. Blood was spun at 3,000*g* and plasma was collected. Plasma and snap-frozen tissues were stored at −80 °C.

#### Plasma and liver biochemistry

Plasma transaminases (AST, ALT), total plasma cholesterol, plasma triglycerides, LDL and HDL cholesterol were quantitated using an Olympus clinical analyser (Beckman Coulter). Liver triglycerides, free cholesterol and cholesteryl ester were quantitated as described previously^61^.

#### Histopathology and image analysis

Snap-frozen liver samples were cut (6 µm thick) and stained with ORO according to standard procedures. After formalin fixation, dehydration and paraffin embedding, 4-µm sections were stained with haematoxylin and eosin and picrosirius red according to standard procedures. Consecutive sections were immunohistochemically stained for Mac2 (CL8942AP; Cedarlaine) and Col1a1 (LS-C343921; BioSite) in an automated Ventana Ultra system (Ventana Medical Systems). Image analysis was performed on digital images using Visiopharm Integrator System software (v.2018.09). Lipid-filled vacuoles, liver steatosis, inflammation, the NAS and the fibrosis stage were evaluated in the haematoxylin and eosin- and picrosirius red-stained liver sections according to the methods reported by Kleiner et al^28^. All histological assessments were performed blind by a board-certified veterinary pathologist.

### Statistical analysis

For the DHS, the *P* values for associations between liver fat content and the target variants were calculated using linear regression analysis adjusted for age, gender and the four leading principal components of ancestry. An additive genetic model was used in all analyses.

For the LBC and for the central European independent replication cohort, the association between the *PSD3* rs71519934 variant and liver disease was evaluated under an additive genetic model by binary logistic (prevalence of liver disease) or ordinal regression (liver histological features) analysis adjusted for age, gender, BMI, centre of recruitment and number of PNPLA3 I148M mutant alleles. All analyses were performed using IBM SPSS statistics v.27.

For the UK Biobank, liver PDFF was first rank-based inverse normal transformed, and then the association with *PSD3* rs7003060 was examined using linear regression adjusted for age, gender, BMI, the first ten genomic principal components and array type under an additive genetic model using R v.3.6.1, MATLAB R2020b (academic license).

For descriptive statistics, data are shown as the mean and s.d. or the median and interquartile range (i.q.r.) as appropriate. Categorical traits are shown as numbers and proportions. For continuous traits, *P* values were calculated by linear regression under an additive genetic model unadjusted or adjusted for age, gender and BMI. Non-normally distributed traits were log-transformed before being entered into the model. For categorical traits, *P* values were calculated by the chi-squared test or by binary logistic regression adjusted for age, gender and BMI using IBM SPSS statistics v.27.

Differences in *PSD3* and *NAT2* expression levels in human tissues were evaluated by the Mann–Whitney non-parametric test (for comparisons between healthy individuals and those with FLD) or by linear regression analysis (for comparisons among genotypes).

Differential gene expression analysis in primary cells between two conditions/groups (two biological replicates per condition) was performed using the DESeq2 R package (v.2_1.6.3) as described above (‘RNA-seq and differentially expressed gene comparison’). The resulting two-sided *P* values were adjusted using the Benjamini and Hochberg’s approach for controlling the FDR.

For meta-analyses of the histological cohorts, an inverse variance meta-analysis of the two studies (LBC and central European replication cohort) was performed using package ‘meta’ with fixed and random effect models in R v.3.6.1 (ref. ^62^).

For in vivo and in vitro studies, data are shown as the mean and s.d. *P* values were calculated by the Mann–Whitney non-parametric test (in vitro) or one-way analysis of variance (ANOVA) Kruskal–Wallis non-parametric test with Dunn’s correction for multiple comparisons (in vivo) using GraphPad Prism v.9. The severity scores of liver disease in the in vivo mouse studies were analysed by using ordinal regression analyses. All the reported *P* values are two-sided.

#### Reagent or resource

The catalogue numbers and providers of reagents and resources are detailed in supplementary information.

### Ethical approval

Our research complies with the principles outlined in the Declaration of Helsinki. The specific board/committee and institution that approved each protocol are listed in the relevant section. Each subject provided written informed consent.

### Reporting Summary

Further information on research design is available in the Nature Research Reporting Summary linked to this article.

## Supplementary information

Supplementary Information Supplementary Tables 1–8, Figs. 1–10, Methods and unprocessed blots for supplementary figures.

Reporting Summary

Supplementary Data 1 Statistical source data for Supplementary Fig. 3.

Supplementary Data 2 Statistical source data for Supplementary Fig. 4.

Supplementary Data 3 Combined microscopy image for Supplementary Fig. 5 as a PDF.

Supplementary Data 4 Statistical source data for Supplementary Fig. 5.

Supplementary Data 5 Combined microscopy image for Supplementary Fig. 6 as a PDF.

Supplementary Data 6 Statistical source data for Supplementary Fig. 6.

Supplementary Data 7 Statistical source data for Supplementary Fig. 8.

Supplementary Data 8 Microscopy image for Supplementary Fig. 9.

Supplementary table Supplementary table with catalogue numbers of reagents.

Supplementary data Microscopy images for Supplementary Fig. 7.

## Data Availability

All data associated with this study are present in the paper or the Supplementary Information. For UK Biobank, all individual-level phenotype/genotype data are accessible via a formal application to the UK Biobank http://www.ukbiobank.ac.uk. Antisense oligonucleotides associated with this study can be made available, on reasonable request, to academic researchers under a material transfer agreement with AstraZeneca and Ionis Pharmaceuticals. Owing to study participants’ privacy data protection, the RNA-seq data of the liver biopsies of the LBC can only be made available on request to the corresponding authors for collaborative projects. Bulk RNA-seq data of the primary human hepatocytes are deposited in the NCBI SRA under the BioProject identifier PRJNA778044. All other data are available from the authors on reasonable request. The following online databases have been used: Database of Single Nucleotide Polymorphisms (dbSNP) (https://www.ncbi.nlm.nih.gov/snp/); Ensembl (https://www.ensembl.org/index.html); Exome Aggregation Consortium [ExAC] database (http://exac.broadinstitute.org). Source data are provided with this paper.

## Source data

Source Data Fig. 1 Statistical source data.
Source Data Fig. 2 Statistical source data.
Source Data Fig. 3 Unprocessed western blot.
Source Data Fig. 3 Statistical source data.
Source Data Fig. 4 Unprocessed western blot.
Source Data Fig. 4 Statistical source data.
Source Data Fig. 5 Unprocessed western blot.
Source Data Fig. 5 Statistical source data.
Source Data Fig. 6 Statistical source data.
Source Data Fig. 7 Statistical source data.
Source Data Fig. 8 Microscopy image combined.

## Code availability statement

Source data are provided with this paper. All codes and scripts used for association studies are available on request. Codes used for image analysis available on request.
