# Supplementary material for: PSD3 downregulation confers protection against fatty liver disease
Source: Nat Metab. 2022 Jan 31;4(1):60–75. doi: 10.1038/s42255-021-00518-0 (PMC8803605; doi:10.1038/s42255-021-00518-0)
Supplement: Supplementary file 1 — Supplementary Tables 1–8, Figs. 1–10, Methods and unprocessed blots for supplementary figures. [file 42255_2021_518_MOESM1_ESM.pdf]

nature portfolio

https://doi.org/10.1038/s42255-021-00518-0

Supplementary information

PSD3 downregulation confers protection against fatty liver disease

In the format provided by the authors and unedited

Supplementary materials

Supplementary table 1: Thirty-two loci affecting triglycerides in Europeans (*1*).

Locus	Tag SNP	Lead trait	Other traits
ANGPTL3	rs2131925	TG	TC, LDL
GALNT2	rs4846914	HDL	TG
APOB	rs1042034	TG	HDL
GCKR	rs1260326	TG	TC
COBLL1/LOC101929615/GRB14	rs10195252	TG	
IRS1/CUL3	rs2972146	HDL	TG
MSL2L1/PCCB	rs645040	TG	
KLHL8	rs442177	TG	
MAP3K1	rs9686661	TG	
TIMD4	rs6882076	TC	LDL, TG
HLA/WASF5P	rs2247056	TG	
TYW1B	rs13238203	TG	
MLXIPL/TBL2/BCL7B	rs17145738	TG	HDL
PINX1	rs11776767	TG	
NAT2/PSD3	rs1495741	TG	TC
LPL/SLC18A1	rs12678919	TG	HDL
TRIB1/LOC101927634	rs2954029	TG	TC, LDL, HDL
JMJD1C	rs10761731	TG	
CYP26A1/NIP7P1	rs2068888	TG	
FADS1-2-3	rs174546	TG	HDL, TC, LDL
APOA1	rs964184	TG	TC, HDL, LDL
LRP1	rs11613352	TG	HDL
ZNF664	rs4765127	HDL	TG
CAPN3	rs2412710	TG	
FRMD5	rs2929282	TG	
LIPC	rs1532085	HDL	TC, TG
CTF1	rs11649653	TG	
CETP/HERPUD1	rs3764261	HDL	TC, LDL, TG
CILP2/TM6SF2/SUGP1	rs10401969	TC	TG, LDL
APOE/APOC1	rs439401	TG	
PLTP/PCIF1	rs6065906	HDL	TG
PLA2G6	rs5756931	TG	

A total of 32 genetic tag SNPs previously associated with triglycerides as the main (n=24) or secondary trait (n=8) at a genome-wide significance level were selected.

Abbreviations: TG, triglyceride; TC, total cholesterol; LDL, low-density lipoprotein; HDL, high-density lipoprotein.

Supplementary Table 2: Characteristics of DHS participants stratified by PSD3 L186T genotype.

Characteristic	186L	L186T	186T	*P-value*
**All ethnicities**				
N	1907	728	101	
Age, years	45.6 ± 10.2	45.4 ± 10.4	47.5 ± 9.5	0.59
Female, n (%)	1077 (56.48)	403 (55.36)	53 (52.48)	0.67
BMI, kg/m^2	30.2 ± 6.8	30.2 ± 6.6	28.1 ± 6.1	0.49
TC, mg/dL	184.5 ± 40.9	184.2 ± 36.6	182.1 ± 39.1	0.18
LDL, mg/dL	109.7 ± 37.1	109 ± 33.9	108.9 ± 34.1	0.39
HDL, mg/dL	51 ± 14.9	49.8 ± 14.8	50.4 ± 15.4	0.69
TG, mg/dL	96 (68 - 144)	104 (73 - 155)	101 (72 - 140)	0.32
HTGC, %	3.49 (1.97 - 6.78)	3.74 (1.95 - 7.5)	3.01 (1.43 - 7.29)	0.049
**African Americans**				
N	1096	224	8	
Age, years	46.5 ± 10.1	45.8 ± 10.5	49.1 ± 11.7	0.52
Female, n (%)	642 (58.58)	142 (63.39)	3 (37.5)	0.19
BMI, kg/m^2	30.9 ± 7.2	31.9 ± 7.6	33.1 ± 7.9	0.072
TC, mg/dL	182.8 ± 41.2	181.9 ± 38.3	196.4 ± 42	0.78
LDL, mg/dL	109.4 ± 38.4	107.4 ± 36	120.8 ± 47.1	0.66
HDL, mg/dL	53 ± 15.5	53.4 ± 14.8	54.9 ± 29.1	0.41
TG, mg/dL	86 (63 - 121)	84 (66 - 128)	95 (78 - 136)	0.43
HTGC, %	3.2 (1.78 - 5.43)	3.1 (1.7 - 5.08)	5.39 (1.85 - 10.91)	0.57
**European Americans**				
N	457	344	81	
Age, years	46.7 ± 10.5	46.9 ± 10.1	47.3 ± 9.4	0.66
Female, n (%)	236 (51.64)	170 (49.42)	45 (55.56)	0.58
BMI, kg/m^2	28.6 ± 5.8	29.4 ± 6.1	27.7 ± 5.9	0.99
TC, mg/dL	189.9 ± 39.5	186.2 ± 35.7	179.2 ± 38.4	0.012
LDL, mg/dL	112.5 ± 36	110.7 ± 33.9	106.8 ± 32.3	0.20
HDL, mg/dL	50.1 ± 15.1	48.8 ± 15.4	49.6 ± 13.6	0.25
TG, mg/dL	113 (75 - 173)	112 (79 - 164)	101 (70 - 145)	0.11
HTGC, %	3.64 (2.09 - 7.42)	3.83 (2.02 - 7.98)	2.64 (1.28 - 5.51)	0.073
**Hispanics**				
N	308	149	8	

Age, years	41 ± 8.8	41.3 ± 9.5	49.6 ± 9.4	0.16
Female, n (%)	184 (59.74)	86 (57.72)	4 (50)	0.78
BMI, kg/m^2	30.4 ± 6.5	29.8 ± 5.5	28.5 ± 6	0.21
TC, mg/dL	183.9 ± 42.2	183.8 ± 36	189.6 ± 35.9	0.95
LDL, mg/dL	108 ± 34.3	107.8 ± 30.8	109.8 ± 28.1	0.92
HDL, mg/dL	45.7 ± 10.9	47.5 ± 12.3	52.2 ± 16.8	0.054
TG, mg/dL	122 (81 - 183)	119 (82 - 170)	116 (86 - 176)	0.57
HTGC, %	5.28 (2.7 - 12.54)	4.43 (2.36 - 9.92)	8.65 (4.35 - 12.36)	0.32

Data are shown ad mean ± SD, as median and quartile range, or number and percentage as appropriate. P-values were calculated by linear regression adjusted for age, gender and BMI as necessary, and adjusted or stratified for self-reported ethnicity. Non-normally distributed traits were log-transformed before entering the model. All reported p-values are 2-sided with no adjustment for multiple testing. HTGC, hepatic triglyceride content.

Supplementary table 3: Characteristics of the Liver Biopsy Cohort (LBC) and replication cohort from central Europe.

**Liver Biopsy Cohort**	**N**	**1,951**
Anthropometrical traits	Age, years	47±12
	Male gender, n(%)	909(47)
	BMI, Kg/m2	37±9
Metabolic traits	Diabetes presence, n(%)	507(26)
	Total cholesterol, mmol/L	4.90±1.14
	HDL, mmol/L	1.25±0.36
	LDL, mmol/L	2.93±0.99
	Triglycerides, mmol/L	1.35(0.99-1.91)
	ALT, UI/L	38(23-63)
Liver histology	Steatosis presence, n(%)	1551(79)
	Fibrosis presence, n(%)	1074(55)
	Inflammation presence, n(%)	1134(58)
	Ballooning presence*, n(%)	652(36)
Recruitment centre	Kuopio, Finland, n(%)	410(21)
	Helsinki, Finland, n(%)	145(7)
	Palermo, Italy, n(%)	374(19)
	Milan, Italy n(%)	1022(52)
**Central European Cohort**	**N**	**674**
Anthropometrical traits	Age, years	45±12
	Male gender, n(%)	236(35)
	BMI, Kg/m2	46±10
Metabolic traits	Diabetes presence, n(%)	192(31)
	Total cholesterol, mmol/L	-
	HDL, mmol/L	-
	LDL, mmol/L	-
	Triglycerides, mmol/L	-
	ALT, UI/L	29(20-46)
Liver histology	Steatosis presence, n(%)	549(81)
	Fibrosis presence, n(%)	255(38)
	Inflammation presence, n(%)	212(31)
	Ballooning presence, n(%)	196(29)
Recruitment centre	Germany, n(%)	559(83)
	Austria, n(%)	83(12)
	Switzerland, n(%)	32(5)

Continuous traits are shown as mean ± standard deviation (normally distributed traits) or median and quartile range (non-normally distributed traits). Categorical traits are shown as numbers and proportions.

**data available for n=1,805.*

Supplementary table 4: *PSD3* rs71519934 minor allele associates with less severe liver disease in the LBC after adjustment for genetic risk factors.

	P-value	**OR**	**CI**	
**Steatosis severity**	2.2E-06	0.74	0.65	0.84
**Fibrosis severity**	0.002	0.82	0.71	0.93
**Inflammation severity**	2.3E-06	0.73	0.64	0.83
**Ballooning* severity**	0.003	0.79	0.68	0.92

The association was tested by an ordinal regression analysis adjusted for age, gender, BMI, recruitment centre, number of PNPLA3 I148M mutant allele (n=1,950) and further adjusted for other main genetic risk factors (TM6SF2 rs58542926 [E167K] (n=1,943), MBOAT7 rs641738 (n=1938), and GCKR rs1260326 [L446P] (n=1,863)), and for presence of diabetes. Odds Ratio (OR) and confidence interval (CI) were calculated as exponentials of the coefficient estimate and its CI. All reported p-values are 2-sided with no adjustment for multiple testing.

**data available for n= 1,805*

Supplementary table 5: Characteristics of the Liver Biopsy Cohort (LBC) and the replication cohort from Central Europe stratified by *PSD3* rs71519934.

**Liver biopsy cohort**	**186L**	**L186T**	**186T**	***P-value***
N	917	796	238	
Age, years	47±13	47±12	48±13	0.101
Male gender, n(%)	434(47)	376(47)	99(42)	0.218
BMI, Kg/m^2	35.7±9.0	37.1±8.8	38.2±8.9	2.1×10^{-5}
Diabetes presence, n(%)	222(24)	212(27)	73(31)	0.571
Total cholesterol, mmol/L	5.08±1.13	4.78±1.14	4.62±1.09	1.4×10^{-6}
HDL, mmol/L	1.29±0.39	1.22±0.33	1.2±0.33	0.001
LDL, mmol/L	3.05±0.99	2.86±0.98	2.71±0.94	0.001
Triglycerides, mmol/L	1.35(0.99-1.91)	1.36(1.00-1.96)	1.32(0.98-1.85)	0.828
ALT, UI/L	39(23-66)	38(23-61)	37(24-58)	0.173
**Central European cohort**				
N	330	282	62	
Age, years	44±13	46±12	43±12	0.955
Male gender, n(%)	111(34)	102(36)	23(37)	0.469
BMI, Kg/m2	46±9	46±11	45±11	0.837
Diabetes presence, n(%)	89(30)	82(32)	21(36)	0.295
Total cholesterol, mmol/L	-	-	-	-
HDL, mmol/L	-	-	-	-
LDL, mmol/L	-	-	-	-
Triglycerides, mmol/L	-	-	-	-
ALT, UI/L	31(21-46)	28(19-45)	27(20-44)	0.985

Continuous traits are shown as mean ± standard deviation (normally distributed traits) or median and quartile range (non-normally distributed traits). Categorical traits are shown as numbers and proportions.

For continuous traits, P-values were calculated by linear regression under an additive genetic model unadjusted (age) or adjusted for age, gender and recruitment centre (BMI), or by age, gender, BMI and recruitment centre. Non-normally distributed traits were log-transformed before entering the model.

For categorical traits, P-values were calculated by chi-square test (gender) or by binary logistic regression adjusted for age, gender, BMI and recruitment centre (diabetes). All reported p-values are 2-sided with no adjustment for multiple testing.

Supplementary table 6: Meta-analyses of the association between the rs71519934 and liver histological traits in the Liver Biopsy Cohort (LBC) and the replication cohort from Central Europe.

**Steatosis Severity**	P	OR	CI	
LBC	3.3E-07	0.729	0.645	0.823
Central Europe Cohort	0.158	0.850	0.690	1.060
Meta analyses				
Fixed-effect	2.4E-07	0.755	0.678	0.840
Random-effects	1.24E-04	0.763	0.665	0.876

**Fibrosis severity**	P	OR	CI	
LBC	0.001	0.811	0.715	0.921
Central Europe Cohort	0.04	0.770	0.600	0.990
Meta analyses				
Fixed-effect	1.13E-04	0.803	0.718	0.898
Random-effects	1.13E-04	0.803	0.718	0.898

**Inflammation severity**	P	OR	CI	
LBC	1.6E-07	0.711	0.626	0.807
Central Europe Cohort	0.355	0.880	0.680	1.150
Meta analyses				
Fixed-effect	2.81E-07	0.739	0.659	0.829
Random-effects	0.008	0.764	0.627	0.931

**Ballooning severity**	P	OR	CI	
LBC	0.001	0.777	0.669	0.902
Central Europe Cohort	0.048	0.750	0.570	1.000
Meta analyses				
Fixed-effect	1.27E-04	0.771	0.675	0.881
Random-effects	1.27E-04	0.771	0.675	0.881

An inverse variance meta-analysis of two studies was performed using package "meta" with fixed- and random-effect models in R version 3.6.1(2). All reported p-values are 2-sided with no adjustment for multiple testing.

**Steatosis Presence**	P	OR	CI	
LBC	5.90E-06	0.670	0.570	0.800
Central Europe Cohort	0.024	0.690	0.500	0.950
Meta analyses				
Fixed-effect	4.22E-07	0.674	0.579	0.786
Random-effects	4.22E-07	0.674	0.579	0.786

**Fibrosis presence**	P	OR	CI	
LBC	0.006	0.820	0.720	0.940
Central Europe Cohort	0.049	0.770	0.590	1.000
Meta analyses				
Fixed-effect	7.95E-04	0.808	0.714	0.915
Random-effects	7.95E-04	0.808	0.714	0.915

**Inflammation presence**	P	OR	CI	
LBC	9.90E-07	0.700	0.610	0.810
Central Europe Cohort	0.524	0.920	0.700	1.200
Meta analyses				
Fixed-effect	4.53E-06	0.747	0.659	0.846
Random-effects	0.073	0.785	0.603	1.023

**Ballooning presence**	P	OR	CI	
LBC	0.002	0.790	0.680	0.920
Central Europe Cohort	0.047	0.750	0.560	1.000
Meta analyses				
Fixed-effect	2.52E-04	0.781	0.684	0.892
Random-effects	2.52E-04	0.781	0.684	0.892

An inverse variance meta-analysis of two studies was performed using package "meta" with fixed- and random-effect models in R version 3.6.1(2). All reported p-values are 2-sided with no adjustment for multiple testing.

Supplementary table 7 *In silico* prediction of the effect of the rs71519934 substitution on the protein function.

Tool	Prediction (raw score)	Range	Ref.
SIFT	Tolerated (0.79)		(*3*)‡
PolyPhen	Benign (0)		(*4*)‡
Condel	Neutral (0.001)		(*5*)‡
CADD_raw	-0.34*	-6.46 to 18.30	(*6*)‡
PROVEAN	Neutral (-0.16)		(*7*)#
PhD-SNP	Neutral		(*8*)#

**The larger the score the more likely the SNP has damaging effect, ‡Extracted from VEP dbNSFP* database plugin (version 4.0a), # *predictions are for amino acid sequence.*

Supplementary table 8 *PSD3* genotype frequency and distribution in the Liver Biopsy Cohort (LBC) stratified by center of recruitment.

	Genotypes						Frequency comparison			
	**186L**	**L186T**	**186T**	**MAF**	**HWE P**		**Kuopio, Finland**	**Helsinki, Finland**	**Palermo, Italy**	**Milan, Italy**
**Kuopio, Finland**	108(26)	214(52)	88(21)	0.48	NS	**Kuopio, Finland**	-	NS	<0.001	<0.001
**Helsinki, Finland**	45(31)	78(54)	22(15)	0.42	NS	**Helsinki, Finland**	NS	-	<0.001	<0.001
**Palermo, Italy**	210(56)	135(36)	29(8)	0.26	NS	**Palermo, Italy**	<0.001	<0.001	-	NS
**Milan, Italy**	554(54)	369(36)	99(10)	0.28	0.002	**Milan, Italy**	<0.001	<0.001	NS	-

PSD3 genotype frequency is presented as number and percentage. Comparisons between frequencies have been performed by chi-square test.

Abbreviations: 186L: homozygotes for the Leucine allele; L186T: heterozygotes; 186T: homozygotes for the Threonine allele; MAF, minor allele frequency; HWE, Hardy-Weinberg equilibrium.

Supplementary Figure 1: Schematic representation of the study design and workflow

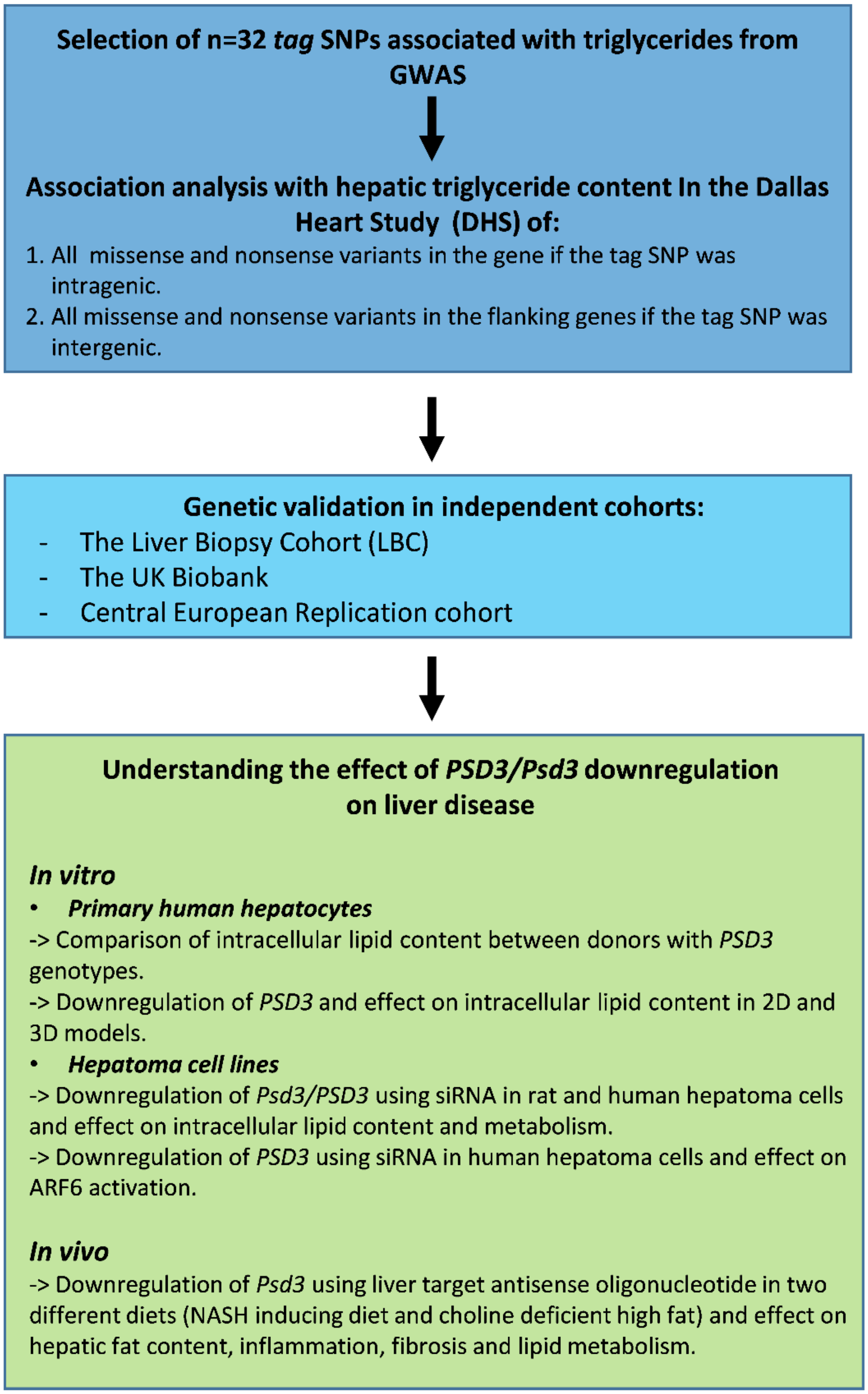

Supplementary figure 2: *Sanger sequencing and allelic discrimination for the PSD3 variant.*

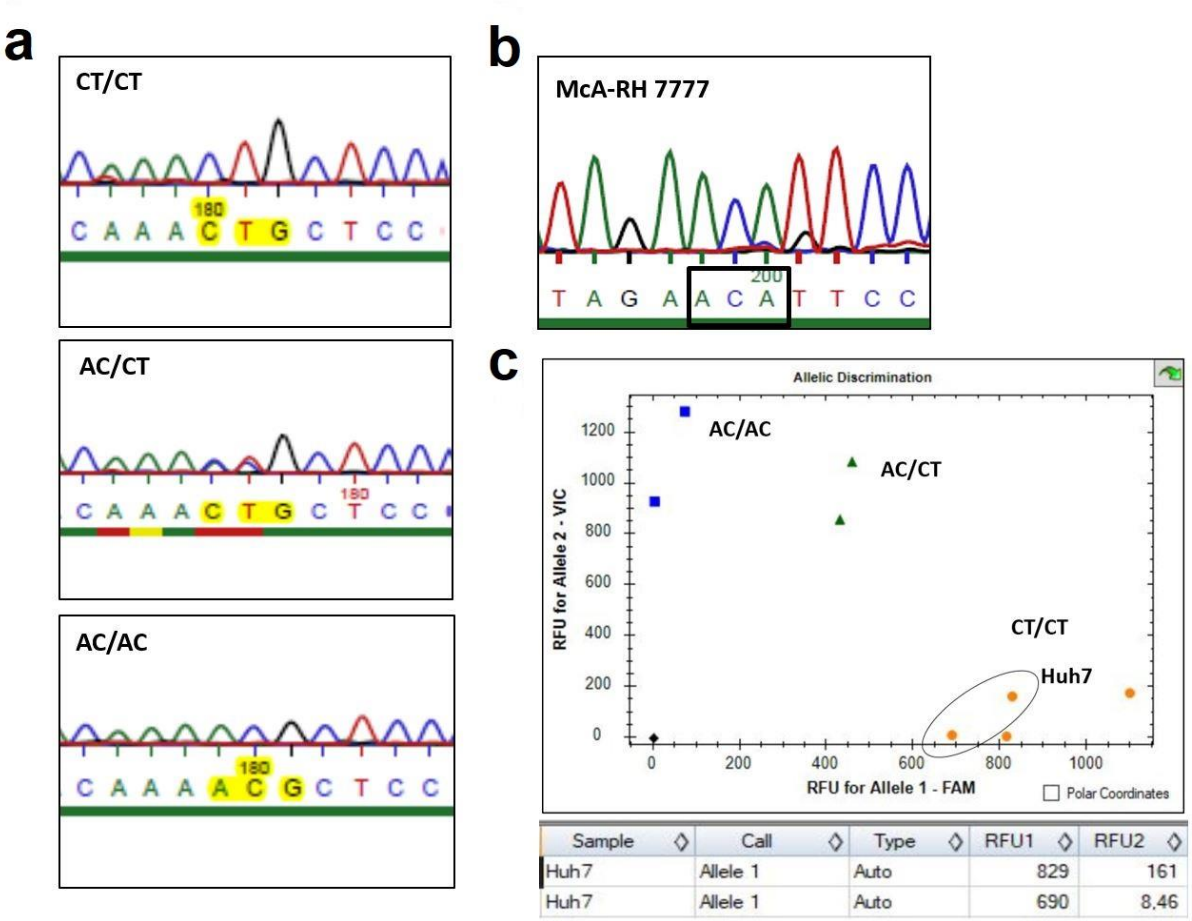

*A total of 9 individuals denoted by the TaqMan assay as AC/AC homozygotes (n=3), CT/CT homozygotes (n=3) and AC/CT heterozygotes (n=3) were Sanger sequenced for cross referencing the genotype and we obtained consistent results. (**a**) Representative electropherograms of each of the groups. (**b**) Representative electropherogram of sanger sequencing of endogenous Psd3 in McA-RH 7777, homozygotes for 180T that corresponds to 186T in human PSD3 according to the alignment of human NP_056125.3 and rat XP_017455908.1*
***(c)** Allelic discrimination plot generated with TaqMan SNP genotyping assay showing Huh7 homozygous for CT (Allele 1).*

Supplementary figure 3: *PSD3* rs71519934 did not interact with *PNPLA3* rs738409 variant on liver disease in LBC.

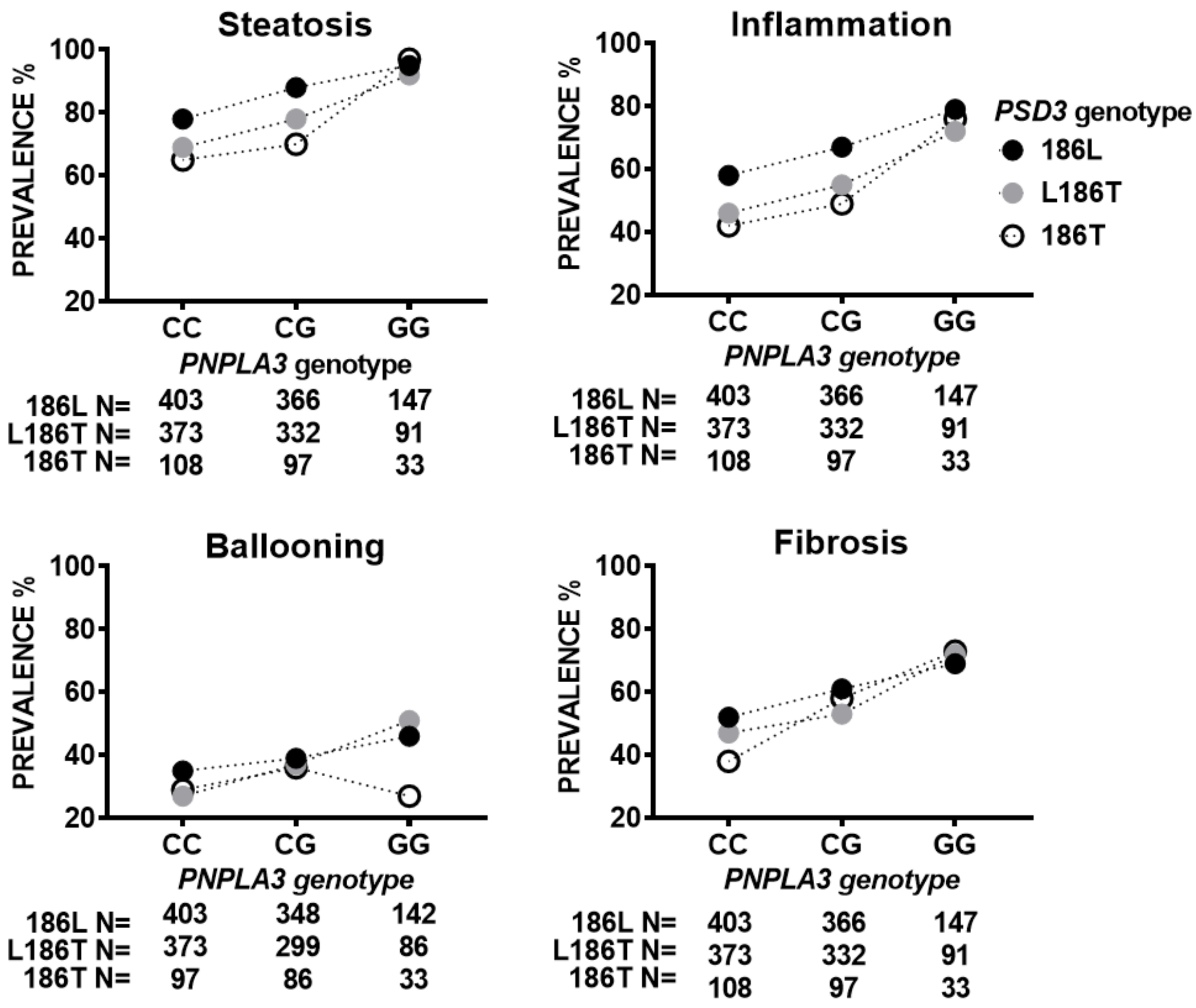

Interaction between PSD3 rs71519934 and PNPLA3 rs738409 variants towards liver steatosis, inflammation, ballooning and fibrosis. Two-sided interactions were tested by including a rs71519934 x rs738409 interaction term in the binary logistic model.

Supplementary figure 4: *Severely obese (BMI>35) carriers of the PSD3 minor allele had lower liver fat content in white British from the UK Biobank.*

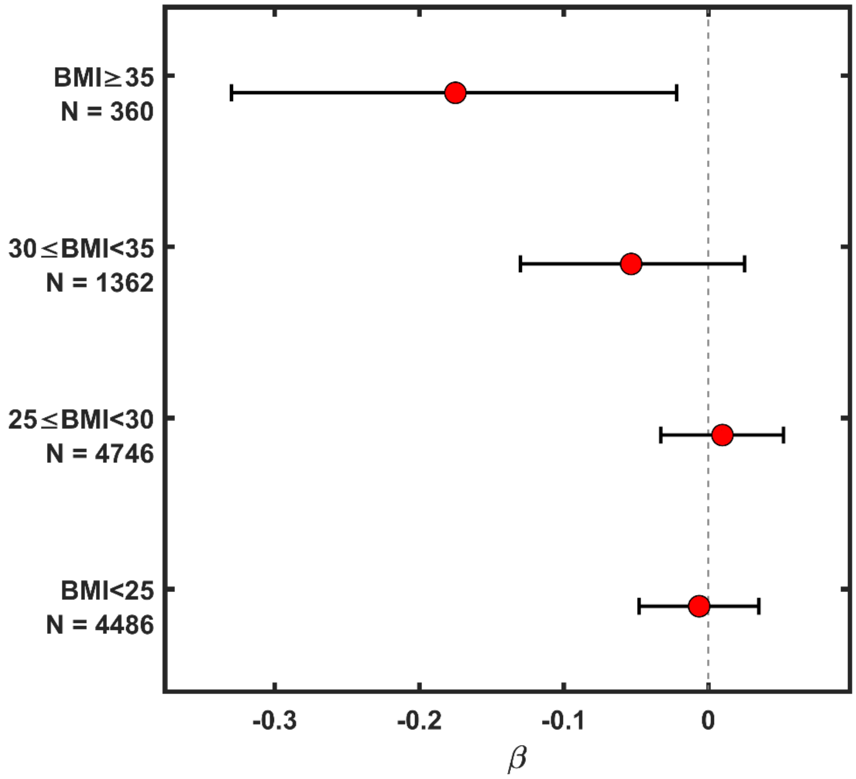

Analysis was performed using a linear regression model adjusted for age, sex, BMI, the first 10 principal components of ancestry and array type. The whiskers show 95% CI.

Supplementary figure 5*: Psd3 downregulation in vitro in hepatoma cells resulted in lower intracellular neutral fat content, de novo triglyceride synthesis and apolipoprotein b (Apo-b) secretion, with no differences in beta-oxidation.*

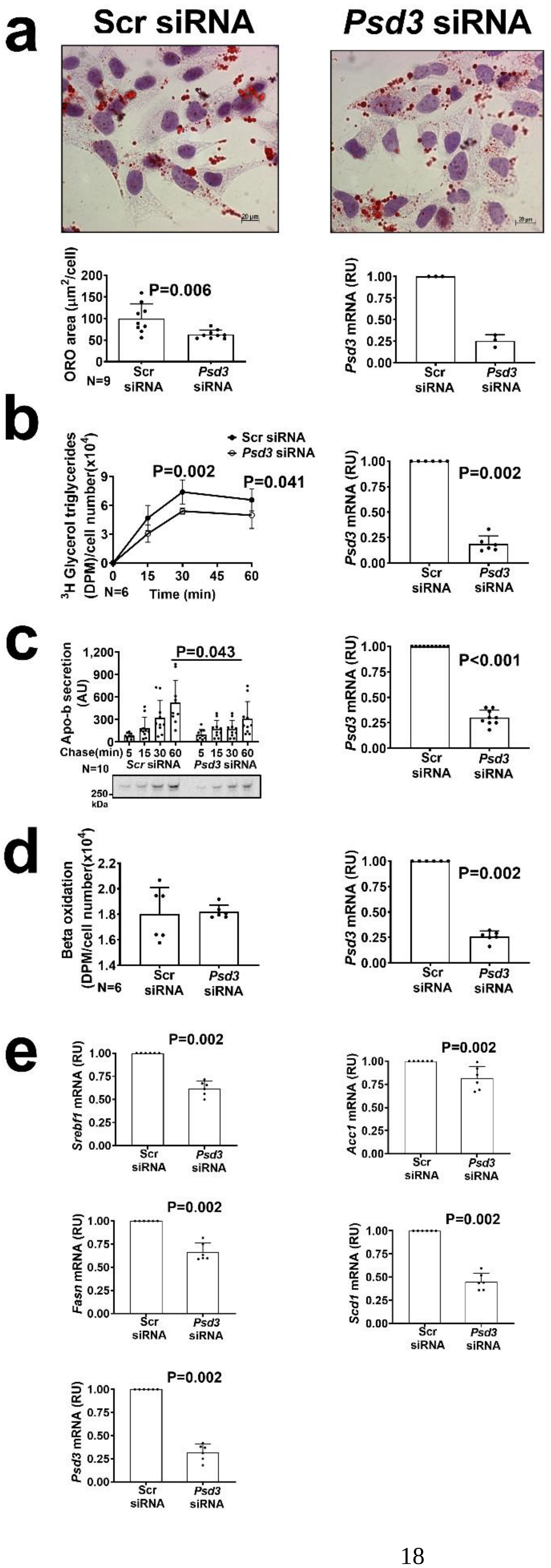
a
Scr siRNA
Psd3 siRNA
20 µm
ORO area (µm²/cell)
P=0.006
N=9
Scr siRNA
Psd3 siRNA
Psd3 mRNA (RU)
b
Scr siRNA
Psd3 siRNA
³H Glycerol triglycerides (DPM)/cell number(x10⁴)
P=0.002
P=0.041
N=6
Time (min)
P=0.002
c
Apo-b secretion (AU)
P=0.043
Chase(min) 5 15 30 60 5 15 30 60
N=10
Scr siRNA
Psd3 siRNA
250-
kDa
P<0.001
d
Beta oxidation (DPM/cell number(x10⁴)
N=6
P=0.002
e
Srebf1 mRNA (RU)
P=0.002
Acc1 mRNA (RU)
P=0.002
Fasn mRNA (RU)
P=0.002
Scd1 mRNA (RU)
P=0.002
Psd3 mRNA (RU)
P=0.002

Downregulation of endogenous Psd3 by using siRNA in McA-RH7777 cells, as compared to cells transfected with scramble (Scr) siRNA. ***(a)*** *intracellular neutral fat content visualized by Oil Red O (ORO) staining (top panels) and quantified by Biopix (bottom left panel);* **(b)** *de novo triglyceride synthesis measured as radiolabelled newly synthesized triglycerides separated by TLC and quantified by scintillation counting 15, 30, 60 minutes after incubation with 5µCi/ml 3H-glycerol plus 50 µM oleic acid;* ***(c)*** *Apo-b secretion as visualized by SDS–PAGE and its quantification. Cells were pulsed with 0.05 mCi/mL 35S Met/Cys + 50 µM OA for 2 hours. Cells were then incubated with chase media with excess L-methionine and L-cysteine for 5, 15, 30 or 60 minutes. Apo-b was immunoprecipitated from media and visualized by phosphorimager after separation on SDS–PAGE.* **(d)** *Beta oxidation measured as precipitated radiolabeled palmitate. Cells were incubated with 8.5 µCi/mL 3H-palmitate +55 µmol/L palmitic acid for 2 hours after which palmitate was precipitated with BSA and perchloric acid and quantified by scintillation counting.*

For each figure panel, the average of Psd3 downregulation efficiency was ~80% as evaluated by real-time quantitative PCR analyzed by the $2^{-\Delta\Delta Ct}$ *method (right panels).*

(*e*) *Expression of genes involved in lipogenesis (N=6).*

For each panel, data shown as mean ± SD of the reported independent experiments. P-values calculated by 2-sided Mann Whitney non parametric test comparing Scr siRNA vs. Psd3 siRNA. Abbr: AU: arbitrary units; RU: relative units; dpm: disintegrations per minute.

Supplementary figure 6: *PSD3* downregulation in human Huh7 hepatoma cells resulted in lower intracellular neutral fat content and lower *de novo* triglyceride synthesis.

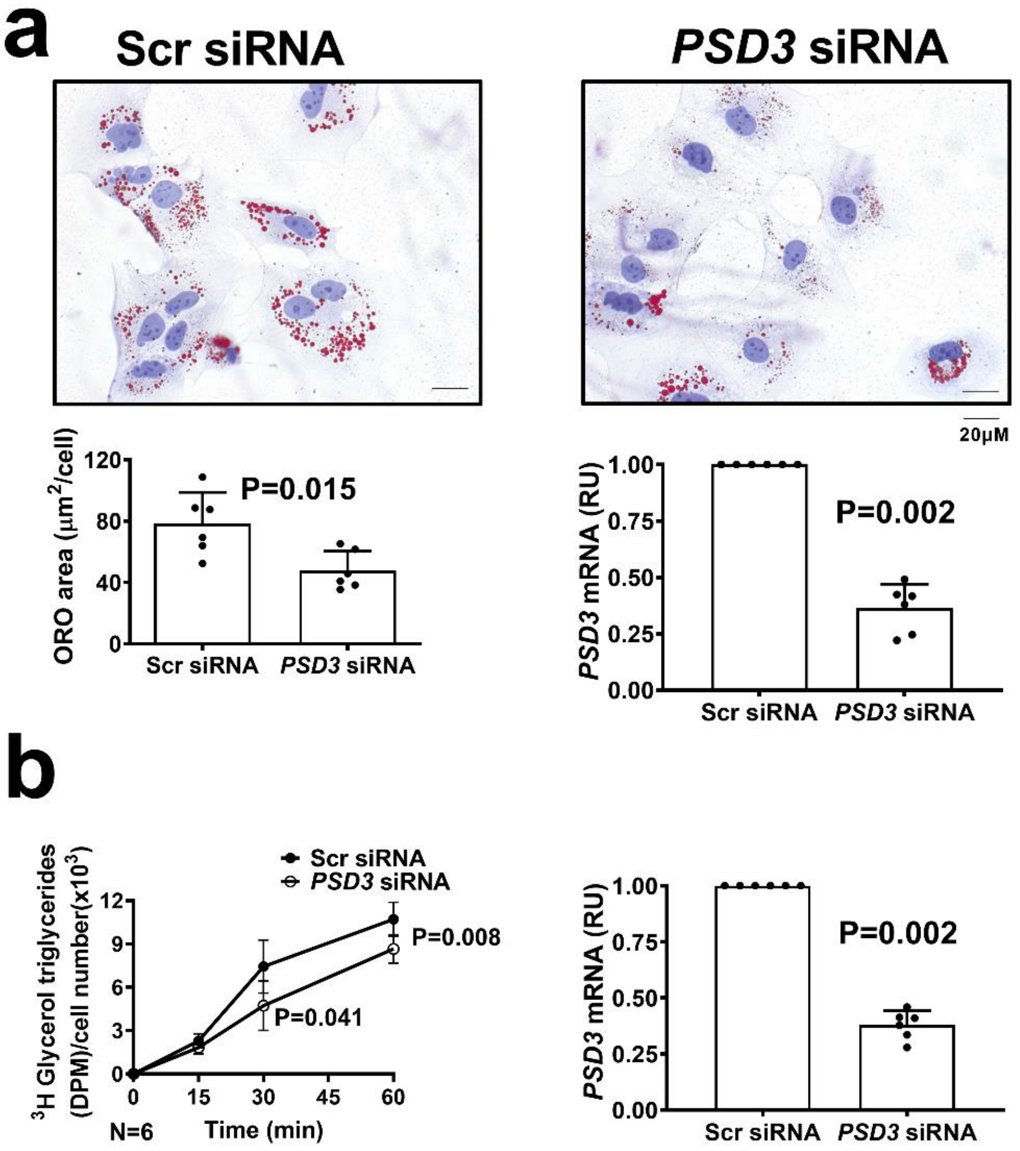

*Downregulation of endogenous PSD3 by siRNA in human Huh7 hepatoma cells. (**a**) intracellular neutral fat content compared to cells transfected with scramble siRNA. Intracellular fat content was visualized by ORO staining (top panels), and the area of ORO was quantified by Biopix. The efficiency of PSD3 mRNA downregulation was ~65%, as evaluated by real-time quantitative PCR analysed by the* $2^{-\Delta\Delta Ct}$ *method. Cells were seeded in triplicate and, 24 h after seeding, they were transfected with scramble or PSD3-siRNA and grown in regular medium without FBS plus 25 µM OA for 48 h. (**b**) de novo triglyceride synthesis as measured by newly synthesized triglycerides separated by TLC and quantified by scintillation*

counting 15, 30, 60 minutes after incubation with 5µCi/ml 3H-glycerol plus 50µM oleic acid. The data are shown as the mean ± SD of the reported independent experiments (N=6). Two-sided P-values calculated by Mann Whitney non parametric test comparing Scr siRNA vs. PSD3 siRNA.

Supplementary figure 7: *PSD3 and active ARF6 staining of individuals with NAFLD heterozygous (L186T) or homozygous (186L) for PSD3.*

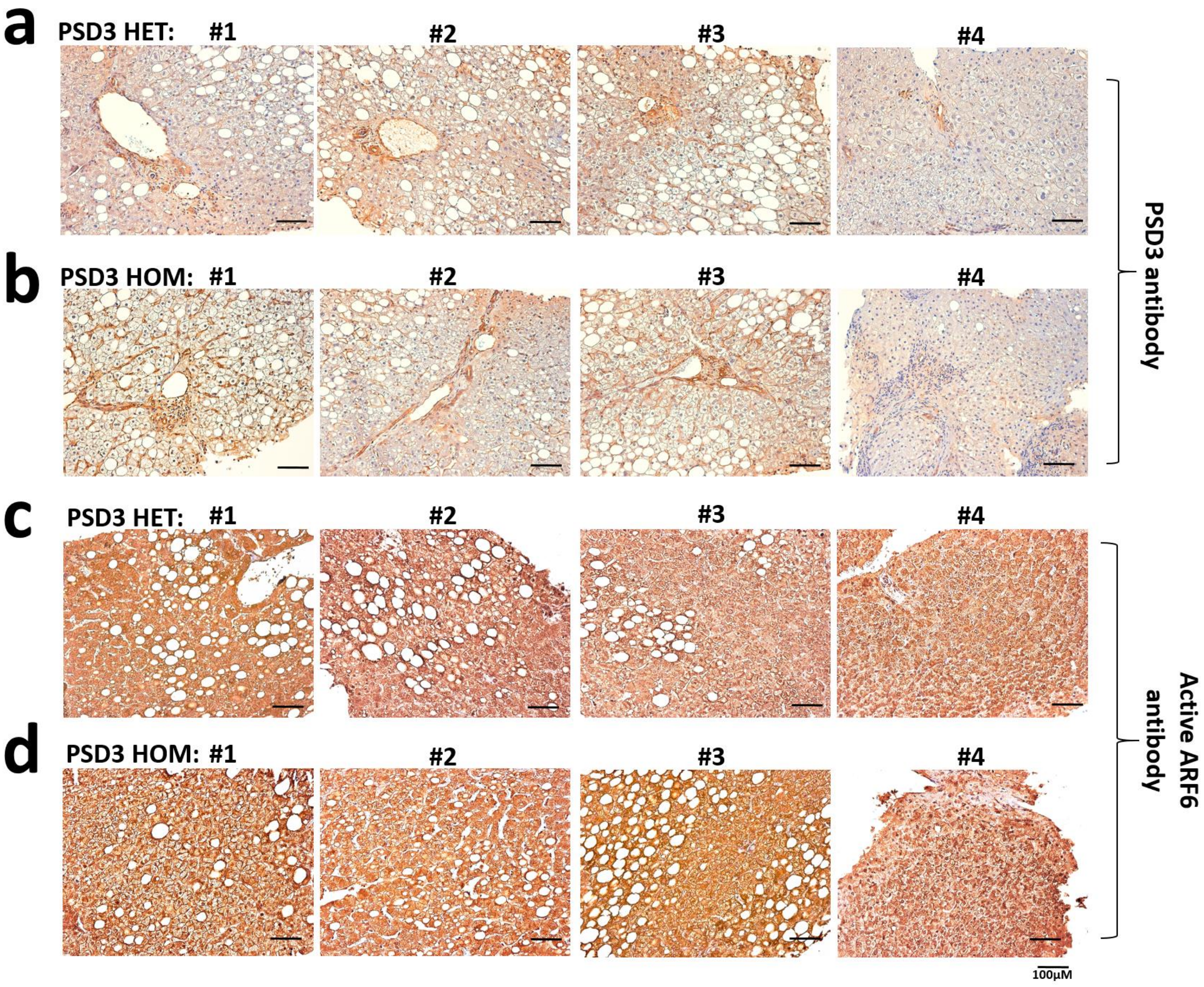

*Immunostaining was performed on formalin-fixed and paraffin embedded liver specimens from eight individuals with NAFLD, n=4 heterozygotes (T186L) and n=4 homozygotes for the (186L) allele of the PSD3 gene. Sections were immunostained with anti-PSD3 and anti-activated ARF6 antibodies according to standard procedures as described in materials and methods. Representative histological slides of PSD3 staining of (**a**) heterozygote (T186L) and (**b**) homozygote (186L). Corresponding active ARF6 staining of (**c**) heterozygote (T186L) and (**d**) homozygote (186L). Experiment performed independently in 4 heterozygote(T186L) and 4 homozygote(T186T) individuals and representative staining images shown for all individuals, as denoted by #1,#2;#3 and #4.*

Supplementary Figure 8: Liver *Psd3* downregulation in mice fed a choline-deficient high fat diet fed mice lowered liver lipid levels.

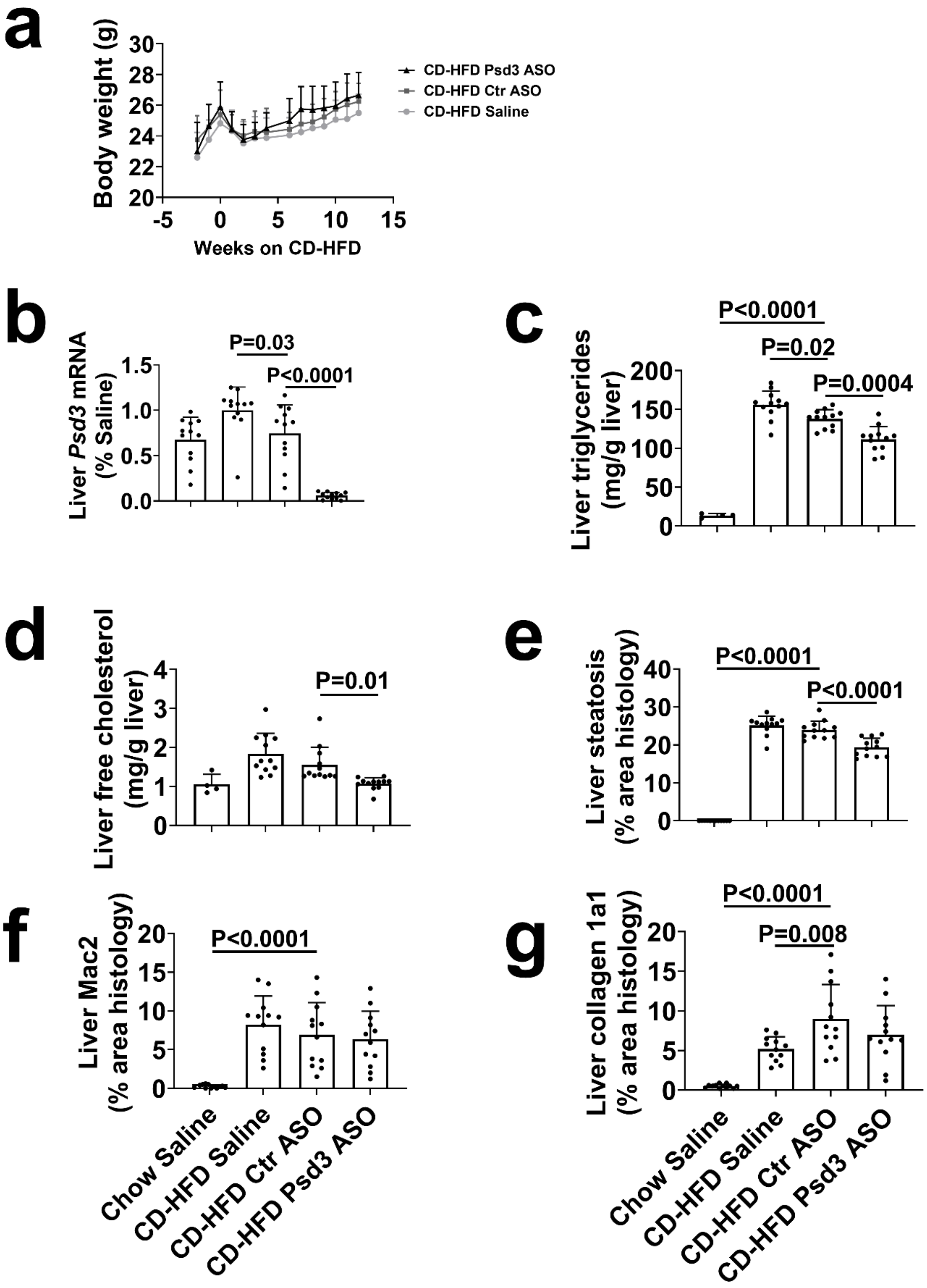
a
Body weight (g)
30
28
26
24
22
20
-5
0
5
10
15
Weeks on CD-HFD
CD-HFD Psd3 ASO
CD-HFD Ctr ASO
CD-HFD Saline
b
Liver Psd3 mRNA (% Saline)
P=0.03
P<0.0001
1.5
1.0
0.5
0.0
c
Liver triglycerides (mg/g liver)
P<0.0001
P=0.02
P=0.0004
200
150
100
50
0
d
Liver free cholesterol (mg/g liver)
P=0.01
4
3
2
1
0
e
Liver steatosis (% area histology)
P<0.0001
P<0.0001
40
30
20
10
0
f
Liver Mac2 (% area histology)
P<0.0001
20
15
10
5
0
Chow Saline
CD-HFD Saline
CD-HFD Ctr ASO
CD-HFD Psd3 ASO
g
Liver collagen 1a1 (% area histology)
P<0.0001
P=0.008
20
15
10
5
0
Chow Saline
CD-HFD Saline
CD-HFD Ctr ASO
CD-HFD Psd3 ASO

C57BL/6 male mice were dosed via once weekly subcutaneous injections with saline (n=12 animals), control GalNac-ASO (n=12 animals, 5 mg/kg/wk), or Psd3 GalNAc-ASO (n=12 animals, 5 mg/kg/wk) for 14 weeks. For the first 2 weeks of treatment mice were fed a chow diet, then switched to a choline-deficient high fat diet (CD-HFD) for the remaining 12 weeks of the study. A smaller group of mice were kept on chow diet and dosed with saline for the entire study (n=4 animals). Psd3 GalNAc-ASO treatment did not significantly impact body weight ***(a)*** *significantly reduced liver Psd3 mRNA expression levels* ***(b)*** *as well as liver triglyceride* ***(c)*** *and liver free cholesterol* ***(d)*** *content. Quantitation of liver histology (supplementary figure 9) showed that Psd3 GalNAc-ASO treatment reduced steatosis* ***(e)*** *but there was no significant impact on liver Mac2* ***(f)*** *or liver collagen 1a1 protein levels* ***(g).*** *The data are presented as the mean values ± SD. P values were calculated by one-way ANOVA Kruskal-Wallis non-parametric test followed by Dunn's correction for multiple comparisons. Multiple comparisons were performed comparing the mean of each group with the mean of the control group (Ctr GalNAc-ASO).*

Supplementary figure 9: *Psd3* downregulation in mice fed choline deficient high fat diet reduced the severity of liver steatosis.

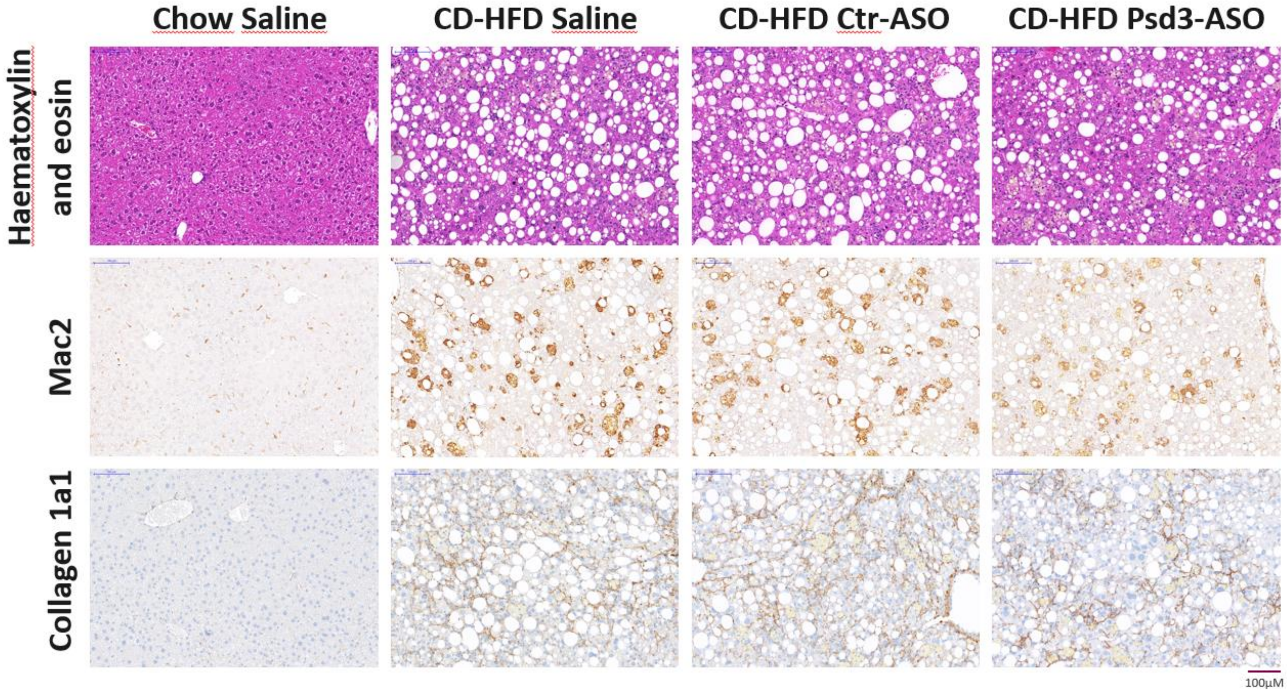

C57BL/6 male mice were dosed via once weekly subcutaneous injections with saline (n=12 animals), control GalNac-ASO (n=12 animals, 5 mg/kg/wk), or Psd3 GalNAc-ASO (n=12 animals, 5 mg/kg/wk) for 14 weeks. For the first 2 weeks of treatment mice were fed a chow diet, then switched to a choline deficient high fat diet (CD-HFD) for the remaining 12 weeks of the study. A smaller group of mice were kept on chow diet and dosed with saline for the entire study (n=4 animals). Liver sections were stained with haematoxylin and eosin (HE), Mac2 or Collagen 1a1 according to standard procedures. Representative pictures are presented.

Supplementary figure 10*:* Custom PSD3 antibody validation *in vitro*.

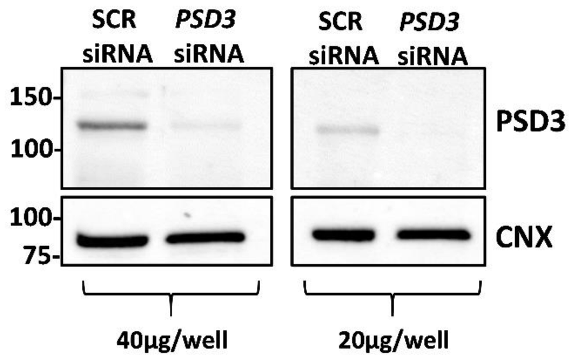

HepaRG cells were grown in regular medium and transfected with 30 nM negative control SCR siRNA or PSD3 siRNA for 48 hours. Membranes were probed with rabbit anti-PSD3 primary antibody (1:1000, details in materials methods) for 1 hour at room temperature. Secondary anti-rabbit HRP conjugated antibody (1:2000) was used and developed using ECL substrate (Immobilon Western Chemiluminescent HRP Substrate, Merck Millipore). Immunoblotting was performed with two different concentrations of cell lysates (40µg/well and 20µg/well, as indicated) to asses sensitivity of the PSD3 antibody. Representative blot shown from experiment performed indenpendently 6 times with similar results.

SUPPLEMENTARY METHODS

Study cohorts

Dallas Heart Study (DHS)

The original cohort was enrolled between 2000 and 2002, and all participants as well as their spouses or significant others were invited for a repeat evaluation in 2007-2009 (DHS-2). For the present study, all analyses were based on cross-sectional data. At the time of enrolment, all participants completed a detailed survey and underwent a clinical examination that involved blood pressure measurement, anthropometry, blood and urine sample collection, and imaging studies. Ethnicity was self-reported. Hepatic triglyceride content was measured with proton magnetic resonance spectroscopy (1H-MRS) in *n*=2,736 participants (*9*). Given the low prevalence of heavy drinking (>30 g/day) in the cohort, we did not exclude subjects based on alcohol intake.

Liver Biopsy Cohort (LBC)

The original LBC cohort includes individuals consecutively enrolled in 3 European centres (*10, 11*). In the present study, to these individuals a total of 323 individuals from a fourth and independent Finnish centre has been included. More specifically, the present study includes 1,951 individuals from: the Metabolic Liver Diseases outpatient service and the Fondazione IRCCS Ca' Granda Ospedale Policlinico Milano, Milan, Italy (n=1,022 (52%)) (*12*); the Gastrointestinal & Liver Unit of the Palermo University Hospital, Palermo, Italy (n=374 (19%)) (*13*); the Northern Savo Hospital District, Kuopio, Finland (n=410 (21%)) (*14*); and the Hospital District of Helsinki and Uusimaa, Finland (n=145 (7%)) (*15*). Individuals with high alcohol intake (men, >30 g/day; women, >20 g/day), viral and autoimmune hepatitis or other causes of liver disease were excluded.

The central European independent replication cohort

A total of 674 adult Europeans with BMI > 30 kg/m^2 who underwent percutaneous or surgical liver biopsy were recruited from tertiary referral centers in: Austria (*n*=83), Germany (*n*=559)

and Switzerland (*n*=32). In all patients infectious (e.g. viral hepatitis, HIV), immunological, drug-induced hepatic steatosis (e.g. amiodarone, methotrexate, steroids, valproate, etc.) or hereditary causes (hereditary hemochromatosis, Wilson disease) of chronic liver disease were excluded by accepted measures. As assessed by self-reporting, subjects with average alcohol consumption of more than 30 g/day (in men) or 20 g/day (in women) were not included.

Design of custom genotyping assay for PSD3 rs71519934

The allelic discrimination probe for *PSD3* rs71519934 was not commercially available. A custom assay for this variant has been designed as follows:

context sequence:
CGTTGTTACTTCAGCTGAAAGAGGTATTTCNGGTAAATTTTTTTGGCCAGCAGGGAG
C[GT/AG]TTTGTTGACTCTCTGTGTTTTACNNCTGGCAGTGTCCANCTCTTTTTCCACC
TGCTGANCTGAAAAACTAGAAACAGCATCTTGGTCCA;

- forward primer: CGTTGTTACTTCAGCTGAAAGAGGTA;
- reverse primer: TGGACCAAGATGCTGTTTCTAGTTT;
- Reporter 1 (VIC) Sequence: TCAACAAAACGCTCCC (reverse complement);
- Reporter 2 (FAM) Sequence: TCAACAAACTGCTCCC (reverse complement).

Gene expression analysis

Gene expression in human liver biopsies

For human liver biopsies, RNA sequencing was performed using the Illumina HiSeq 4000 platform (Novogene, Hong Kong, China). RNA reads were mapped against the human genome, and the gene read count (Ensembl human transcript reference assembly, version 75) was determined using RSEM software version 1.3.0. To quantify gene expression, the RSEM per gene count data were normalized using the DESeq2 package.

Gene expression in primary and immortalized cells

For primary and immortalized cells, RNA was extracted with the RNeasy Plus mini kit (Qiagen) and retro-transcribed using the high-capacity cDNA reverse transcription kit (Thermo Fisher Scientific) according to the manufacturer's instructions. Gene expression was then assessed by real-time qPCR using TaqMan probes (Hs00938064_m1(*PSD3* human) and Rn01402859_g1(*PSD3* rat), Hs01922781_g1 (*ARF6* human) Thermo Fisher Scientific) and master mix (Life Technologies) according to the manufacturer's protocol. All reactions were performed in triplicates. Data were analysed using the $2^{-\Delta\Delta Ct}$ method.

Gene expression in mice

Liver RNA was purified using the RNeasy kit (Qiagen, Hilden, Germany) and subjected to quantitative PCR analysis. The Applied Biosystems StepOne Plus RT-PCR system, which uses real-time fluorescence RT-PCR detection (Thermo Fisher Scientific, Waltham, MA), was used to quantify RNA expression as described previously (*16*).

For lipogenic and inflammatory gene expression, total RNA was isolated from mouse livers as described above. Then, 100 ng total RNA for each sample was used to generate 3'-end RNAseq libraries using the Quantseq Kit 3'mRNA kit (Lexogen, Vienna, Austria). These libraries were pooled and sequenced on a NextSeq500 sequencing instrument (Illumina, San Diego, CA) to a read length of 50 bp and depth of 3-5 million reads per sample. Reads were mapped to gene models using Salmon (ver 0.7.1) using quasi-mapping based quantitation mode and automated libtype detection (*17*). Salmon (ver 0.7.1) provides fast and bias-aware quantification of transcript expression and gene abundance were reported as transcripts per million (TPM) by normalizing gene-associated reads by total mapped reads per sample.

RNA-Seq and differentially expressed genes (DEG) comparison.

Total RNA from human primary hepatocytes homozygous for the 186T or the 186L allele was extracted with the RNeasy Plus mini kit (Qiagen). Its purity and integrity number was evaluated by Agilent Bioanalyzer 2100 system (Agilent Technologies, CA, USA). Then, RNA Illumina sequencing was performed by Novogene (UK) Company Ltd. (Cambridge, UK). Reference

genome and gene model annotation files (Homo sapiens GRCh38/hg38) were downloaded from a genome website browser (NCBI/UCSC/Ensembl) directly. Indexes of the reference genome was built using STAR and paired-end clean reads were aligned to the reference genome using STAR (v2.5). Read numbers of each gene mapped were counted by HTSeq v0.6.1. Next, according to the gene length and read counts mapped to a gene, the FPKM was calculated. Differential expression analysis between the two /groups was performed using the DESeq2 R package (2_1.6.3).

3D liver spheroid formation

To generate spheroids, primary cells were seeded into ultra-low attachment 96-well plates (Corning) at 2000 viable cells per well using 100 µL of serum-containing complete medium. To generate spheroids with primary hepatocytes carrying 186T (donor HUM183001), 5 nM of Z-VAD-FMK was added to the seeding medium to support spheroid formation (*18*). Primary hepatocytes carrying 186L (donor BGW-M00995) was able to form spheroids even without addition of Z-VAD-FMK. Seeded cells were then transfected with 30 nM SCR siRNA (AM4611, Thermo Fisher Scientific), or *PSD3* siRNA (mix of s23653, s23654 and s23655, Thermo Fisher Scientific) by Lipofectamine 3000 transfection reagent (L3000-075 Thermo Fisher Scientific) according to manufacturer's instruction. Day 1 after seeding 100 µL of serum-free maintanence medium was added to achieve a total volume of 200 µL per well. Every 48 hours, 50% of this media was replenished for fresh serum-free medium until day 7.

ARF6 activation assay

The active form of ARF6 has been isolated by pulldown assay using an ARF6 activation assay kit (STA-407-6, Cell Biolabs, Inc.). Huh7 cells were transfected with either scramble SCR siRNA (AM4611, Thermo Fisher Scientific), *PSD3* siRNA (mix of s23653, s23654 and s23655, Thermo Fisher Scientific) or *ARF6* siRNA (mix of s1565, s1566 and s1567, Thermo Fisher Scientific) using Lipofectamine 3000 transfection reagent (L3000-075 Thermo Fisher Scientific). Forty eight hours after transfection, cells were lysed using lysis buffer plus forty microliters of GGA3 PBD agarose beads per 1 mL of each of the cell lysates. The tubes were then incubated at 4°C for 1 hour with gentle agitation. The beads were then pelleted and washed thoroughly 3

times with assay washing buffer to remove traces of unbound proteins. The samples were then eluted by boiling in SDS-PAGE loading buffer. The GGA3 PBD-eluted proteins, along with their corresponding total cell fraction proteins, were subsequently analysed by immunoblotting for ARF6 and Calnexin.

De novo triglyceride synthesis

Twenty-four hours after seeding, McA and HuH7 were transfected with *Psd3* siRNA or scramble (Scr) siRNA for 48 h in DMEM with 10% FBS. Forty-eight hours after transfection, cells were incubated with DMEM with no FBS + 5µCi/ml ^{3}H-glycerol (Perkin Elmer, MA, USA) + 50µM oleic acid (or 25µM oleic acid for Huh7) for 15, 30 or 60 minutes. Cell lysates were collected and lipids were extracted using chloroform methanol (2:1) by the Folch extraction procedure. Briefly, 3mL of 2:1 chloroform:methanol (v/v) (Sigma-Aldrich) and 1mL of acidified solution (17mM NaCl, 1mM H2 SO4) (Sigma-Aldrich) was added to 1mL of cell lysate in PBS (one well of 6 well plate). Samples were centrifuged at 15,000g for 10 minutes, the organic phase was collected and dried under nitrogen gas. Lipids were reconstituted by resolubilizing in 50µl chloroform and separated on TLC silica plates (Merck-Millipore). Triolein (Sigma-Aldrich) was used a marker for triglycerides and Petroleum ether: diethyl ether: acetic acid (40:60:1, v/v) was used a mobile phase. The spots corresponding to triglycerides were visualized with iodine vapor and were cut and added to vials with scintillation fluid (Perkin Elmer). The newly synthesized triglycerides were measured with a scintillation counter (Beckman Coulter LS6500, CA, USA) as disintegrations per minute (DPM).

Apolipoprotein b secretion

McA-RH7777 cells were grown in T-25 flasks in DMEM with 10% FBS and transfected with *Psd3* siRNA or scramble (Scr) siRNA for 48 h in DMEM with 10% FBS. Forty-eight hours after transfection, cells were incubated with DMEM with no methionine and cysteine for 2 hours followed by treatment with 0.05mCi/mL ^{35}S Met/Cys (Perkin Elmer, MA, USA) + 50µM OA for 2 more hours. Then, cells were incubated with chase media, composed of DMEM with surplus of cold L-methionine and L-cysteine (final concentration 10mM, Sigma Aldrich, MO, USA), for 5, 15, 30 or 60 minutes after which media and lysates were collected. Apo-b was then

immunoprecipitated using agarose beads coated with rabbit anti-human Apo-b (Q0497, Dako, Denmark). The samples were eluted from beads by boiling for 5 minutes in 60 µl SDS PAGE sample buffer containing beta mercaptoethanol, and subsequently separated on 3-8% gradient SDS-PAGE. The gel was then dried and visualized using phosphoimager (Fujifilm FLA-3000, Tokyo, Japan).

Beta oxidation

McA-RH7777 cells were grown in triplicates in 6 well plates and transfected with *Psd3* siRNA or Scramble siRNA in DMEM with 10% FBS. Forty-eight hours after transfection, cells were incubated for 2h with 8.5µCi ^{3}H-palmitate plus 55 µmol/L palmitic acid in DMEM with no FBS. Then, 500µl of media was collected and the labelled palmitate was precipitated by adding 50µl of 20% BSA and 27µl of 70% perchloric acid. The supernatant was collected after centrifuging at 12,000 rpm for five minutes and a second aliquot of 20% BSA was added. This was repeated for a total of three times. Then, the final supernatant was added to vials with scintillation fluid. Radioactivity was measured by scintillation counter as disintegrations per min (DPM).

References:

1. T. M. Teslovich *et al.*, Biological, clinical and population relevance of 95 loci for blood lipids. *Nature* **466**, 707–713 (2010).
2. S. Balduzzi, G. Rücker, G. Schwarzer, How to perform a meta-analysis with R: a practical tutorial. *Evid Based Ment Health* **22**, 153-160 (2019).
3. P. C. Ng, S. Henikoff, Predicting deleterious amino acid substitutions. *Genome Res* **11**, 863-874 (2001).
4. I. A. Adzhubei *et al.*, A method and server for predicting damaging missense mutations. *Nat Methods* **7**, 248-249 (2010).
5. A. González-Pérez, N. López-Bigas, Improving the assessment of the outcome of nonsynonymous SNVs with a consensus deleteriousness score, Condel. *Am J Hum Genet* **88**, 440-449 (2011).
6. M. Kircher *et al.*, A general framework for estimating the relative pathogenicity of human genetic variants. *Nat Genet* **46**, 310-315 (2014).
7. Y. Choi, G. E. Sims, S. Murphy, J. R. Miller, A. P. Chan, Predicting the functional effect of amino acid substitutions and indels. *PLoS One* **7**, e46688 (2012).
8. E. Capriotti, R. Calabrese, R. Casadio, Predicting the insurgence of human genetic diseases associated to single point protein mutations with support vector machines and evolutionary information. *Bioinformatics* **22**, 2729-2734 (2006).
9. L. S. Szczepaniak *et al.*, Measurement of intracellular triglyceride stores by H spectroscopy: validation *in vivo*. *Am. J. Physiol.* **276**, E977–E989 (1999).
10. P. Dongiovanni *et al.*, Transmembrane 6 superfamily member 2 gene variant disentangles nonalcoholic steatohepatitis from cardiovascular disease. *Hepatology* **61**, 506–514 (2015).
11. R. M. Mancina *et al.*, The MBOAT7-TMC4 variant rs641738 increases risk of nonalcoholic fatty liver disease in individuals of European descent. *Gastroenterology* **150**, 1219–1230 (2016).
12. L. Valenti *et al.*, The APOC3 T-455C and C-482T promoter region polymorphisms are not associated with the severity of liver damage independently of PNPLA3 I148M genotype in patients with nonalcoholic fatty liver. *J. Hepatol.* **55**, 1409–1414 (2011).
13. S. Petta *et al.*, Glucokinase regulatory protein gene polymorphism affects liver fibrosis in non-alcoholic fatty liver disease. *PLoS One* **9**, e87523 (2014).
14. M. Simonen *et al.*, Desmosterol in human nonalcoholic steatohepatitis. *Hepatology* **58**, 976–982 (2013).
15. P. K. Luukkonen *et al.*, Hepatic ceramides dissociate steatosis and insulin resistance in patients with non-alcoholic fatty liver disease. *J Hepatol* **64**, 1167-1175 (2016).
16. M. J. Graham *et al.*, Antisense oligonucleotide inhibition of apolipoprotein C-III reduces plasma triglycerides in rodents, nonhuman primates, and humans. *Circ. Res.* **112**, 1479–1490 (2013).
17. R. Patro, G. Duggal, M. I. Love, R. A. Irizarry, C. Kingsford, Salmon provides fast and bias-aware quantification of transcript expression. *Nat Methods* **14**, 417-419 (2017).
18. M. Ölander *et al.*, A simple approach for restoration of differentiation and function in cryopreserved human hepatocytes. *Arch Toxicol* **93**, 819-829 (2019).

Unprocessed blots

Supplementary figure 5c: Unprocessed phosphorimager file

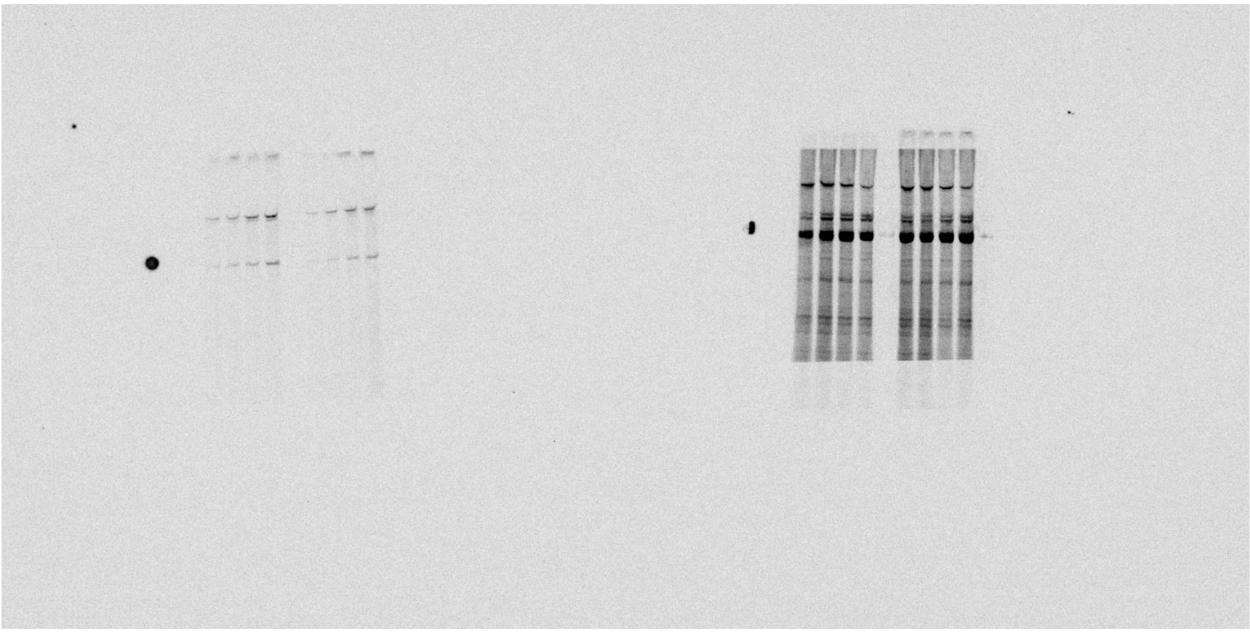

Left panel, APOB blotted from cell culture supernatant. The black dot represents 250kDa marker.

Supplementary figure 10: Unprocessed blot

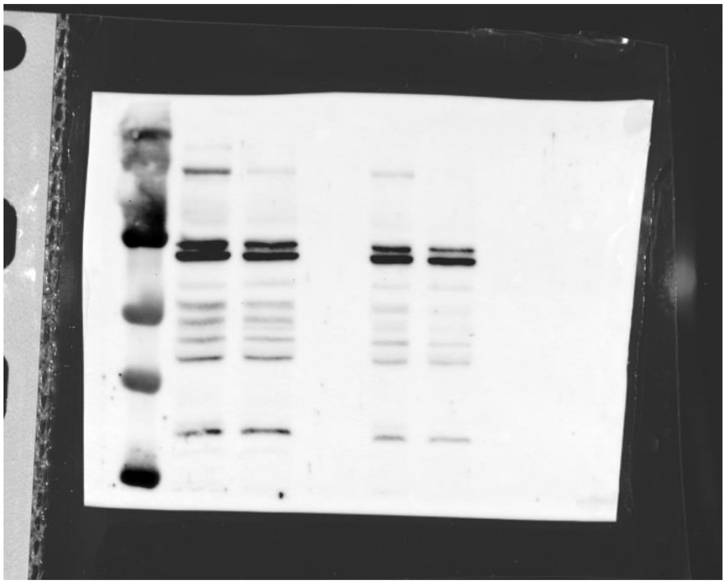

PSD3

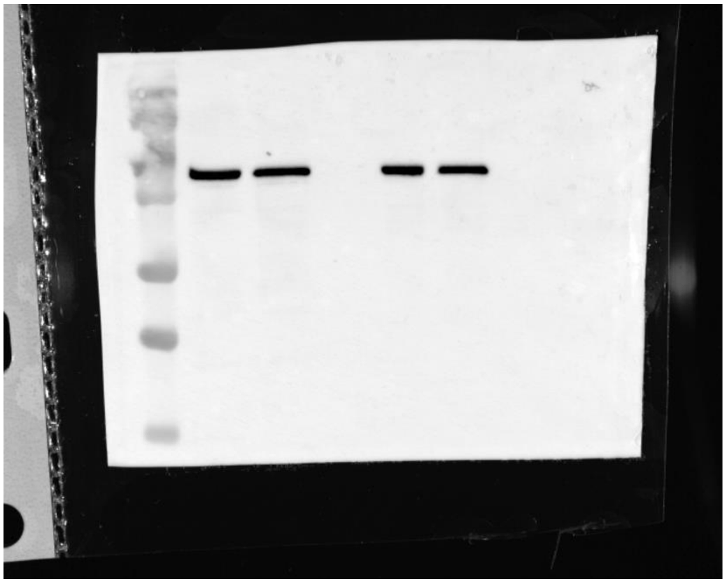

Calnexin
