# Supplementary material for: PSD3 downregulation confers protection against fatty liver disease
Source: Nat Metab. 2022 Jan 31;4(1):60–75. doi: 10.1038/s42255-021-00518-0 (PMC8803605; doi:10.1038/s42255-021-00518-0)
Supplement: Supplementary file 2 — Reporting Summary [file 42255_2021_518_MOESM2_ESM.pdf]

Corresponding author(s): Daniel Lindén, Stefano Romeo

Last updated by author(s): Dec 2, 2021

Reporting Summary

Nature Portfolio wishes to improve the reproducibility of the work that we publish. This form provides structure for consistency and transparency in reporting. For further information on Nature Portfolio policies, see our Editorial Policies and the Editorial Policy Checklist.

Statistics

For all statistical analyses, confirm that the following items are present in the figure legend, table legend, main text, or Methods section.

n/a	Confirmed	
☐	☒	The exact sample size (*n*) for each experimental group/condition, given as a discrete number and unit of measurement
☐	☒	A statement on whether measurements were taken from distinct samples or whether the same sample was measured repeatedly
☐	☒	The statistical test(s) used AND whether they are one- or two-sided<br>*Only common tests should be described solely by name; describe more complex techniques in the Methods section.*
☐	☒	A description of all covariates tested
☐	☒	A description of any assumptions or corrections, such as tests of normality and adjustment for multiple comparisons
☐	☒	A full description of the statistical parameters including central tendency (e.g. means) or other basic estimates (e.g. regression coefficient) AND variation (e.g. standard deviation) or associated estimates of uncertainty (e.g. confidence intervals)
☒	☐	For null hypothesis testing, the test statistic (e.g. *F*, *t*, *r*) with confidence intervals, effect sizes, degrees of freedom and *P* value noted<br>*Give P values as exact values whenever suitable.*
☒	☐	For Bayesian analysis, information on the choice of priors and Markov chain Monte Carlo settings
☒	☐	For hierarchical and complex designs, identification of the appropriate level for tests and full reporting of outcomes
☒	☐	Estimates of effect sizes (e.g. Cohen's *d*, Pearson's *r*), indicating how they were calculated

Our web collection on statistics for biologists contains articles on many of the points above.

Software and code

Policy information about availability of computer code

Data collection	No software was used for data collection. For UK Biobank, data collection was performed centrally.
Data analysis	The following softwares have been used to analyze data or images: IBM SPSS statistics version 27, R version 3.6.1, MATLAB R2020b (academic license), and GraphPad Prism version 9, RSEM version 1.3.0, DESeq2 1.22.2 from Bioconductor version 3.8, DESeq2 R package 2_1.6.3, AxioVision 4.8 (Zeiss), Biopix iQ software version 2.3.1, Visiopharm Integrator System software (version 2018.09).

For manuscripts utilizing custom algorithms or software that are central to the research but not yet described in published literature, software must be made available to editors and reviewers. We strongly encourage code deposition in a community repository (e.g. GitHub). See the Nature Portfolio guidelines for submitting code & software for further information.

Data

Policy information about availability of data

All manuscripts must include a data availability statement. This statement should provide the following information, where applicable:

- Accession codes, unique identifiers, or web links for publicly available datasets
- A description of any restrictions on data availability
- For clinical datasets or third party data, please ensure that the statement adheres to our policy

All data associated with this study are present in the paper or the Supplementary Information. For UK Biobank, all individual-level phenotype/genotype data are accessible via a formal application to the UK Biobank http://www.ukbiobank.ac.uk. Antisense oligonucleotides associated with this study can be made available, upon reasonable request, to academic researchers under a material transfer agreement with AstraZeneca and Ionis Pharmaceuticals. Due to study participants privacy data protection, RNA-seq data of the liver biopsies of the LBC can be made available only on request to the corresponding authors for collaborative projects. Bulk RNA seq data of the primary human hepatocytes are deposited in the NCBI SRA under the BioProject identifier PRJNA778044. All other data are available from

the authors upon reasonable request. The following online databases have been used: Database of Single Nucleotide Polymorphisms (dbSNP) https://www.ncbi.nlm.nih.gov/snp/ ; Ensembl https://www.ensembl.org/index.html ; Exome Aggregation Consortium [ExAC] database http://exac.broadinstitute.org

Field-specific reporting

Please select the one below that is the best fit for your research. If you are not sure, read the appropriate sections before making your selection.

☒ Life sciences ☐ Behavioural & social sciences ☐ Ecological, evolutionary & environmental sciences

For a reference copy of the document with all sections, see nature.com/documents/nr-reporting-summary-flat.pdf

Life sciences study design

All studies must disclose on these points even when the disclosure is negative.

Sample size

For genetic associations analyses, we performed the following power OR sample size calculations: in DHS considering 2,736 individuals and a MAF >= 0.1%, we estimated to have a power >80% to detect a 44% difference in liver fat content. To confirm our findings from the DHS in the LBC (N=1,951), we selected only variants with a MAF in Europeans >=5%. Using this strategy, we estimated to have a power >80% to detect 5% difference in the prevalence of liver steatosis. Based on our findings from the LBC, to be able to validate our results we estimated to need at least N=620 individuals in the validation cohorts and we have N=674 individuals in the “central European independent replication cohort”, and N=6,994 from the “UK Biobank” replication cohort. Thus, we estimated to have enough power to validate our findings in these 2 cohorts. Statistical power or sample size calculations have been performed using IBM SPSS samplePower 3.
For preclinical NASH studies sample sizes were determined based on a combination of pilot studies using NASH-inducing diets and published literature indicating the smallest sample sizes required to achieve sufficiently low variability and statistical significance in liver fat, inflammation, and fibrosis endpoints and to achieve robust ASO-mediated target knockdown in C57BL/6 mice. Specifically, for NASH endpoints we used the following calculation to estimate sample sizes needed:

Sample size = 2*(SD^2)*(1.96+0.842)^2/d^2 where d = effect size (difference between mean values for control and experimental groups). (Reference: Charon and Kantharia, J Pharmacology & Pharmaceuticals 2013).

Data exclusions

Exclusion criteria for all the study cohorts were pre-established as follows: for LBC and the “central European independent replication cohort”, individuals with high alcohol intake (men, >30 g/day; women, >20 g/day), viral and autoimmune hepatitis or other causes of liver disease were excluded. Individuals with missing data on histological liver disease evaluation and/or PSD3 genotype were additionally excluded from the present study. For the UK Biobank, we restricted our analysis to the subset of unrelated white-British participants from the UK Biobank, after further removal of individuals with excessive relatives (more than 10 putative third-degree relatives), a mismatch between the self-reported and genetically inferred gender, putative sex chromosome aneuploidy, withdrawn consent, and those who were identified by the UK Biobank as outliers based on heterozygosity and missingness. For the DHS, for the present study, only individuals with measurement of hepatic triglyceride content have been included. Given the low prevalence of heavy drinking (>30 g/day) in this cohort, we did not exclude subjects based on alcohol intake. All analyses were based on cross-sectional data. No other exclusion criteria compared to the original study design.

Replication

To confirm our findings from the DHS in the LBC (N=1,951), we selected only variants with a MAF in Europeans >=5%. Using this strategy, we estimated to have a power >80% to detect 5% difference in the prevalence of liver steatosis. Based on our findings from the LBC, to be able to validate our results we estimated to need at least N=620 individuals in the validation cohorts and we have N=674 individuals in the “central European independent replication cohort”, and N=6,994 from the “UK Biobank” replication cohort. Thus, we estimated to have enough power to validate our findings in these 2 cohorts. Statistical power or sample size calculations have been performed using IBM SPSS samplePower 3.

Randomization

For in vivo studies, mice were randomized into study groups based on body weight and plasma ALT levels. For genetic studies, individuals have been allocated into the study groups based on the target genotype. Confounding factors (including, age, gender, BMI plus all the others relevant for each specific study) were controlled including these as covariates into the statical models. For in vitro studies, primary cells have been allocated into the study groups based on the target genotype; immortalized cells have been allocated into the study groups randomly.

Blinding

Histological evaluation of liver disease both in Human and in Murine samples has been performed by experienced histopathologists blinded from patients or study group condition and genotype.

Reporting for specific materials, systems and methods

We require information from authors about some types of materials, experimental systems and methods used in many studies. Here, indicate whether each material, system or method listed is relevant to your study. If you are not sure if a list item applies to your research, read the appropriate section before selecting a response.

Materials & experimental systems

n/a	Involved in the study
☐	☒ Antibodies
☐	☒ Eukaryotic cell lines
☒	☐ Palaeontology and archaeology
☐	☒ Animals and other organisms
☐	☒ Human research participants
☒	☐ Clinical data
☒	☐ Dual use research of concern

Methods

n/a	Involved in the study
☒	☐ ChIP-seq
☒	☐ Flow cytometry
☒	☐ MRI-based neuroimaging

Antibodies

Antibodies used

Anti Calnexin (rabbit, for WB) (Sigma Aldrich C4731), Anti-Arf6 (mouse, for WB) provided from activation kit (Cell Biolabs Inc. STA-407-6), Anti-PSD3 (for WB) (Custom made), anti-active ARF6 (mouse, for IHC) (NewEast biosciences 26918), anti-PSD3(rabbit, for IHC) (ProSci 29-749), Anti-rabbit IgG HRP (for WB) (GE healthcare NA934V), Anti-Mouse IgG HRP(for WB) (GE healthcare NA931V), Goat Anti-Mouse Cat.#170-6516 Bio-Rad Laboratories (secondary Ab for IHC),Goat Anti-Rabbit Cat. #1706515 Bio-Rad Laboratories (secondary Ab for IHC).

Validation

The PSD3 custom Ab was validated via silencing of endogenously expressed PSD3 in human hepatoma HepaRG cells (as described in the manuscript and in Supplementary figure 10).
For other antibodies, Species, application and any validation protocols/statements provided in the manufacturers website is stated below:

Anti Calnexin (rabbit) (Sigma Aldrich C4731)
Species: Rabbit, Tested applications: IP, Indirect immunofluorescence, microarray, western blot.
Species reactivity: canine, mouse, human, rat. Antibody validated by HeLa cells (human) extract separated on SDS-PAGE and probed with IgG fraction AB to calnexin (rabbit,cat no. C4731). The antibody was developed with Alk Phos APA Rabbit IgG(gt) Cat no, A9919) and a NBT/BCIP substrate.
species reactivity

Anti-Arf6 (mouse) provided from activation kit (Cell Biolabs Inc. STA-407-6)
The antibody reacts with Arf6 from human, bovine, canine, and rat. Antibody validated by western blot of HeLa cell lysate loaded with GDP and incubated with GGA3 PBD Agarose beads and HeLa cell lysate loaded with GTPγS and incubated with GGA3 PBD Agarose beads.

Anti-active ARF6 (mouse) (NewEast biosciences 26918)
Species: Mouse, Tested application: IP, IHC, Species reactivity: Anti-active Arf6 antibody recognizes active Arf6 from vertebrates. Antibody validated by Purified full-length Arf6 proteins immunoprecipitated after treated with GDP or GTPγS. Immunoprecipitation was done with the anti-active Arf6 monoclonal antibody (Cat. # NEBA10223,Vita scientific). Immunoblot was with an anti-Arf6 polyclonal antibody (Cat. #NEBA10020,Vita scientific).

Anti-PSD3(rabbit) (ProSci 29-749)
Species: Rabbit, Tested applications: ELISA, IHC, WB, Immunogen: Antibody produced in rabbits immunized with a synthetic peptide corresponding a region of human PSD3. Species reactivity: Human, mouse, rat. Antibody validated by human muscle on IHC and western blot on human A204 cells at 2.5µg/mL.

Amersham ECL Anti-rabbit IgG, Horseradish Peroxidase Linked Species-Specific Whole Antibody (from donkey) (GE healthcare NA934V)
Species: Donkey, Tested applications: Western blotting, ELISA, immunocytochemistry, Species reactivity: Rabbit. The antibody is prepared by hyper-immunizing donkeys with purified immunoglobulin fractions from normal rabbit serum to produce high affinity antibodies. Every batch is also QC tested in a Western blotting system. This is performed using Hybond™ ECL™ membrane containing serially diluted Beta-galactosidase protein and immundetected with primary antibody Anti-Beta-galactosidase and secondary antibody NA934, anti-rabbit HRP. Blots are detected using ECL and ECL Plus™ detection systems.

Amersham ECL Anti-mouse IgG, peroxidase-linked whole antibody (from sheep) affinity purified general purpose reagent (GE healthcare NA931V)
Species: Sheep. Tested applications: western blotting. Species reactivity: Mouse.
The antibody is prepared by hyper-immunizing sheep with purified immunoglobulin fractions from normal mouse serum to produce high affinity antibodies. Every batch is also QC tested in a Western blotting system. This is performed using Hybond™ ECL™ membrane containing tubulin protein and immunodetected with: primary antibody, Monoclonal anti-tubulin; and secondary antibody NXA931, anti-mouse IgG, HRP GPR. Blots are detected using ECL and ECL Plus™ detection systems

Goat anti Mouse IgG (H/L) polyclonal antibody (Cat no. 170-6516 Bio-Rad Laboratories)
Species: Goat, Tested applications: western blotting (Where this product has not been tested for use in a particular technique this does not necessarily exclude its use in such procedures). Species reactivity: mouse. Antiserum to mouse IgG (H/L) was raised by repeated immunisation of goats with highly purified antigen. Antibody reacts with the heavy chains of mouse IgG, and the light chains common to the majority of mouse immunoglobulins. This antibody has not been cross adsorbed against other mouse immunoglobulins, and has been found to react substantially with antibody light chains common to all mouse antibodies. Therefore, significant cross reactivity to non-IgG primary antibodies may occur. Antibody validated by detection of recombinant Human CD33 with Mouse anti Human CD33 (MCA1271, Bio-Rad) in a Western blot analysis with Goat anti Mouse IgG:HRP (Cat no. 170-6516) at a

1/10000 dilution as the secondary antibody.

Goat anti Rabbit IgG antibody (Cat no. 1706515 Bio-Rad Laboratories)
Species: goat, Tested applications; Immunohistology (frozen), ELISA, western blotting. Species reactivity: rabbit. Antisera to rabbit IgG were raised by repeated immunisations of goats with highly purified antigen. Purified IgG was prepared from whole serum by affinity chromatography.

Eukaryotic cell lines

Policy information about cell lines

Cell line source(s)	McA RH-7777 cell line was purchased from ATCC; human hepatocytes Huh7 cell line was purchased from JCRB cell bank, Japan; Sf21 cells were purchased from Gibco(Cat no 11497013)
Authentication	None of the cell lines used were authenticated.
Mycoplasma contamination	All the cell lines used were tested negative for mycoplasma by providers
Commonly misidentified lines (See ICLAC register)	No commonly misidentified cell lines were used in the study.

Animals and other organisms

Policy information about studies involving animals; ARRIVE guidelines recommended for reporting animal research

Laboratory animals	For in vivo studies six-week-old male C57BL/6 mice were used. All mice were obtained from the Jackson Laboratory (Bar Harbor, ME) and housed in cages on a 12-h/12-h light/dark cycle at 22.5±2.5 °C and 50±20% humidity, fed ad libitum and had free access to drinking water for the duration of the studies.<br>For custom PSD3 antibody generation three male, 22 weeks old New Zealand white rabbits were used.
Wild animals	The study did not involve wild animals
Field-collected samples	The study did not involve samples collected on the field
Ethics oversight	The in vivo studies in mice were compliant with ethical regulation and approved by an institutional animal care and use committee and the IONIS local ethical committee, AAALAC Accreditation #P-0305.

Note that full information on the approval of the study protocol must also be provided in the manuscript.

Human research participants

Policy information about studies involving human research participants

Population characteristics	For the DHS: N=2736 individuals with mean age 46±10 years, mean BMI 29±5 kg/m2.and a total of 1533 individuals were female (56%); 1328 (48%) were African Americans, 882 (32%) were European Americans and 465 (17%) were Hispanics.<br>For the LBC: a total of 1951 individuals with mean age 47±12 year, mean BMI 37±9 kg/m2, male gender n=909 (47%); of these individuals, n=1,022 (52%) were from University of Milan, Milan, Italy, n=374 (19%) from the Palermo University Hospital, Palermo, Italy, =410 (21%) from the Northern Savo Hospital District, Kuopio, Finland and n=145 (7%) from the Hospital District of Helsinki and Uusimaa, Finland.<br>Fore the Central European Cohort, a total of 674 individuals with mean age 45±12 years, mean BMI 46±10 kg/m2, male gender n=236 (35%). Of these individuals, 559 (53%) were from Germany, 83 (12%) from Austria and 32 (5%) from Switzerland.<br>For the UK Biobank: a total of 6,994 unrelated white British adult individuals were analyzed in the present study with mean age 55±7 years, mean BMI 27±4 kg/m2, male gender n=3,392 (48%).
Recruitment	The DHS is a multi-ethnic population-based sample of the Dallas County residents including individuals from three different ethnic groups, enrolled between 2000 and 2002. A probably sample of noninstitutionalized, English- or Spanish-speaking adult individuals aged 18-65 years.<br>The primary basis for the sampling frame was the US Postal Service delivery sequence file. A probability sample with equal numbers of unrelated black and non-black women and men, was recruited. More details on the study design and recruitment can be found in the paper from Ronald G et al, the Am J of Cardiology (2004).<br>The LBC is a cross-sectional study of adult individuals of European descent who underwent liver biopsy for suspected non-alcoholic steatohepatitis (NASH) or severe obesity, consecutively enrolled in 3 independent European centres. To these, a total of 323 individuals from a fourth and independent Finnish centre has been included. Individuals with high alcohol intake

(men, >30 g/day; women, >20 g/day), viral and autoimmune hepatitis or other causes of liver disease were excluded. More details on original study design and recruitment process can be found in the paper from Mancina RM et al., Gastroenterology (2016), Dongiovanni P et al., Hepatology (2015), and Luukkonen PK et al., J Hepatol 64, 1167-1175 (2016).
The UK Biobank is a large population-based prospective study comprising more than 500,000 adult individuals (aged between 40-69 years at recruitment) who visited 22 recruitment centres throughout the UK between 2006 and 2010. For the overall study design and recruitment process please visit https://www.ukbiobank.ac.uk/media/gnkeyh2q/study-rationale.pdf .
For the present study, we restricted our analysis (cross-sectional) to a subset of unrelated white-British participants from the UK Biobank, after further removal of individuals with excessive relatives (more than 10 putative third-degree relatives), a mismatch between the self-reported and genetically inferred gender, putative sex chromosome aneuploidy, withdrawn consent, and those who were identified by the UK Biobank as outliers based on heterozygosity and missingness.
The central European cohort is a cross-sectional study of adult obese individuals with BMI > 30 kg/m2 who underwent percutaneous or surgical liver biopsy. Individuals were consecutively recruited from tertiary referral centers in: Austria, Germany and Switzerland. Individuals with infectious (e.g. viral hepatitis, HIV), immunological, drug-induced hepatic steatosis (e.g. amiodarone, methotrexate, steroids, valproate, etc.) or hereditary causes (hereditary hemochromatosis, Wilson disease) of chronic liver disease were excluded by accepted measures. Individuals with self-reported average alcohol consumption > 30 g/day (in men) or 20 g/day (in women) were excluded.

Ethics oversight

The DHS was approved by the Institutional Review Board of University of Texas Southwestern Medical Centre. The LBC study was approved by the Ethics Committees of the Fondazione IRCCS Ca' Granda (Milan), Palermo University Hospital (Palermo), Northern Savo Hospital District in Kuopio (Finland), and the ethics committee of the Hospital District of Helsinki and Uusimaa (Finland).
The UK Biobank study received ethical approval from the National Research Ethics Service Committee North West Multi-Centre Haydock (reference 16/NW/0274).
The Central European cohort was approved from local ethics committees of the participating centers.
All patients gave their written informed consent.
All studies were performed in accordance with the current version of the Helsinki Declaration.

Note that full information on the approval of the study protocol must also be provided in the manuscript.
