# Supplementary figures and images for: PSD3 downregulation confers protection against fatty liver disease
Source: Nat Metab. 2022 Jan 31;4(1):60–75. doi: 10.1038/s42255-021-00518-0 (PMC8803605; doi:10.1038/s42255-021-00518-0)

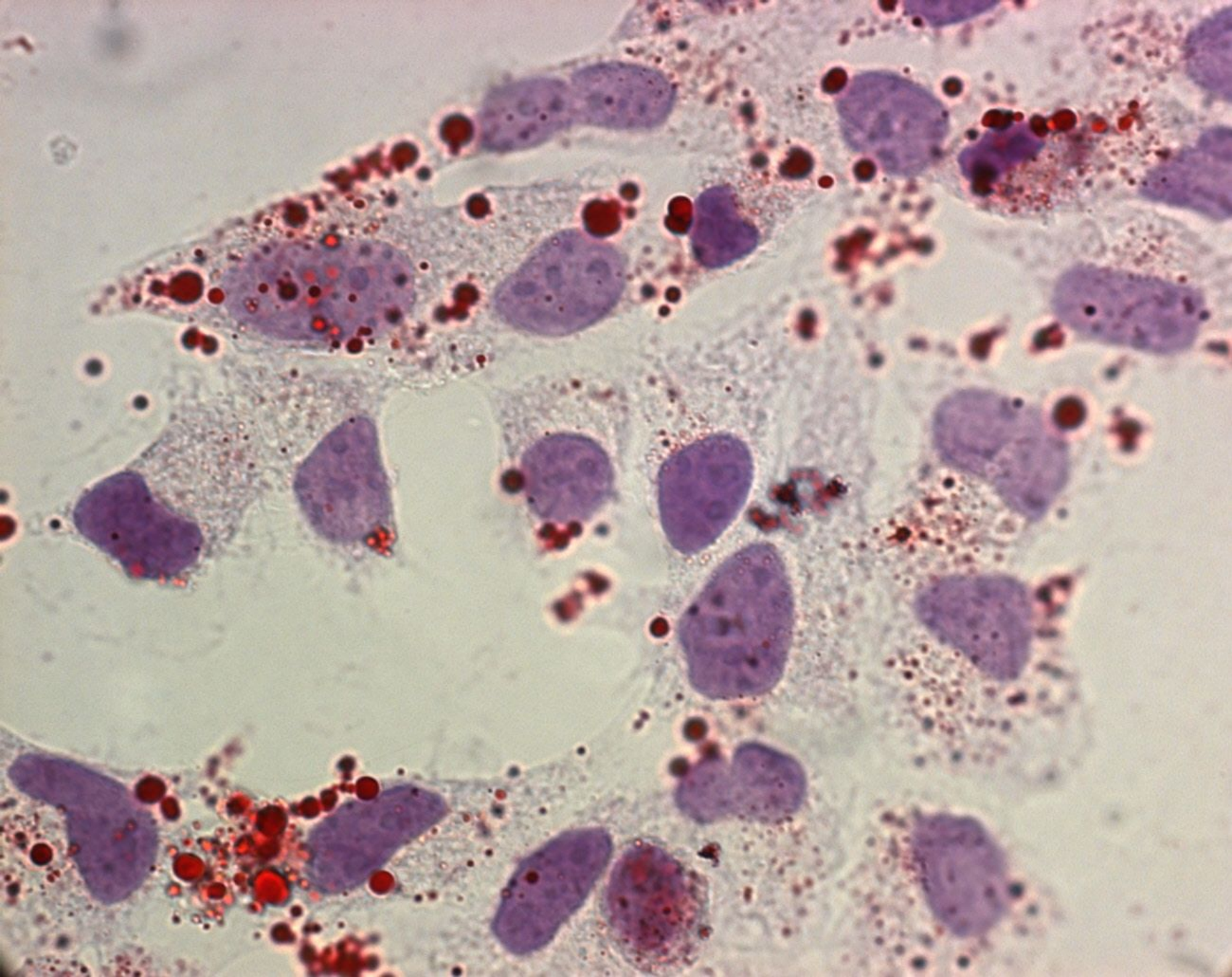

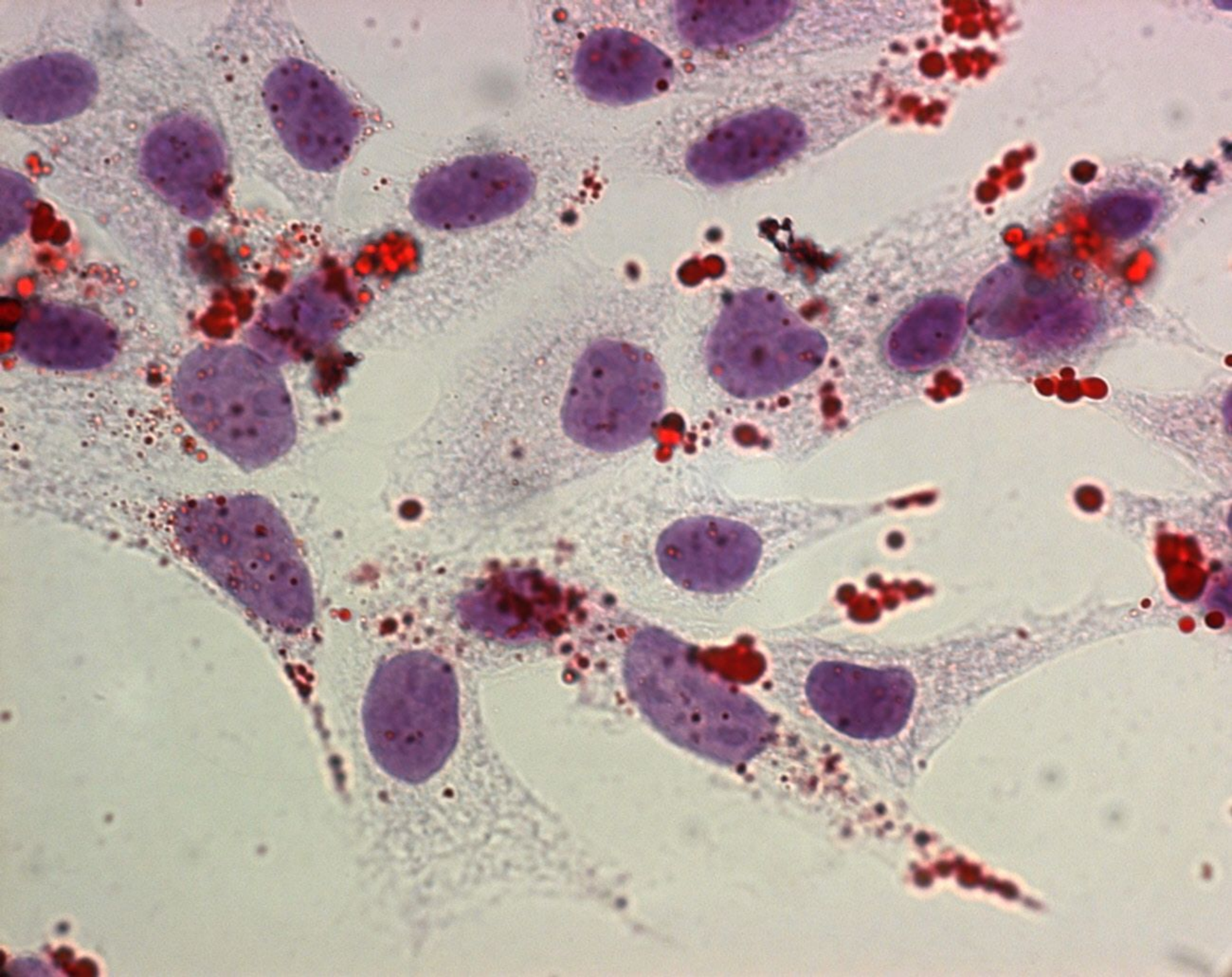

Supplement: Supplementary file 5 — Combined microscopy image for Supplementary Fig. 5 as a PDF. [file 42255_2021_518_MOESM5_ESM.pdf]

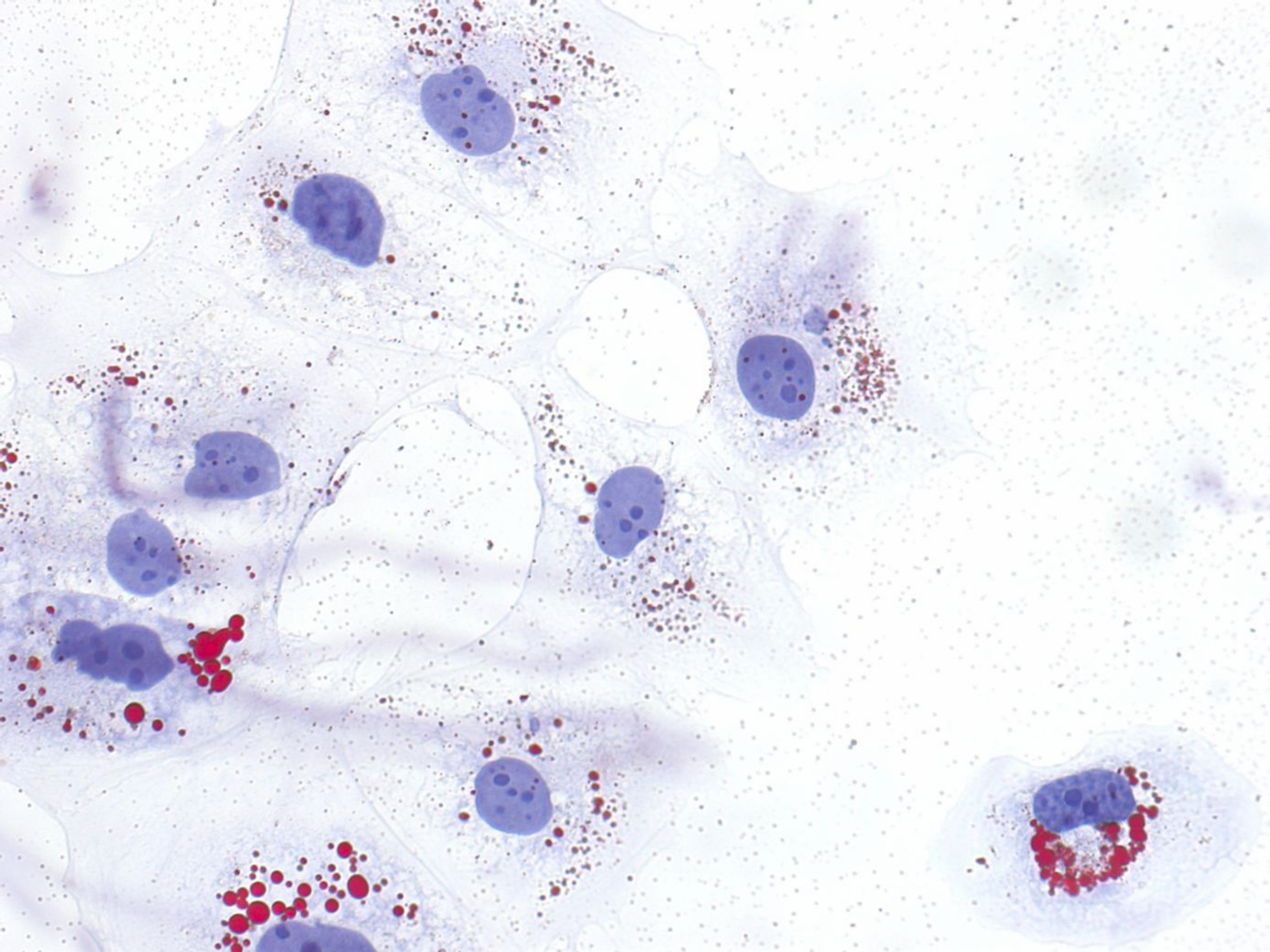

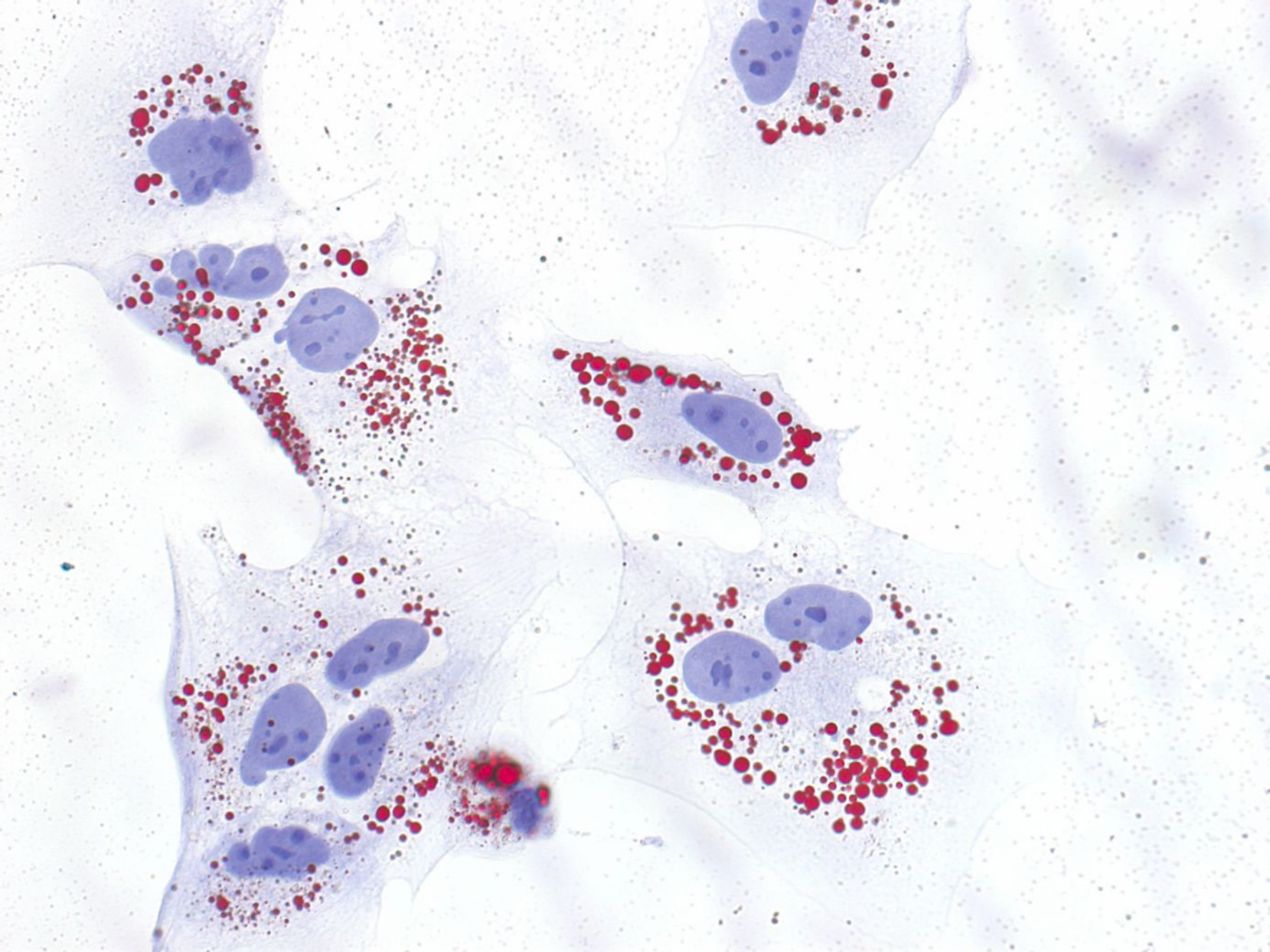

Supplement: Supplementary file 7 — Combined microscopy image for Supplementary Fig. 6 as a PDF. [file 42255_2021_518_MOESM7_ESM.pdf]

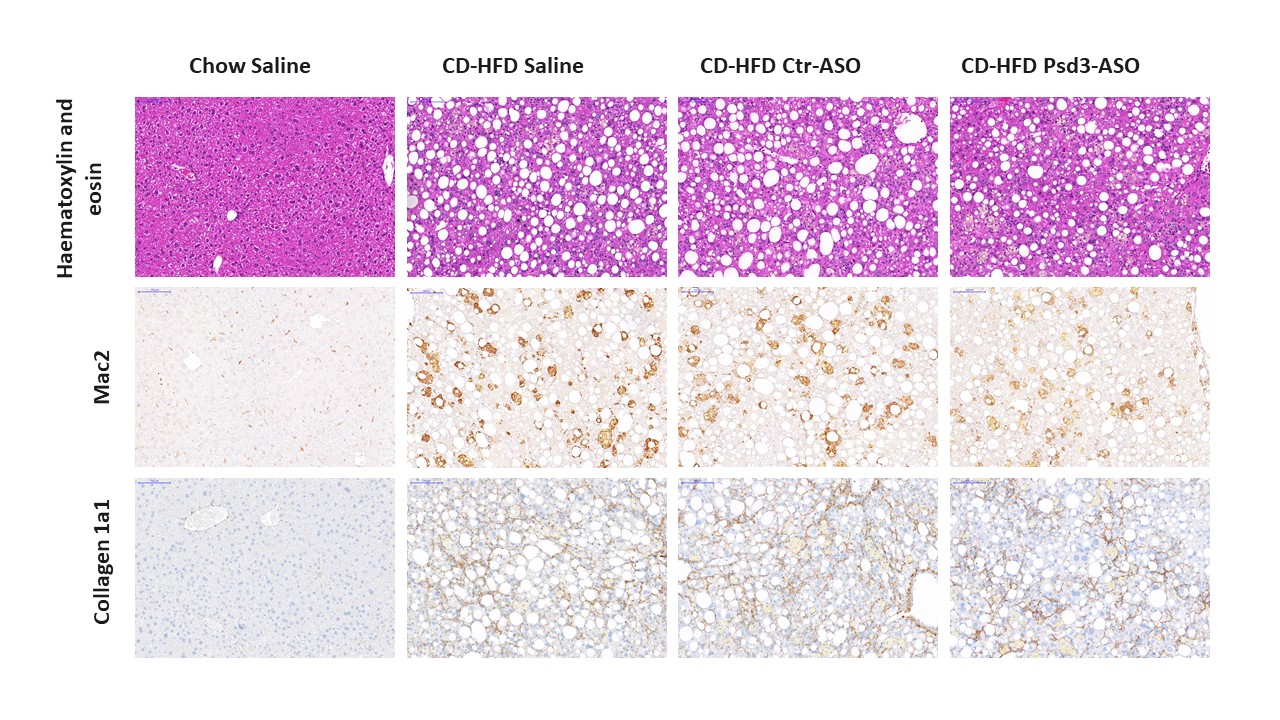

Supplement: Supplementary file 10 — Microscopy image for Supplementary Fig. 9. [file 42255_2021_518_MOESM10_ESM.jpg]

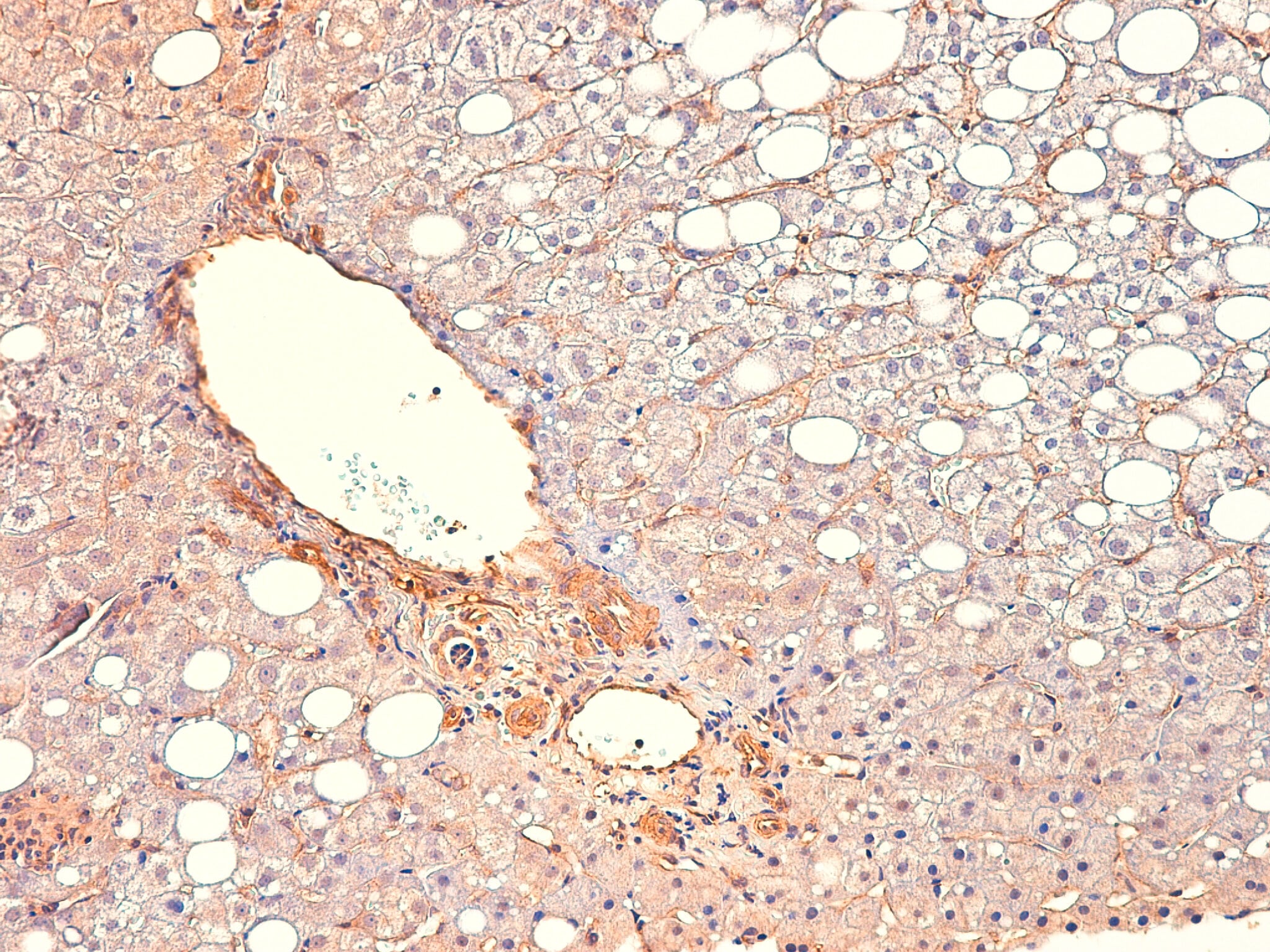

Supplement: Supplementary file 12 — Microscopy images for Supplementary Fig. 7. [file 42255_2021_518_MOESM12_ESM.zip › PSD3 HET 1.jpg]

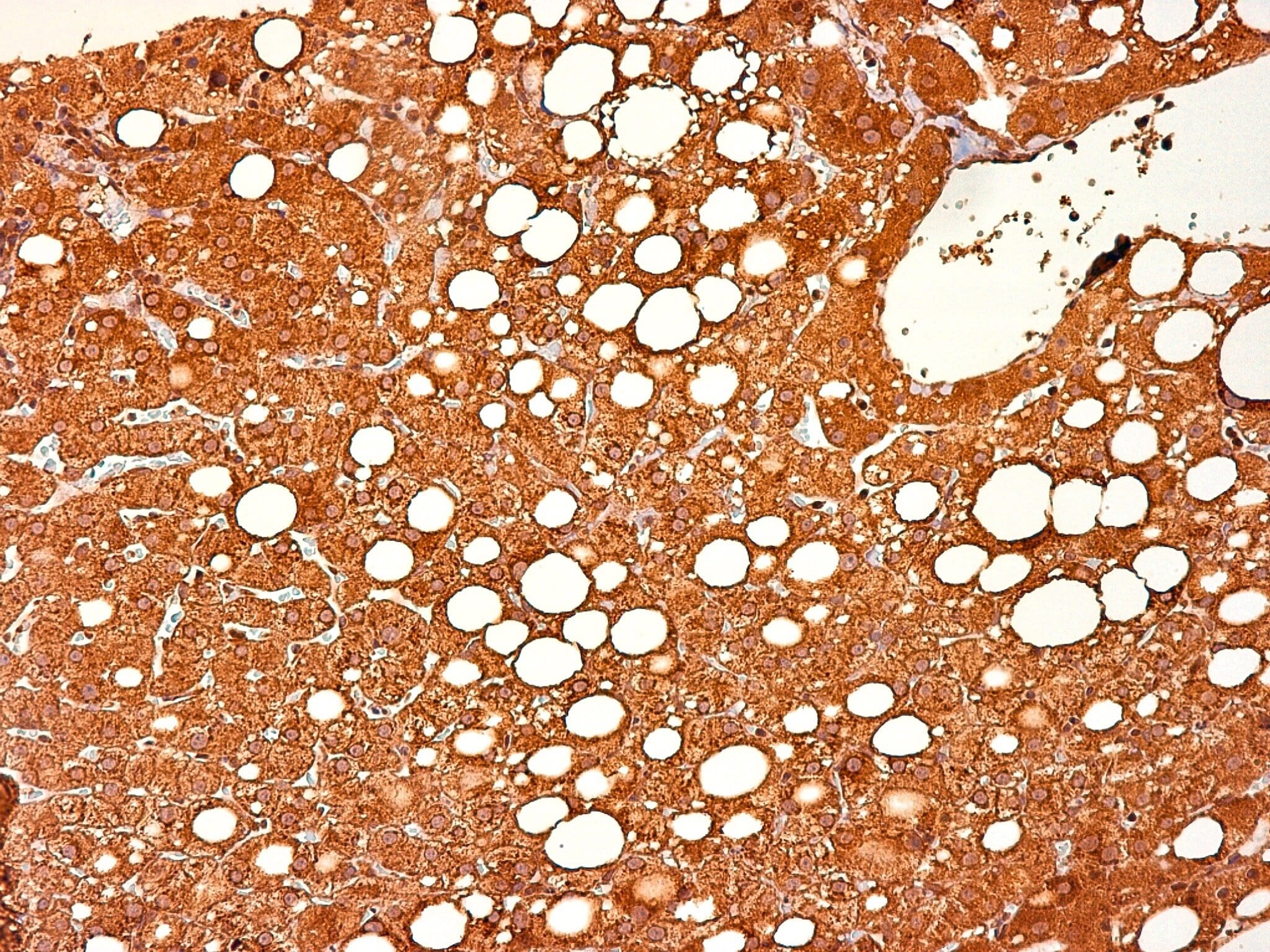

Supplement: Supplementary file 12 — Microscopy images for Supplementary Fig. 7. [file 42255_2021_518_MOESM12_ESM.zip › PSD3 HET 1_ARF6.jpg]

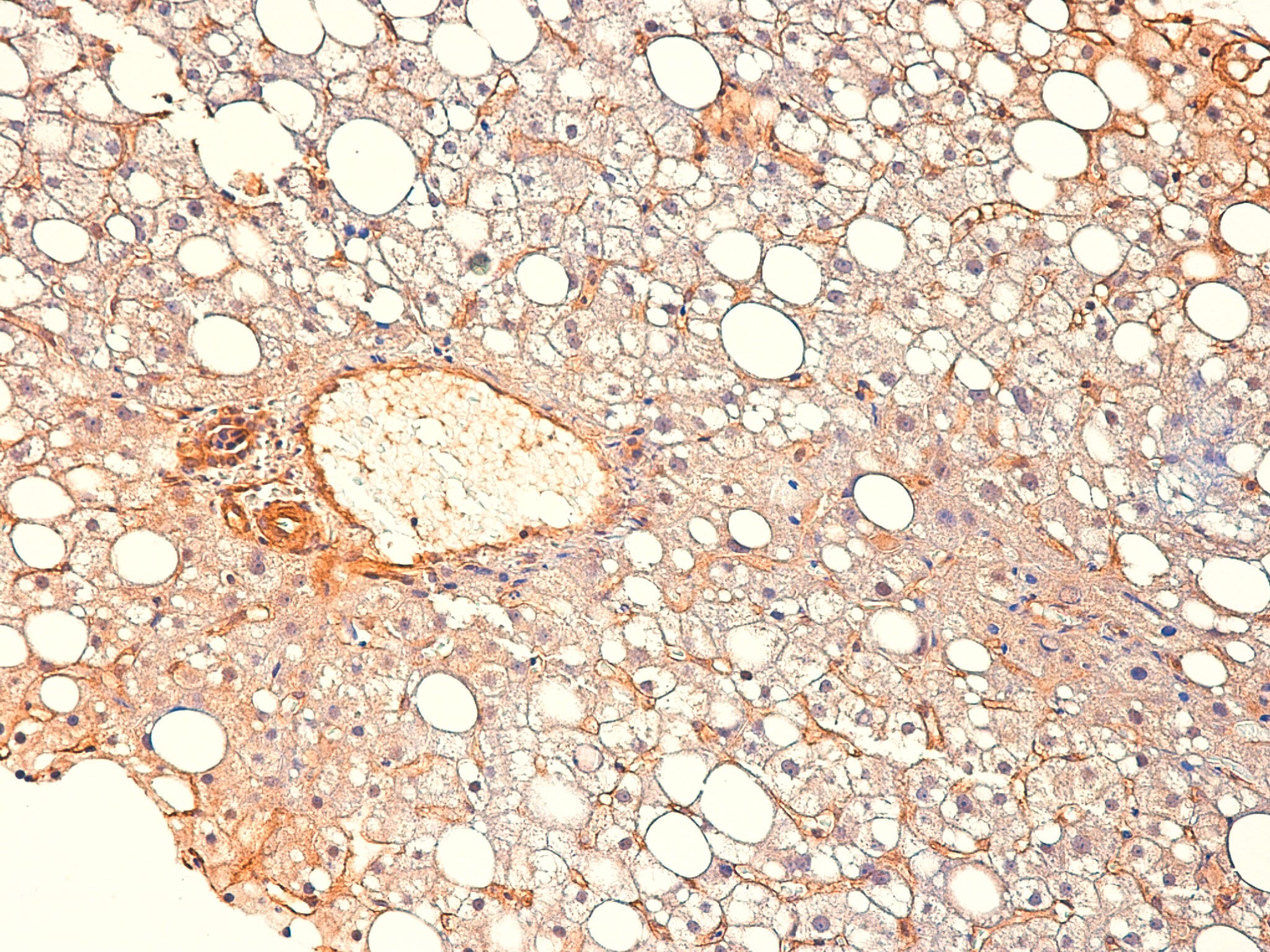

Supplement: Supplementary file 12 — Microscopy images for Supplementary Fig. 7. [file 42255_2021_518_MOESM12_ESM.zip › PSD3 HET 2.jpg]

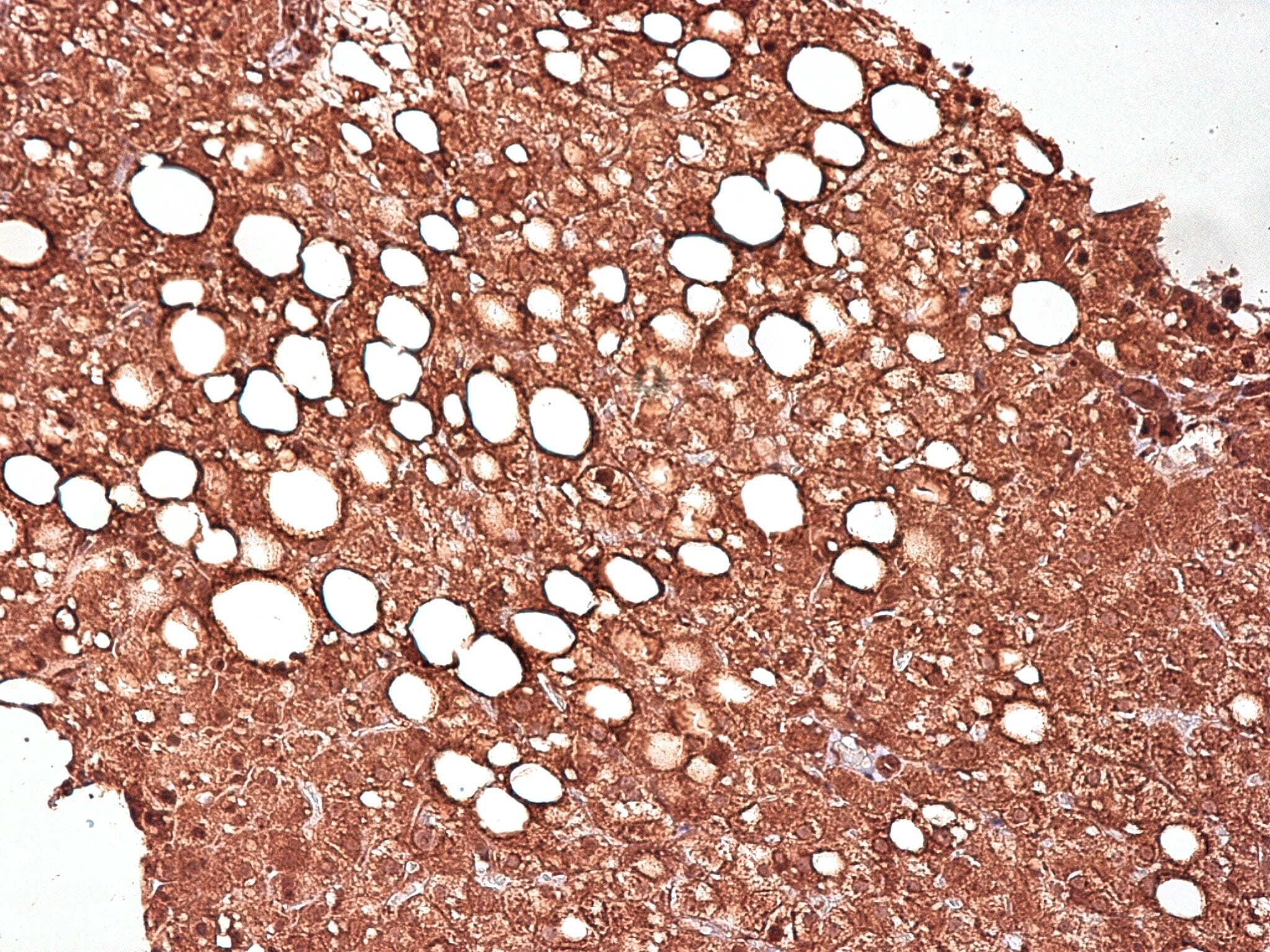

Supplement: Supplementary file 12 — Microscopy images for Supplementary Fig. 7. [file 42255_2021_518_MOESM12_ESM.zip › PSD3 HET 2_ARF6.jpg]

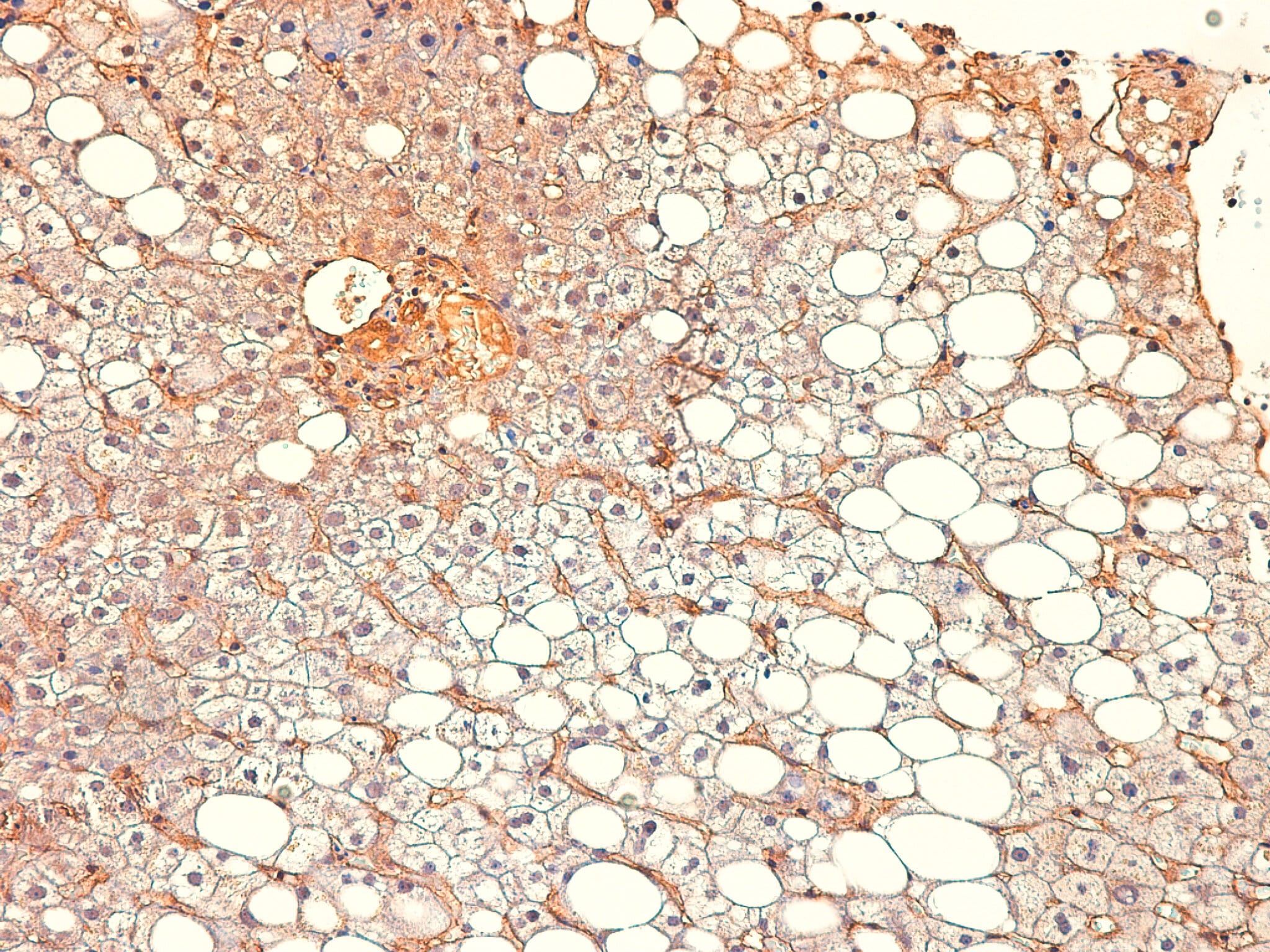

Supplement: Supplementary file 12 — Microscopy images for Supplementary Fig. 7. [file 42255_2021_518_MOESM12_ESM.zip › PSD3 HET 3.jpg]

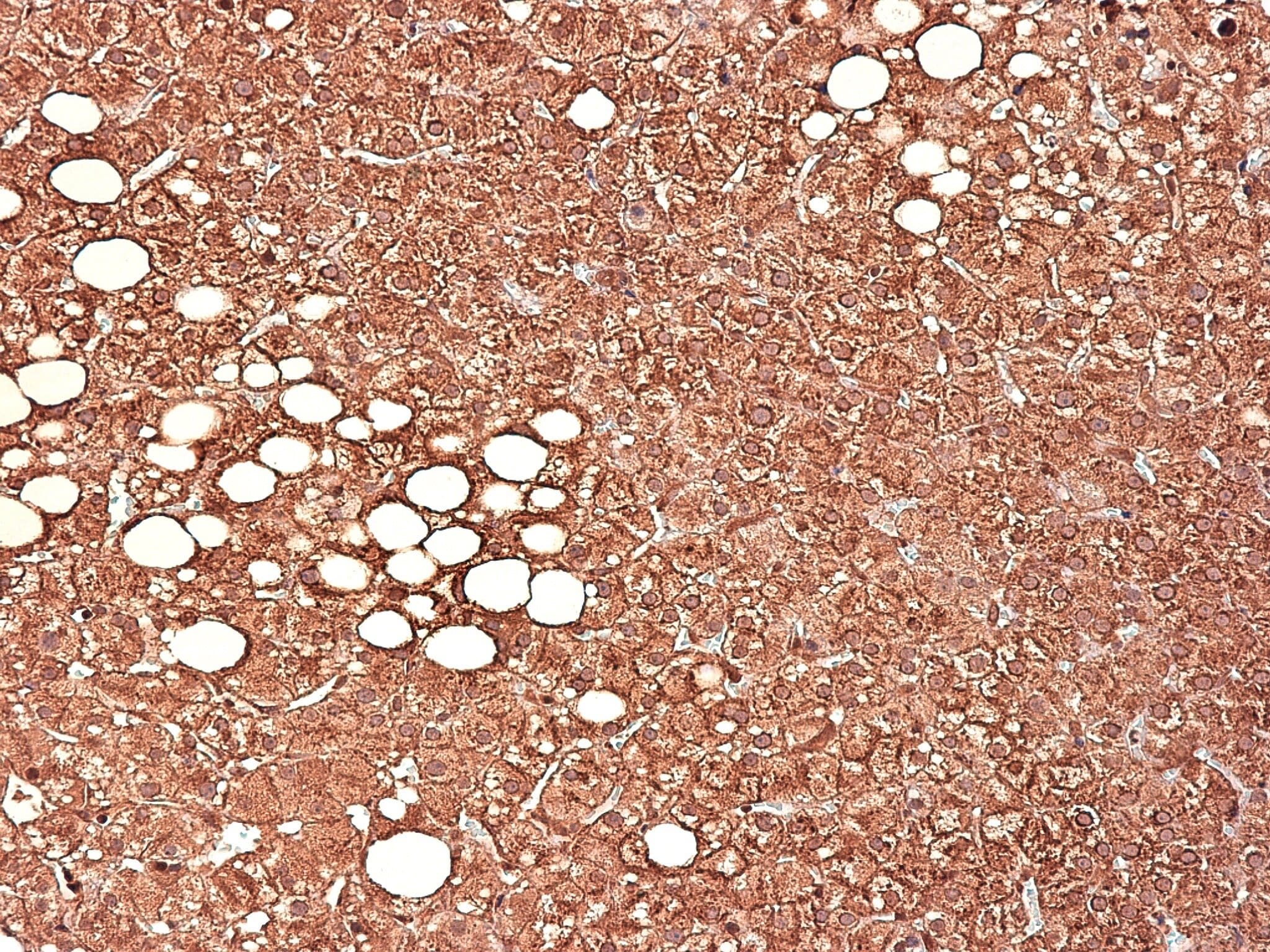

Supplement: Supplementary file 12 — Microscopy images for Supplementary Fig. 7. [file 42255_2021_518_MOESM12_ESM.zip › PSD3 HET 3_ARF6.jpg]

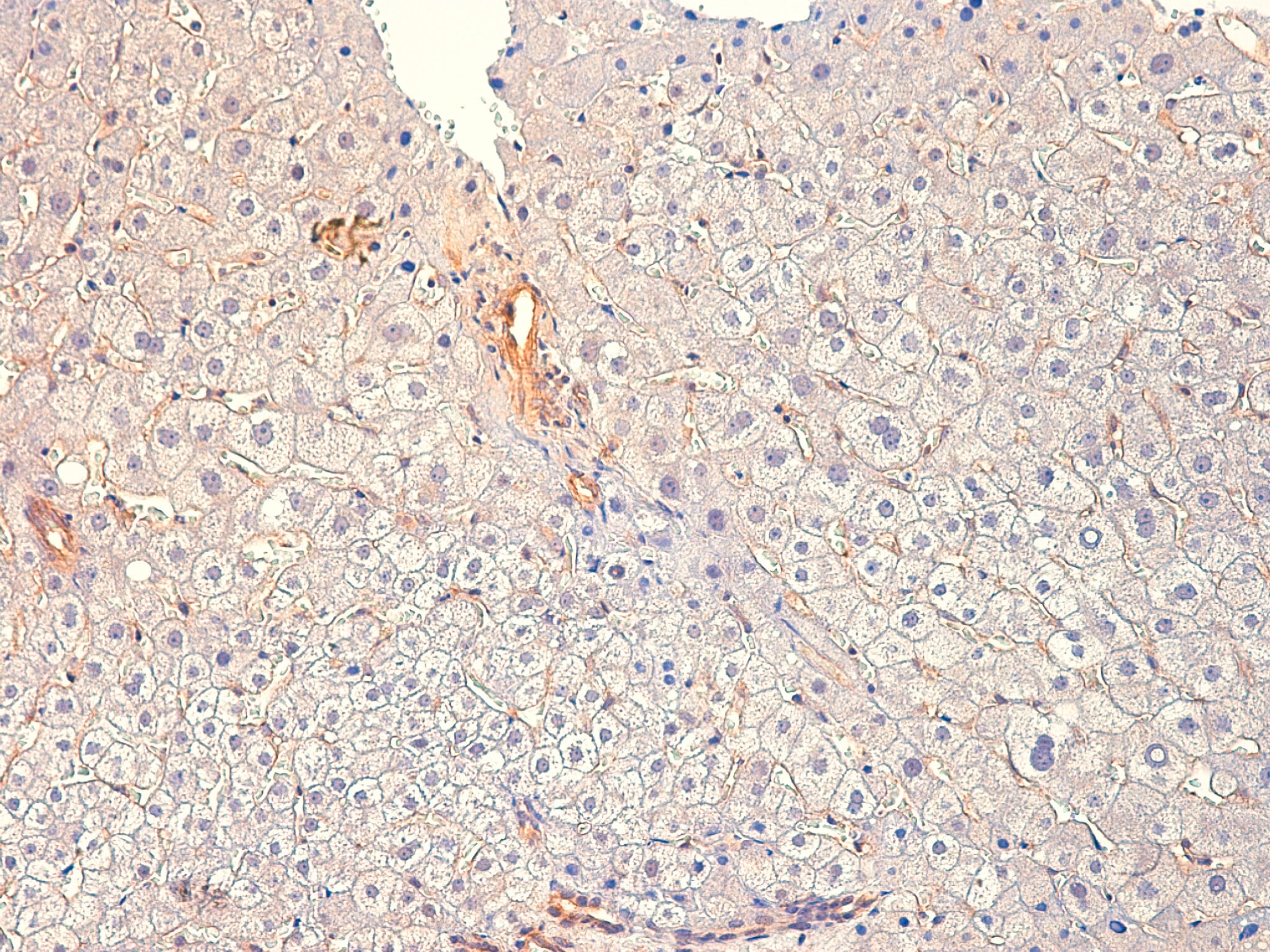

Supplement: Supplementary file 12 — Microscopy images for Supplementary Fig. 7. [file 42255_2021_518_MOESM12_ESM.zip › PSD3 HET 4.jpg]

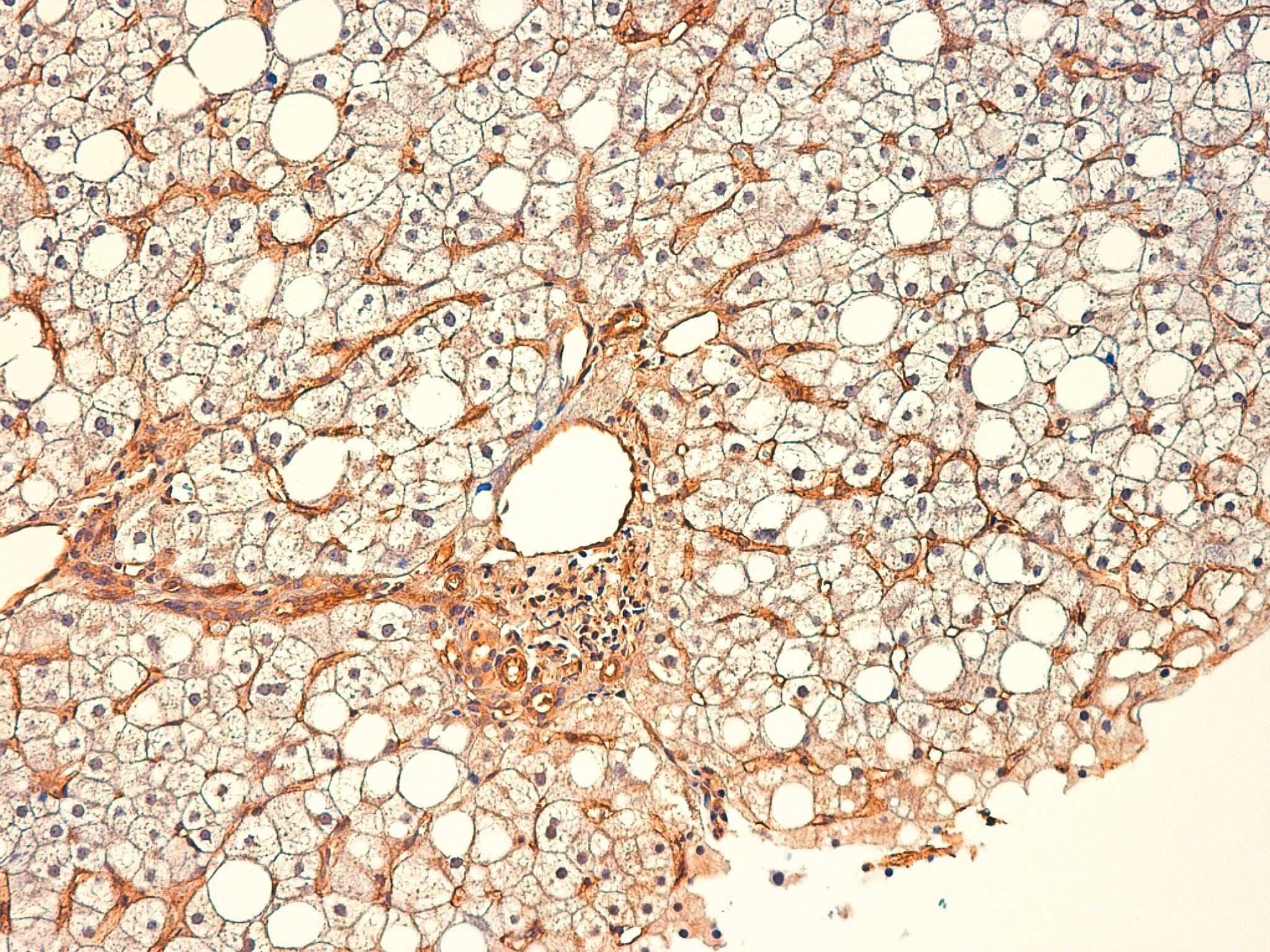

Supplement: Supplementary file 12 — Microscopy images for Supplementary Fig. 7. [file 42255_2021_518_MOESM12_ESM.zip › PSD3 HOM 1.jpg]

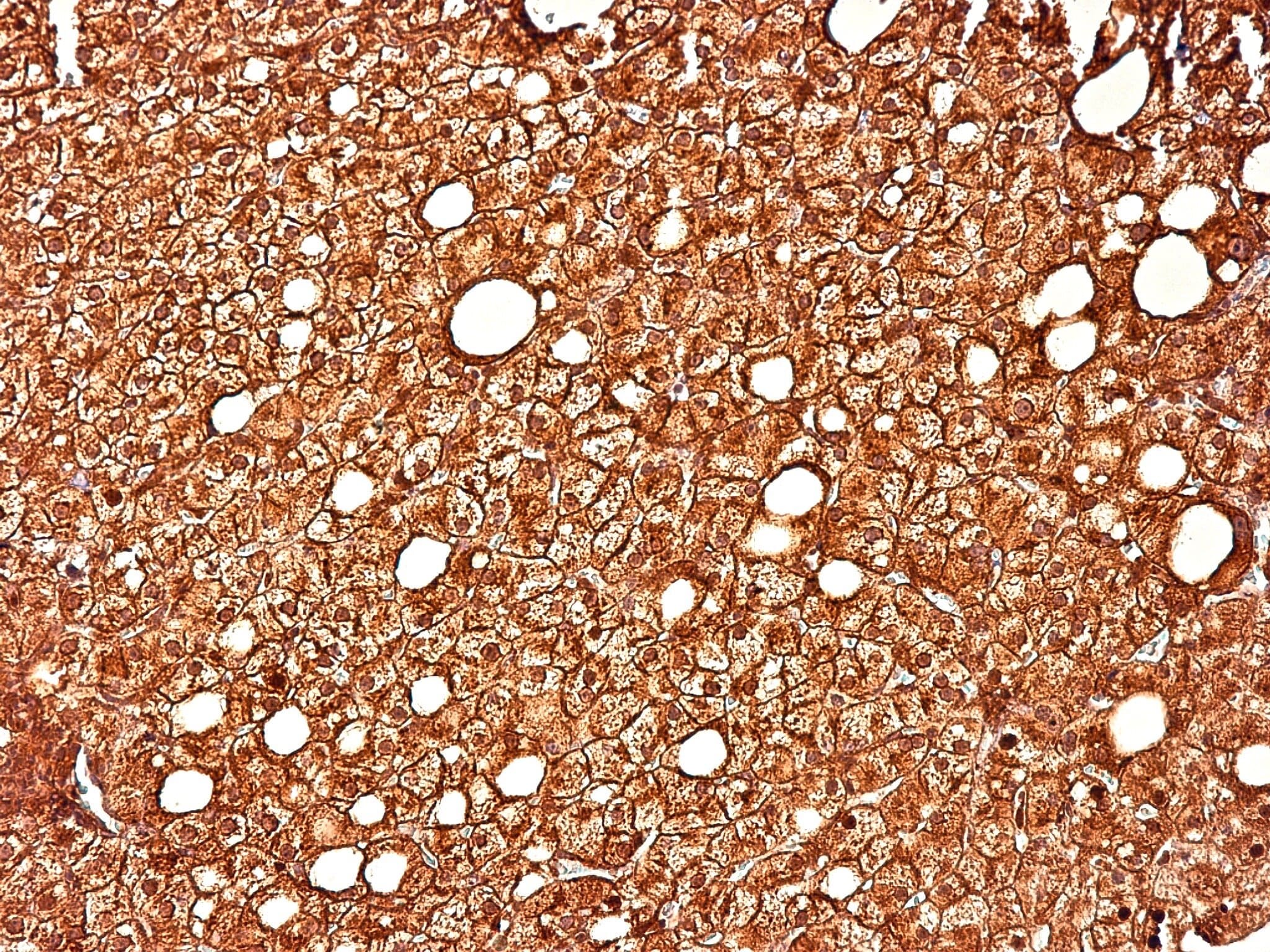

Supplement: Supplementary file 12 — Microscopy images for Supplementary Fig. 7. [file 42255_2021_518_MOESM12_ESM.zip › PSD3 HOM 1_ARF6.jpg]

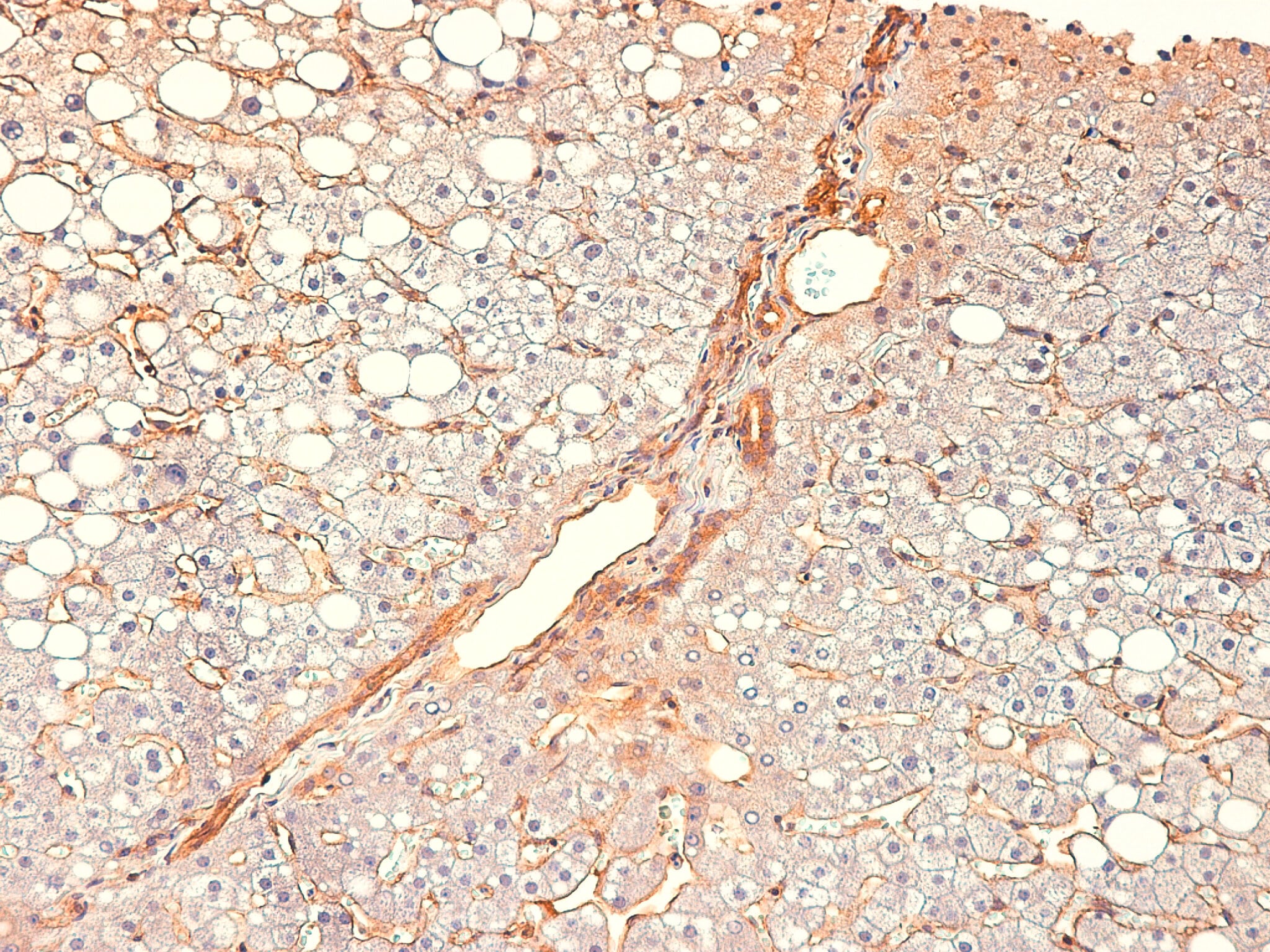

Supplement: Supplementary file 12 — Microscopy images for Supplementary Fig. 7. [file 42255_2021_518_MOESM12_ESM.zip › PSD3 HOM 2.jpg]

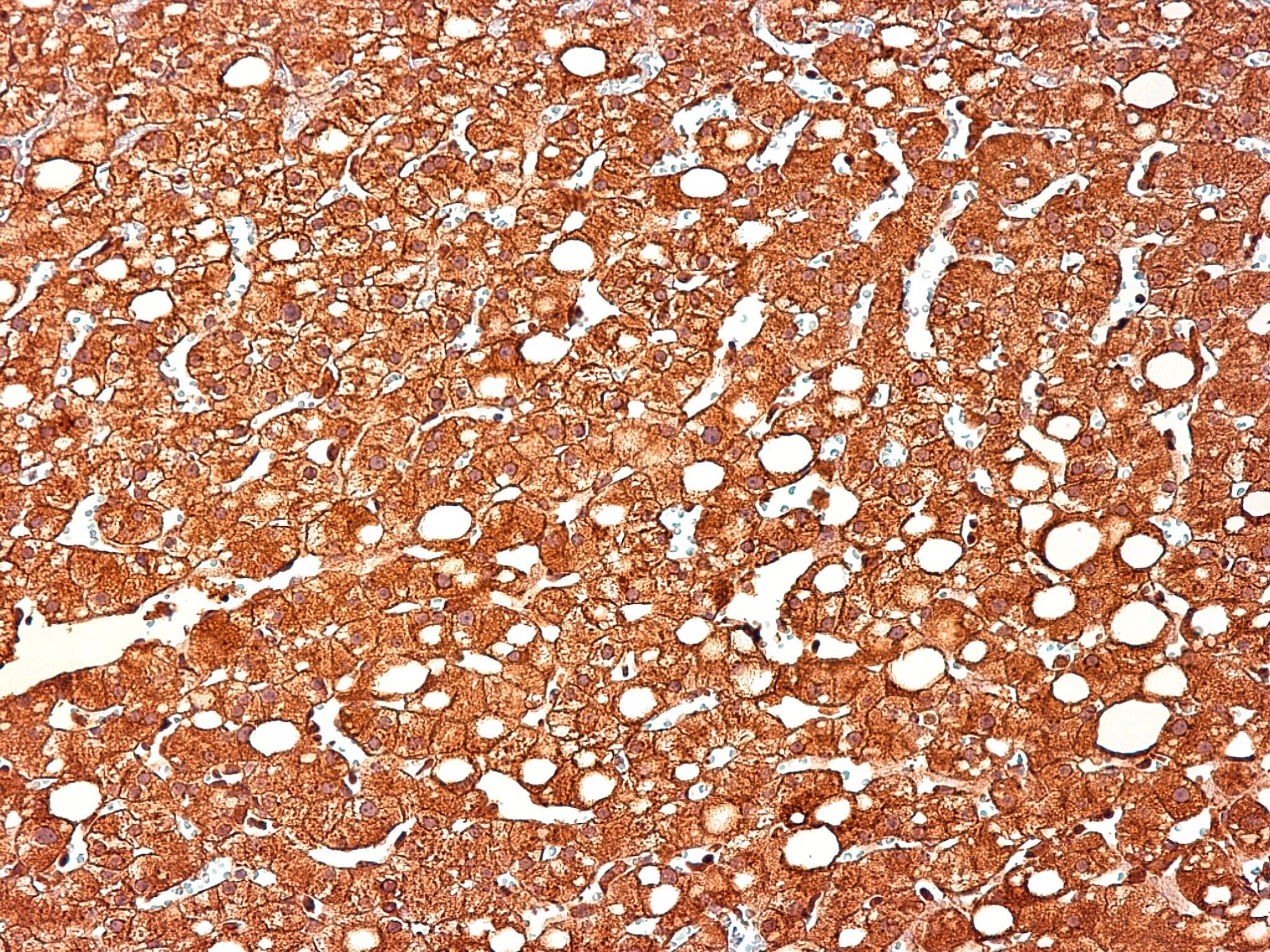

Supplement: Supplementary file 12 — Microscopy images for Supplementary Fig. 7. [file 42255_2021_518_MOESM12_ESM.zip › PSD3 HOM 2_ARF6.jpg]

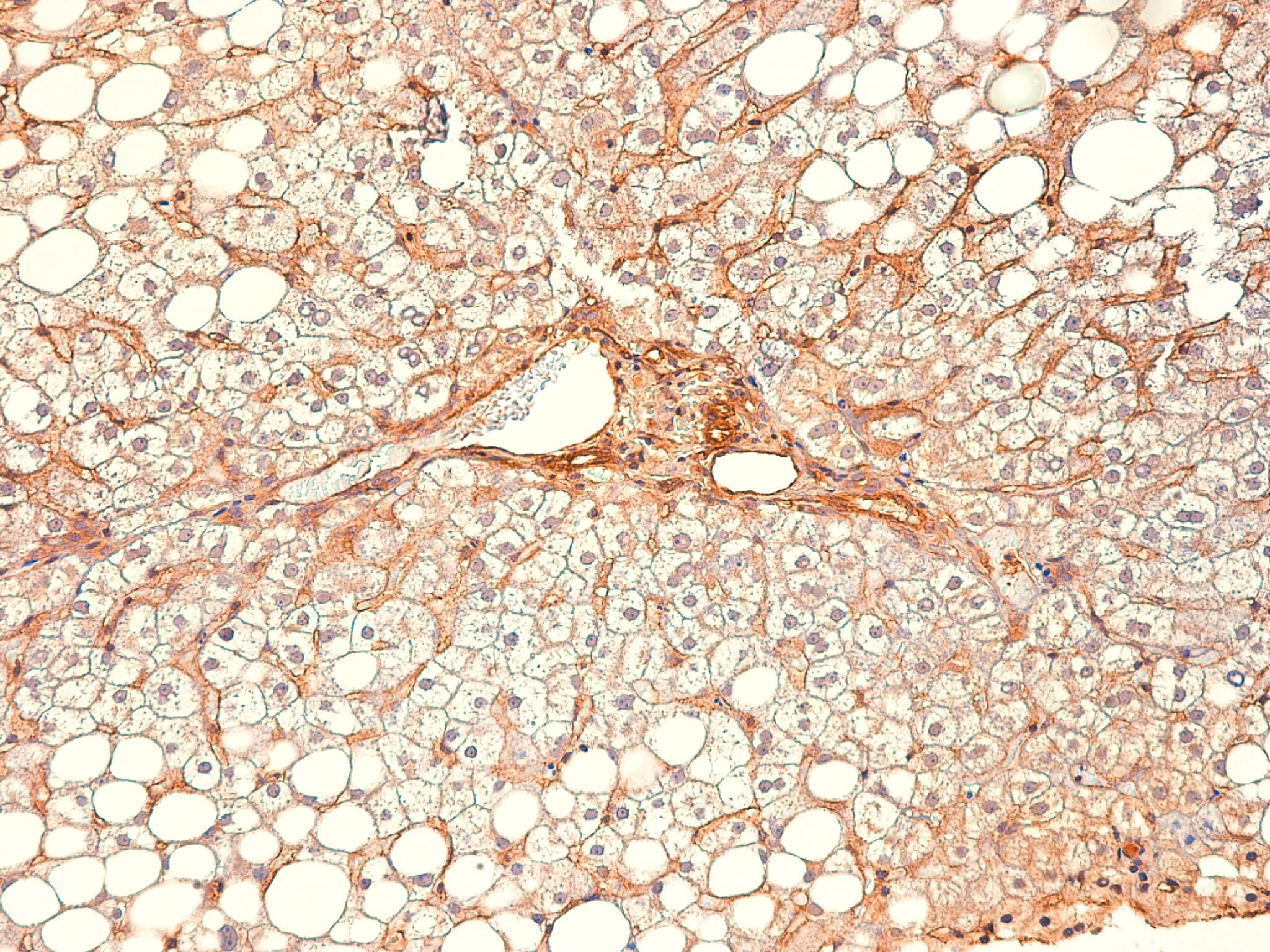

Supplement: Supplementary file 12 — Microscopy images for Supplementary Fig. 7. [file 42255_2021_518_MOESM12_ESM.zip › PSD3 HOM 3.jpg]

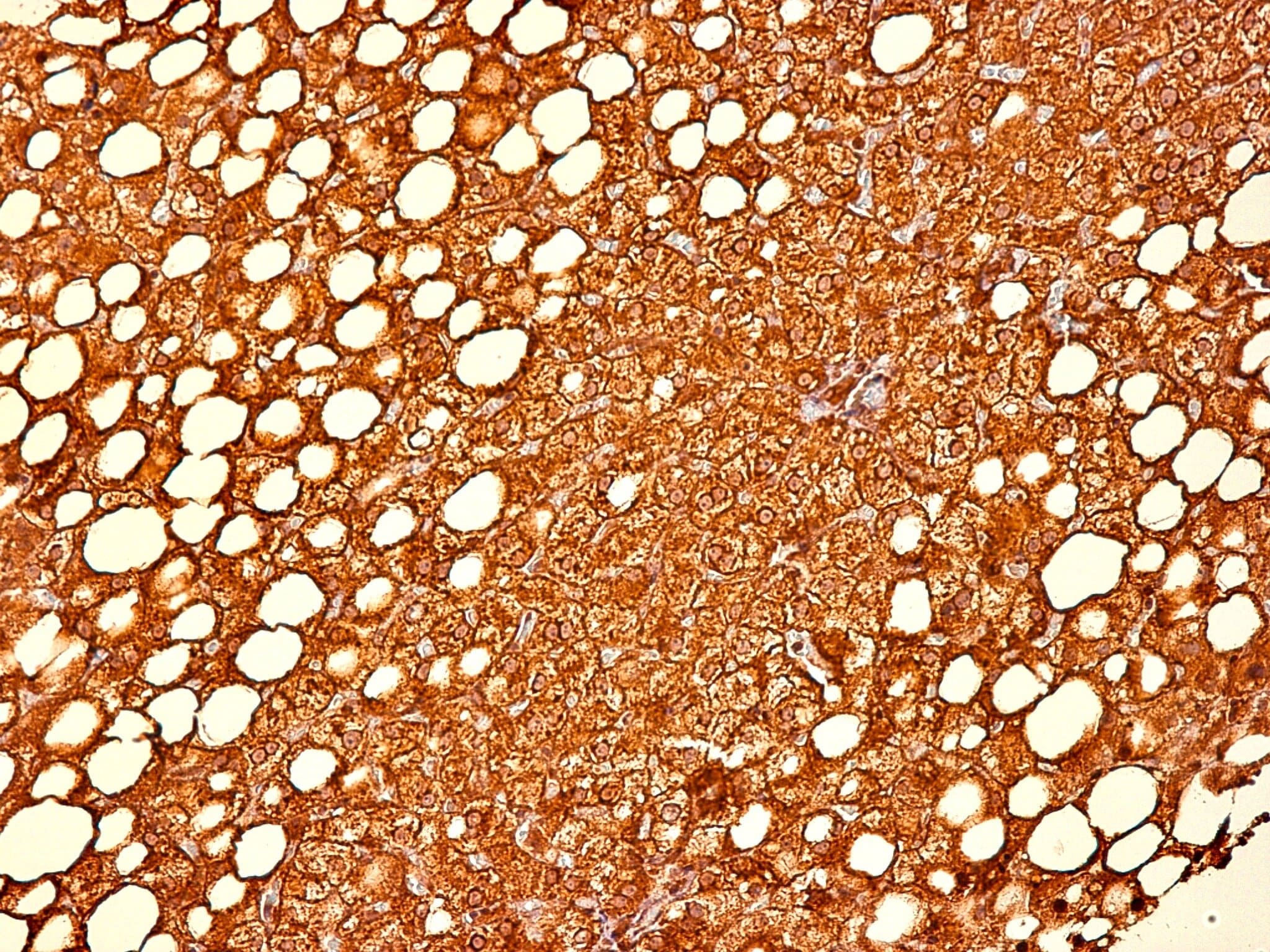

Supplement: Supplementary file 12 — Microscopy images for Supplementary Fig. 7. [file 42255_2021_518_MOESM12_ESM.zip › PSD3 HOM 3_ARF6.jpg]

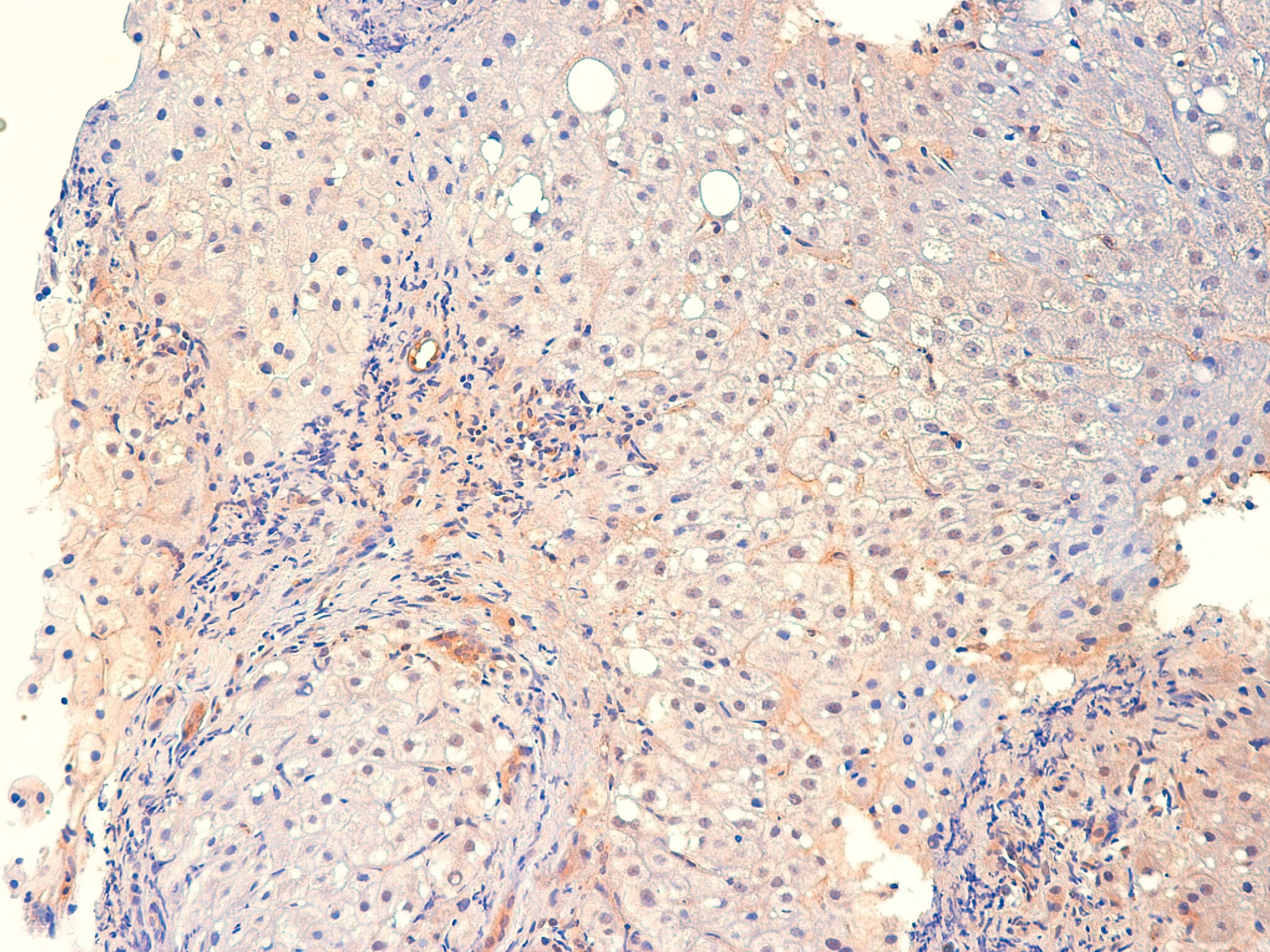

Supplement: Supplementary file 12 — Microscopy images for Supplementary Fig. 7. [file 42255_2021_518_MOESM12_ESM.zip › PSD3 HOM 4.jpg]

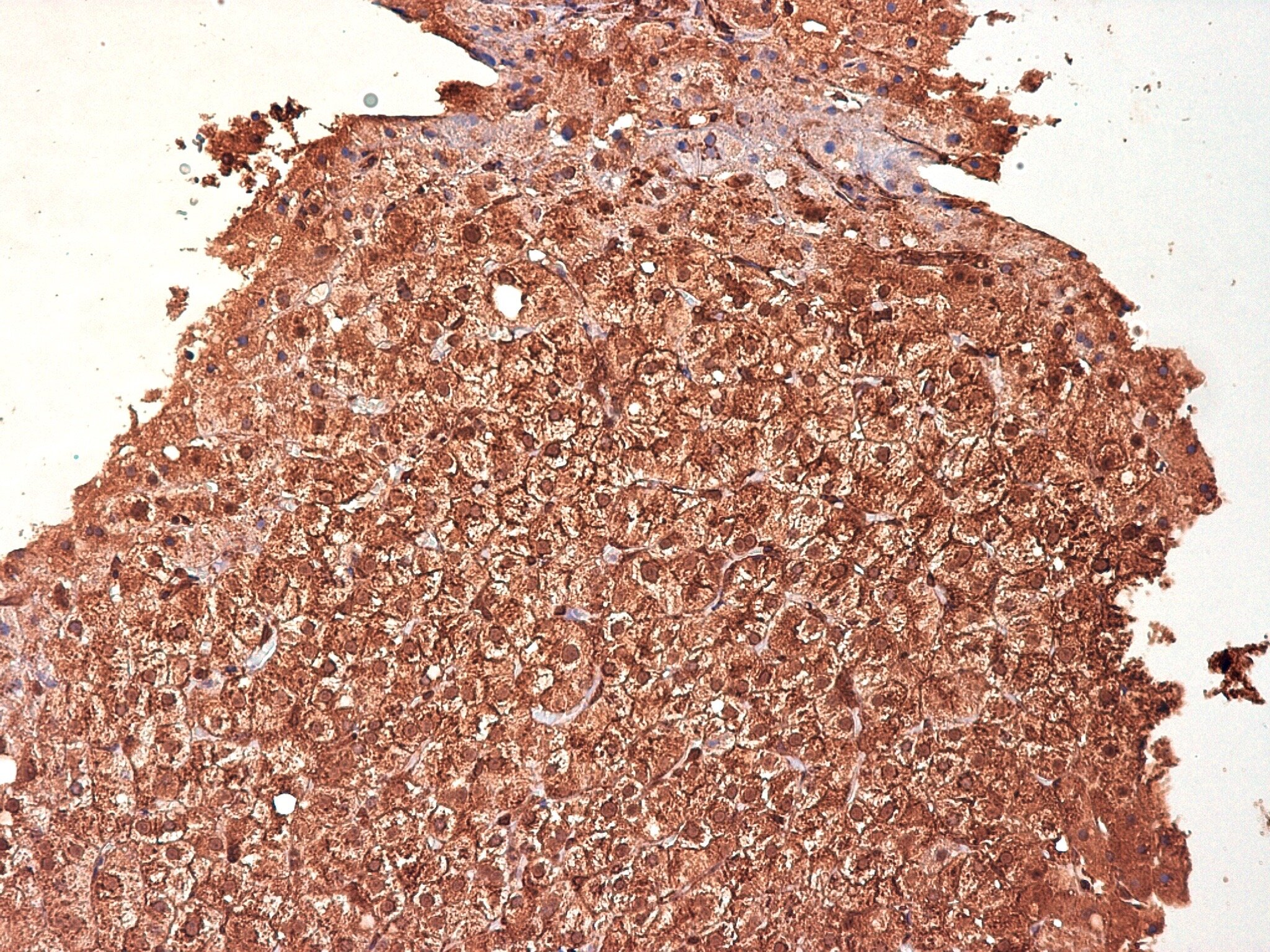

Supplement: Supplementary file 12 — Microscopy images for Supplementary Fig. 7. [file 42255_2021_518_MOESM12_ESM.zip › PSD3 HOM 4_ARF6.jpg]

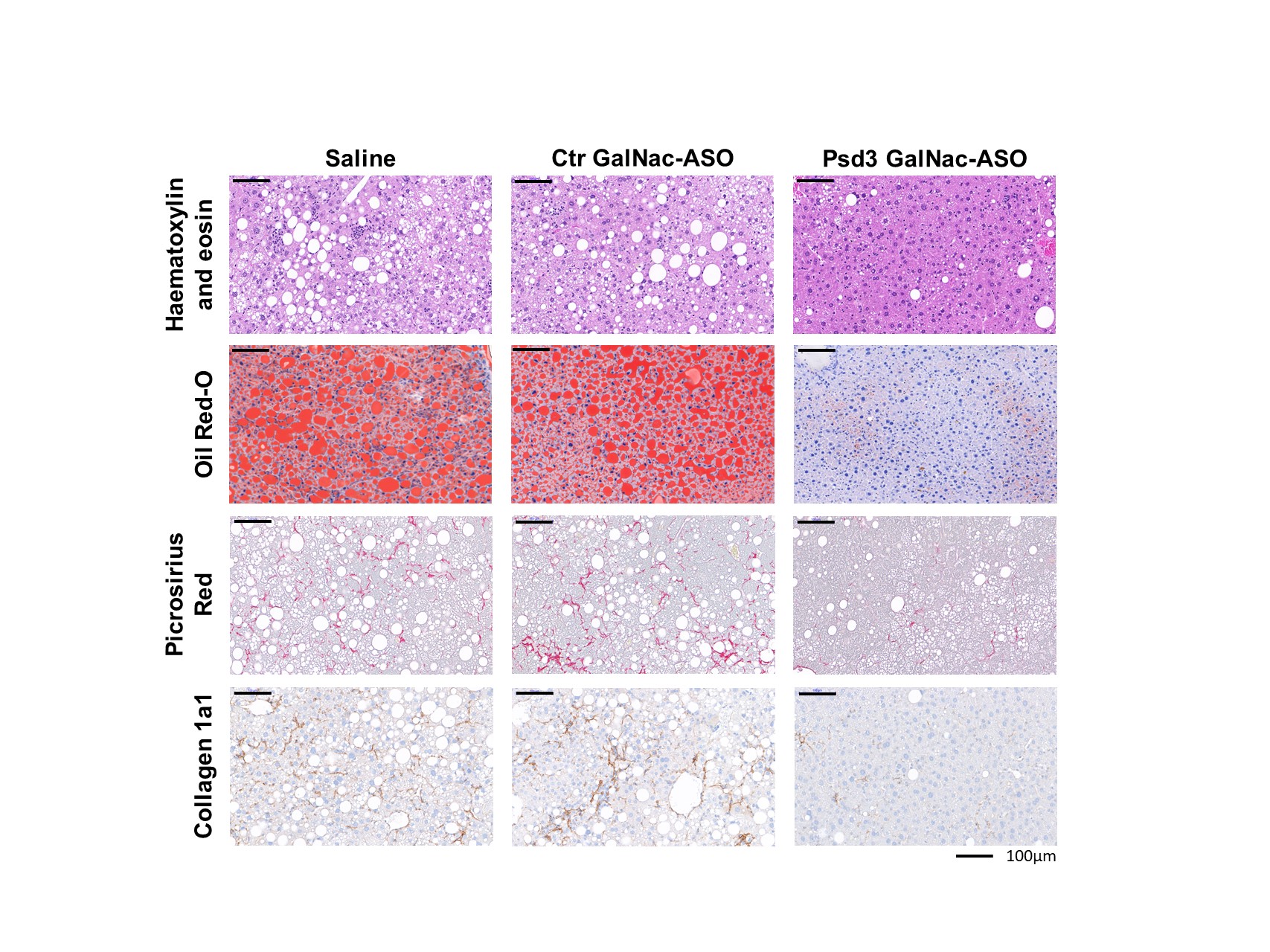

Supplement: Source Data Fig. 8 — Microscopy image combined. [file 42255_2021_518_MOESM23_ESM.jpg]
