# Supplementary material for: PSD3 downregulation confers protection against fatty liver disease
Source: Nat Metab. 2022 Jan 31;4(1):60–75. doi: 10.1038/s42255-021-00518-0 (PMC8803605; doi:10.1038/s42255-021-00518-0)
Supplement: Source Data Fig. 3 — Unprocessed western blot. [file 42255_2021_518_MOESM15_ESM.pdf]

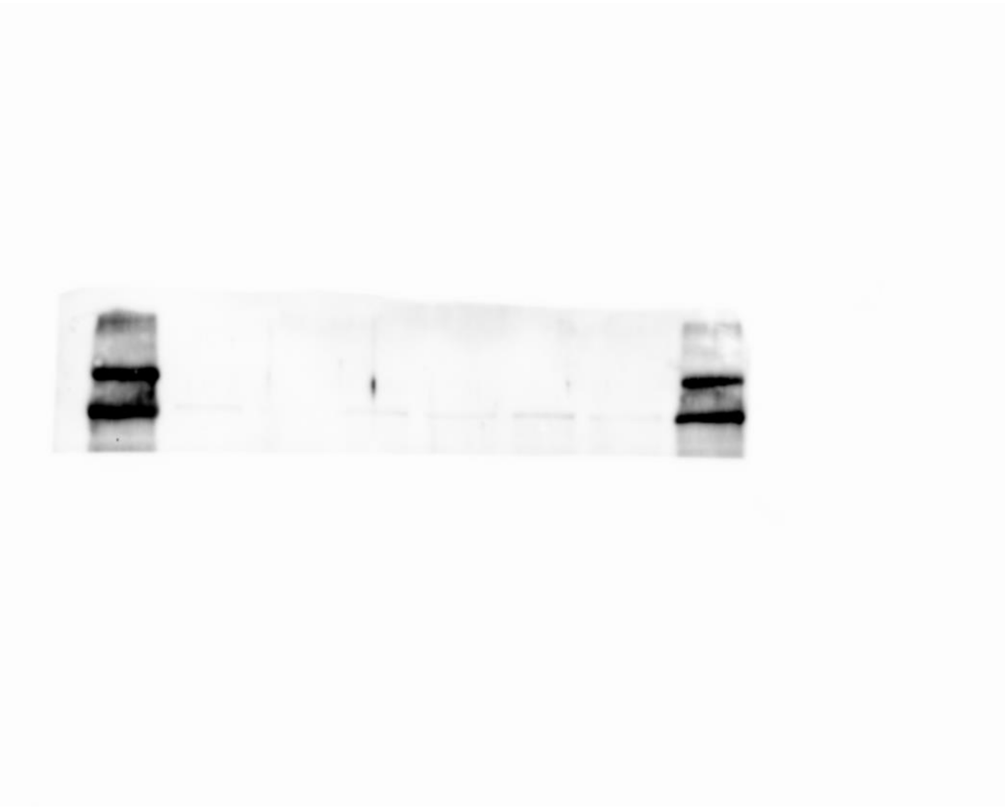

PSD3 antibody

Blot obtained with ECL detection (regular)

Sample order

1. 186L (2%FBS)
2. 186T (2%FBS)
3. 186L(10µM Oleic acid)
4. 186T (10µM oleic acid)
5. 186L (25µM oleic acid)
6. 186T (25µM oleic acid)

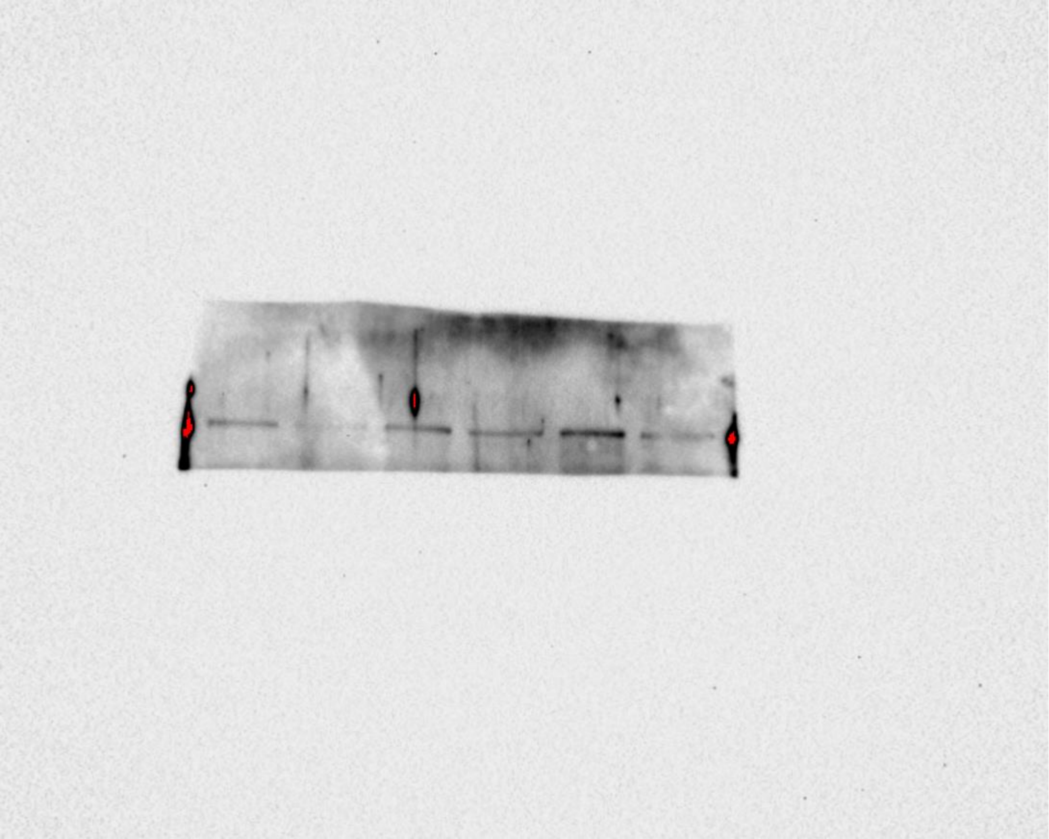

PSD3 antibody

Blot obtained with ECL detection (supersensitive)

Sample order

1. 186L (2%FBS)
2. 186T (2%FBS)
3. 186L(10µM Oleic acid)
4. 186T (10µM oleic acid)
5. 186L (25µM oleic acid)
6. 186T (25µM oleic acid)

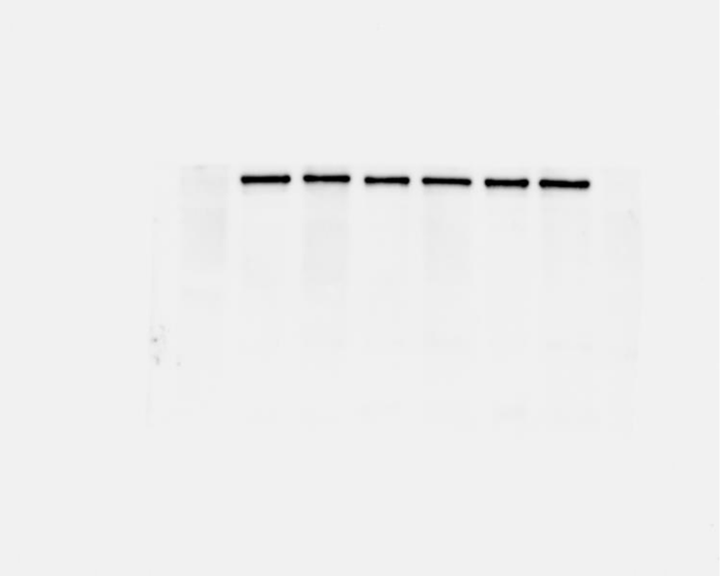

Calnexin antibody

Blot obtained with ECL detection (regular)

Sample order

1. 186L (2%FBS)
2. 186T (2%FBS)
3. 186L(10µM Oleic acid)
4. 186T (10µM oleic acid)
5. 186L (25µM oleic acid)
6. 186T (25µM oleic acid)
