# Supplementary material for: PSD3 downregulation confers protection against fatty liver disease
Source: Nat Metab. 2022 Jan 31;4(1):60–75. doi: 10.1038/s42255-021-00518-0 (PMC8803605; doi:10.1038/s42255-021-00518-0)
Supplement: Source Data Fig. 4 — Unprocessed western blot. [file 42255_2021_518_MOESM17_ESM.pdf]

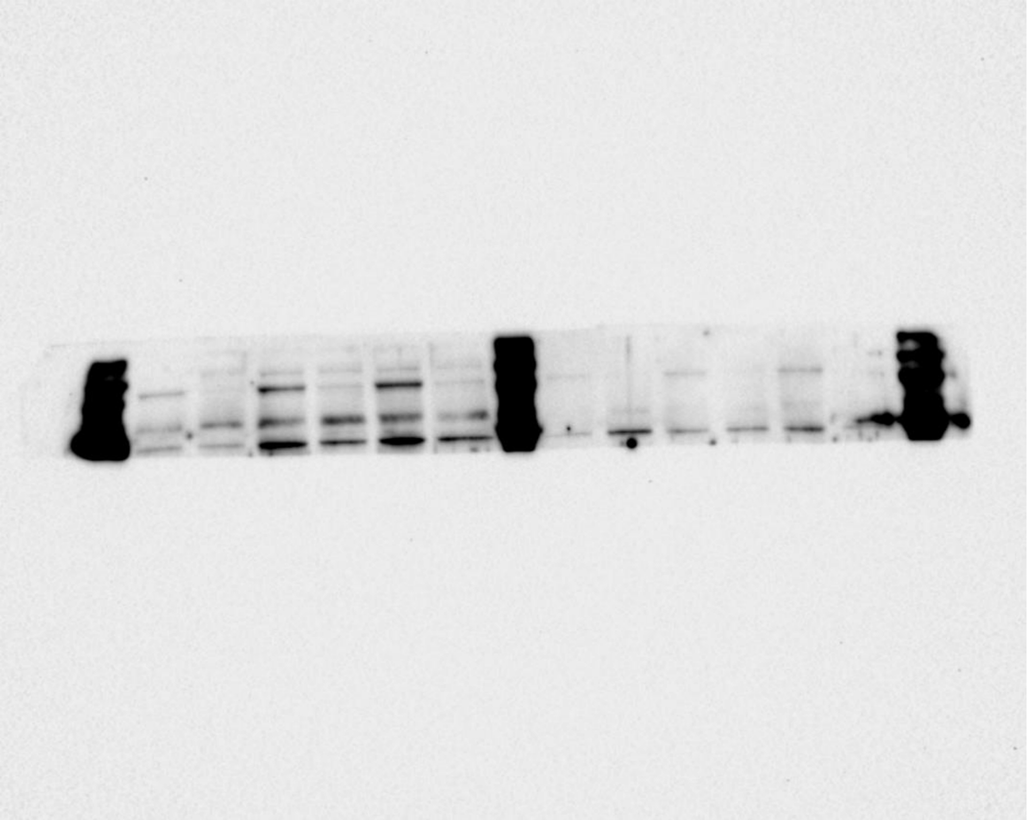

PSD3 antibody

Blot visualized with ECL detection (supersensitive)

Sample order

1. 186L SCR (2%FBS treated)
2. 186L PSD3 siRNA (2%FBS treated)
3. 186L SCR (10µM OA treated)
4. 186L PSD3 siRNA (10µM OA treated)
5. 186L SCR (25µM OA treated)
6. 186L PSD3 siRNA (25µM OA treated)
7. 186T SCR (2%FBS treated)
8. 186T PSD3 siRNA (2%FBS treated)
9. 186T SCR (10µM OA treated)
10. 186T PSD3 siRNA (10µM OA treated)
11. 186T SCR (25µM OA treated)
12. 186T PSD3 siRNA (25µM OA treated)

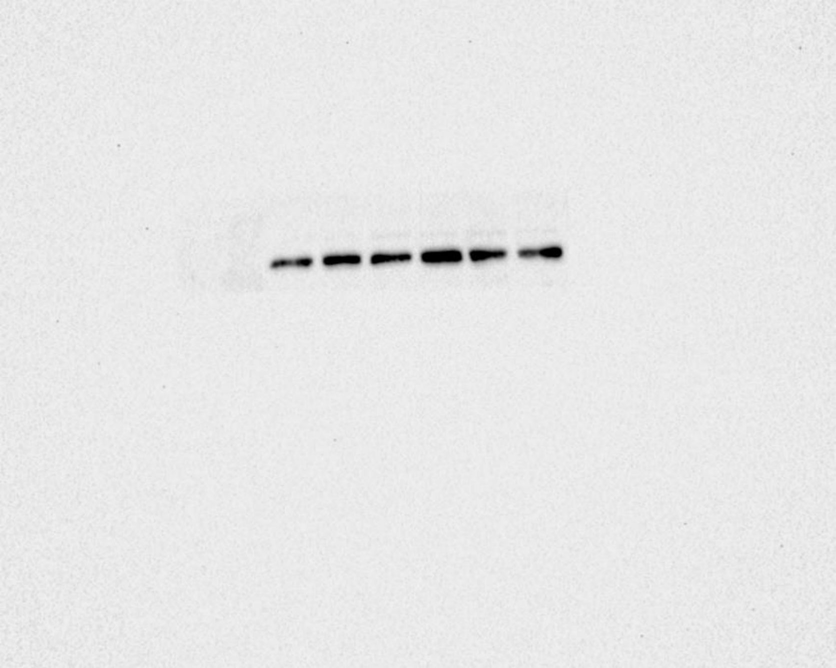

CALNEXIN antibody

Blot visualized with ECL detection (regular)

Sample order

1. 186L SCR (2%FBS treated)
2. 186L PSD3 siRNA (2%FBS treated)
3. 186L SCR (10µM OA treated)
4. 186L PSD3 siRNA (10µM OA treated)
5. 186L SCR (25µM OA treated)
6. 186L PSD3 siRNA (25µM OA treated)

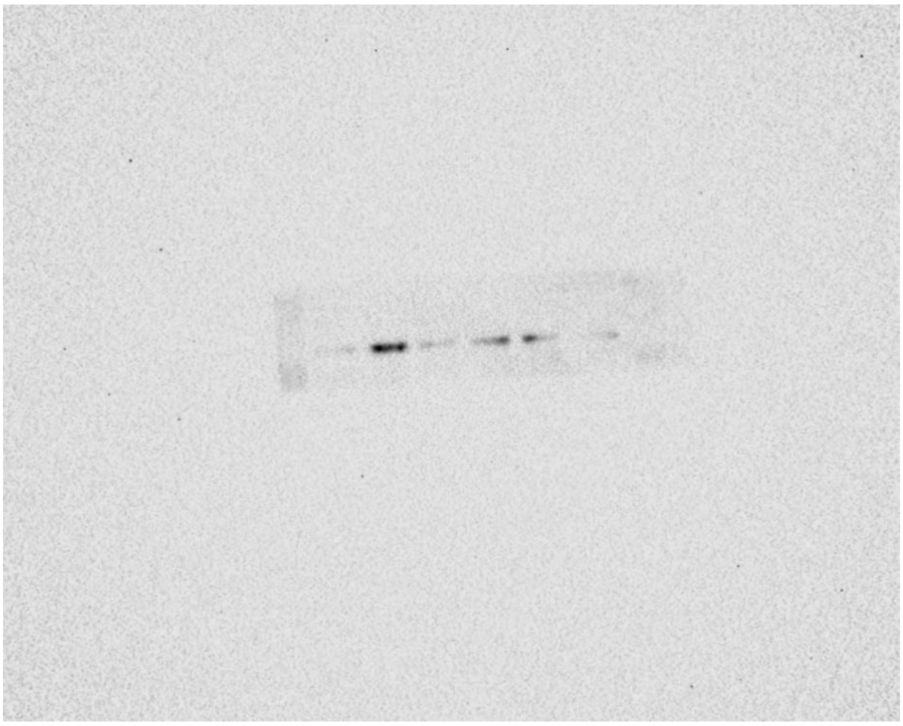

CALNEXIN antibody

Blot visualized with ECL detection (regular)

Sample order

1. 186T SCR (2%FBS treated)
2. 186T PSD3 siRNA (2%FBS treated)
3. 186T SCR (10µM OA treated)
4. 186T PSD3 siRNA (10µM OA treated)
5. 186T SCR (25µM OA treated)
6. 186T PSD3 siRNA (25µM OA treated)
