# Supplementary material for: PSD3 downregulation confers protection against fatty liver disease
Source: Nat Metab. 2022 Jan 31;4(1):60–75. doi: 10.1038/s42255-021-00518-0 (PMC8803605; doi:10.1038/s42255-021-00518-0)
Supplement: Source Data Fig. 5 — Unprocessed western blot. [file 42255_2021_518_MOESM19_ESM.pdf]

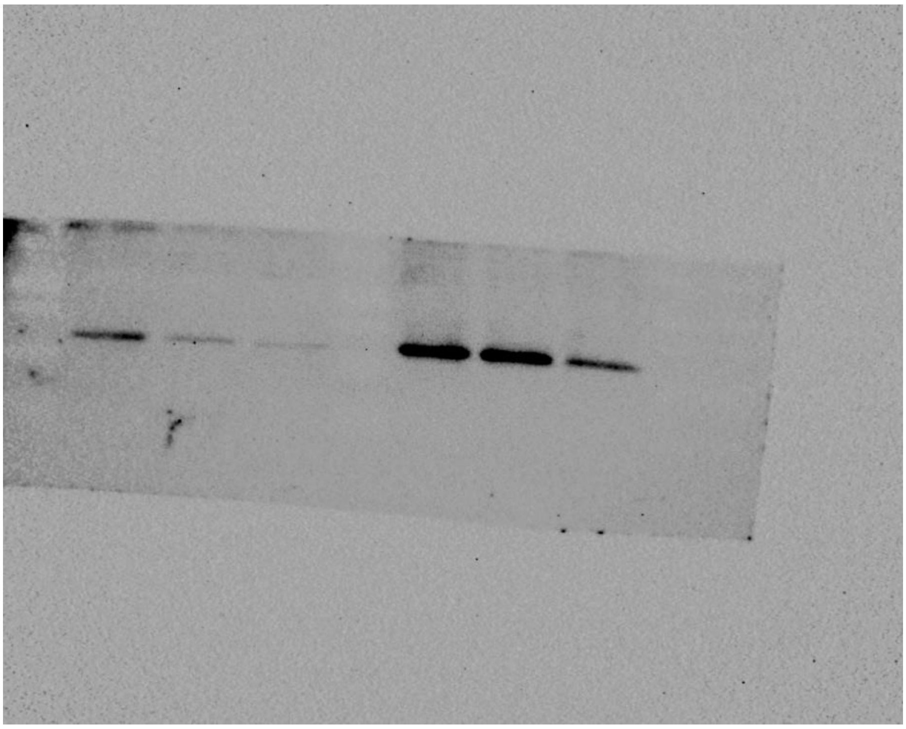

ARF6 antibody

Blot detected with ECL detection (supersensitive)

Sample order: Huh7 cells

1. SCR siRNA (GGA3 beads pull down)
2. PSD3 siRNA(GGA3 beads pull down)
3. ARF6 siRNA(GGA3 beads pull down)
4. SCR siRNA (total)
5. PSD3 siRNA(total)
6. ARF6 siRNA(total)

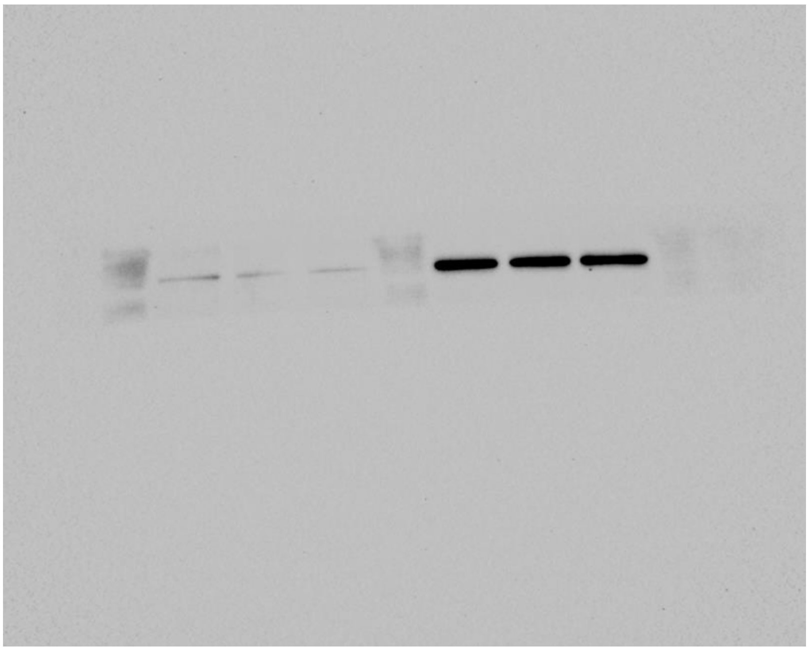

Calnexin

Blot detected with ECL detection (regular)

Sample order: Huh7 cells

1. SCR siRNA(GGA3 beads pull down)
2. PSD3 siRNA(GGA3 beads pull down)
3. ARF6 siRNA(GGA3 beads pull down)
4. SCR siRNA(total)
5. PSD3 siRNA(total)
6. ARF6 siRNA(total)
